# Arbuscular mycorrhizal trees influence the latitudinal beta-diversity gradient of tree communities in forests worldwide

**DOI:** 10.1038/s41467-021-23236-3

**Published:** 2021-05-25

**Authors:** Yonglin Zhong, Chengjin Chu, Jonathan A. Myers, Gregory S. Gilbert, James A. Lutz, Jonas Stillhard, Kai Zhu, Jill Thompson, Jennifer L. Baltzer, Fangliang He, Joseph A. LaManna, Stuart J. Davies, Kristina J. Aderson-Teixeira, David F.R.P. Burslem, Alfonso Alonso, Kuo-Jung Chao, Xugao Wang, Lianming Gao, David A. Orwig, Xue Yin, Xinghua Sui, Zhiyao Su, Iveren Abiem, Pulchérie Bissiengou, Norm Bourg, Nathalie Butt, Min Cao, Chia-Hao Chang-Yang, Wei-Chun Chao, Hazel Chapman, Yu-Yun Chen, David A. Coomes, Susan Cordell, Alexandre A. de Oliveira, Hu Du, Suqin Fang, Christian P. Giardina, Zhanqing Hao, Andrew Hector, Stephen P. Hubbell, David Janík, Patrick A. Jansen, Mingxi Jiang, Guangze Jin, David Kenfack, Kamil Král, Andrew J. Larson, Buhang Li, Xiankun Li, Yide Li, Juyu Lian, Luxiang Lin, Feng Liu, Yankun Liu, Yu Liu, Fuchen Luan, Yahuang Luo, Keping Ma, Yadvinder Malhi, Sean M. McMahon, William McShea, Hervé Memiaghe, Xiangcheng Mi, Mike Morecroft, Vojtech Novotny, Michael J. O’Brien, Jan den Ouden, Geoffrey G. Parker, Xiujuan Qiao, Haibao Ren, Glen Reynolds, Pavel Samonil, Weiguo Sang, Guochun Shen, Zhiqiang Shen, Guo-Zhang Michael Song, I-Fang Sun, Hui Tang, Songyan Tian, Amanda L. Uowolo, María Uriarte, Bin Wang, Xihua Wang, Youshi Wang, George D. Weiblen, Zhihong Wu, Nianxun Xi, Wusheng Xiang, Han Xu, Kun Xu, Wanhui Ye, Mingjian Yu, Fuping Zeng, Minhua Zhang, Yingming Zhang, Li Zhu, Jess K. Zimmerman

**Affiliations:** 1 Department of Ecology, State Key Laboratory of Biocontrol and School of Life Sciences, Sun Yat-sen University,; 2 Department of Biology, Washington University in St. Louis, St. Louis, MO USA; 3 Department of Environmental Studies, University of California, Santa Cruz, CA USA; 4 Wildland Resources Department, Utah State University, Logan, UT USA; 5 Swiss Federal Research Institute for Forest, Snow and Landscape Research WSL, Forest Resources and Management, Birmensdorf, Switzerland; 6 UK Centre for Ecology & Hydrology Bush Estate, Midlothian, UK; 7 Biology Department, Wilfrid Laurier University, Waterloo, ON Canada; 8 Department of Renewable Resources, University of Alberta, Edmonton, AB Canada; 9 ECNU-Alberta Joint Lab for Biodiversity Study, Tiantong National Station for Forest Ecosystem Research, East China Normal University,; 10 Zhejiang Tiantong Forest Ecosystem National Observation and Research Station, School of Ecology and Environmental Sciences, East China Normal University,; 11 Department of Biological Sciences, Marquette University, Milwaukee, WI USA; 12 Forest Global Earth Observatory, Smithsonian Tropical Research Institute, Washington, DC USA; 13 Conservation Ecology Center, Smithsonian Conservation Biology Institute, National Zoological Park, Front Royal, VA USA; 14 School of Biological Sciences, University of Aberdeen, Aberdeen, UK; 15 Center for Conservation and Sustainability, Smithsonian Conservation Biology Institute, National Zoological Park, Washington, DC USA; 16 International Master Program of Agriculture, National Chung Hsing University, https://www.nchu.edu.tw/en-index; 17 CAS Key Laboratory of Forest Ecology and Management, Institute of Applied Ecology, Chinese Academy of Sciences, http://english.iae.cas.cn/; 18 CAS Key Laboratory for Plant Diversity and Biogeography of East Asia, Kunming Institute of Botany, Chinese Academy of Sciences, http://english.kib.cas.cn/; 19 Harvard Forest, Harvard University, Petersham, MA USA; 20 College of Forestry and Landscape Architecture, South China Agricultural University, https://english.scau.edu.cn/; 21 Department of Plant Science and Technology, University of Jos, Jos, Nigeria; 22 The Nigerian Montane Forest Project, Taraba State, Nigeria; 23 School of Biological Sciences, University of Canterbury, Christchurch, New Zealand; 24 Institut de Recherche en Ecologie Tropicale/Centre National de la Recherche Scientifique et Technologique, Libreville, Gabon; 25 School of Biological Sciences, The University of Queensland, St. Lucia, QLD Australia; 26 Centre for Biodiversity and Conservation Science, The University of Queensland, St. Lucia, QLD Australia; 27 CAS Key Laboratory of Tropical Forest Ecology, Xishuangbanna Tropical Botanical Garden, Chinese Academy of Sciences, http://english.xtbg.cas.cn/; 28 Department of Biological Sciences, National Sun Yat-sen University,; 29 Department of Forestry and Natural Resources, National Chiayi University,; 30 Department of Natural Resources and Environmental Studies, National Dong Hwa University,; 31 Department of Plant Sciences, University of Cambridge, Cambridge, UK; 32 Institute of Pacific Islands Forestry, Pacific Southwest Research Station, USDA Forest Service, Hilo, Hawaii USA; 33 Departamento Ecologia, Universidade de São Paulo, Instituto de Biociências, Cidade Universitária, São Paulo, SP Brazil; 34 Key Laboratory of Agro-ecological Processes in Subtropical Region, Institute of Subtropical Agriculture, Chinese Academy of Sciences, http://english.isa.cas.cn/; 35 School of Ecology and Environment, Northwestern Polytechnical University, http://en.nwpu.edu.cn/; 36 Department of Plant Sciences, University of Oxford, Oxford, UK; 37 Department of Ecology and Evolutionary Biology, University of California, Los Angeles, Los Angeles, CA USA; 38 Department of Forest Ecology, Silva Tarouca Research Institute, Brno, Czech Republic; 39 Wildlife Ecology and Conservation Group, Wageningen University and Research, Wageningen, The Netherlands; 40 Key Laboratory of Aquatic Botany and Watershed Ecology, Wuhan Botanical Garden, Chinese Academy of Sciences, http://english.wbg.cas.cn/; 41 Center for Ecological Research, Northeast Forestry University, http://en.nefu.edu.cn/; 42 Department of Botany, National Museum of Natural History, Washington, DC USA; 43 Wilderness Institute and Department of Forest Management, University of Montana, Missoula, MT USA; 44 Guangxi Key Laboratory of Plant Conservation and Restoration Ecology in Karst Terrain, Guangxi Institute of Botany, Guangxi Zhuang Autonomous Region and Chinese Academy of Sciences, http://english.gxib.cn/; 45 Research Institute of Tropical Forestry, Chinese Academy of Forestry, http://ritf.caf.ac.cn/; 46 Key Laboratory of Vegetation Restoration and Management of Degraded Ecosystems, South China Botanical Garden, Chinese Academy of Sciences, http://english.scbg.ac.cn/; 47 The Administrative Bureau of Naban River Watershed National Nature Reserve, http://www.xsbn.gov.cn/nbhbhq/nbhbhq.dhtml; 48 Heilongjiang Key Laboratory of Forest Ecology and Forestry Ecological Engineering, Heilongjiang Forestry Engineering and Environment Institute, http://www.hljifee.org.cn/; 49 Guangdong Chebaling National Nature Reserve, https://cbl.elab.cnic.cn/; 50 State Key Laboratory of Vegetation and Environmental Change, Institute of Botany, Chinese Academy of Sciences, http://english.ib.cas.cn/; 51 Environmental Change Institute, School of Geography and the Environment, University of Oxford, Oxford, UK; 52 Smithsonian Environmental Research Center, Edgewater, MD USA; 53 Natural England, York, UK; 54 Biology Center of the Czech Academy of Sciences, Institute of Entomology and the University of South Bohemia, Ceske Budejovicve, Czech Republic; 55 Área de Biodiversidad y Conservación, Universidad Rey Juan Carlos, Móstoles, Madrid, Spain; 56 Forest Ecology and Management Group, Wageningen University, Wageningen, The Netherlands; 57 Forest Ecology Group, Smithsonian Environmental Research Center, Edgewater, MD USA; 58 Southeast Asia Rainforest Research Partnership, Danum Valley Field Centre, Lahad Datu, Sabah Malaysia; 59 College of Life and Environmental Science, Minzu University of China,; 60 Department of Soil and Water Conservation, National Chung Hsing University,; 61 Department of Ecology, Evolution and Environmental Biology, Columbia University, New York, NY USA; 62 Department of Plant & Microbial Biology, University of Minnesota, St. Paul, MN USA; 63 Yunnan Lijiang Forest Ecosystem National Observation and Research Station, Kunming Instituted of Botany, Chinese Academy of Sciences, http://english.kib.cas.cn/; 64 MOE Key Laboratory of Biosystems Homeostasis & Protection, College of Life Sciences, Zhejiang University, http://www.zju.edu.cn/english/; 65 Department of Environmental Sciences, University of Puerto Rico, San Juan, PR USA

**Keywords:** Biodiversity, Biogeography

## Abstract

Arbuscular mycorrhizal (AM) and ectomycorrhizal (EcM) associations are critical for host-tree performance. However, how mycorrhizal associations correlate with the latitudinal tree beta-diversity remains untested. Using a global dataset of 45 forest plots representing 2,804,270 trees across 3840 species, we test how AM and EcM trees contribute to total beta-diversity and its components (turnover and nestedness) of all trees. We find AM rather than EcM trees predominantly contribute to decreasing total beta-diversity and turnover and increasing nestedness with increasing latitude, probably because wide distributions of EcM trees do not generate strong compositional differences among localities. Environmental variables, especially temperature and precipitation, are strongly correlated with beta-diversity patterns for both AM trees and all trees rather than EcM trees. Results support our hypotheses that latitudinal beta-diversity patterns and environmental effects on these patterns are highly dependent on mycorrhizal types. Our findings highlight the importance of AM-dominated forests for conserving global forest biodiversity.

## Abstract (web summary)

The relationship of mycorrhizal associations with latitudinal gradients in tree beta-diversity is unexplored. Using a global dataset approach, this study examines how trees with arbuscular mycorrhizal and ectomycorrhizal associations contribute to latitudinal beta-diversity patterns and the environmental controls of these patterns.

## Introduction

Variation in community composition (beta-diversity) provides key insights into mechanisms of community assembly and biodiversity maintenance across local and regional scales^1–4^. Total beta-diversity arises from two components: species turnover (i.e., species replacement) and species nestedness (i.e., where sites with fewer species tend to be subsets of sites with more species)^5,6^. These two beta-diversity components are closely associated with various ecological, historical, and evolutionary processes. Species turnover usually occurs among communities with high speciation rates, dispersal limitation, ecological drift, or habitat heterogeneity^7–9^. In contrast, species nestedness often occurs in communities with nested habitat conditions and selective extinction or selective recolonization across environmental gradients^5,10,11^. Studies of the latitudinal gradient in beta-diversity have yielded mixed results, with U-shaped, unimodal, positive, negative, or neutral trends being reported in the literature^3,12–15^. In particular, some studies partitioning beta-diversity have found that species turnover decreases with increasing latitude, while species nestedness increases^16,17^. However, the latitudinal patterns of these two beta-diversity components of trees have not been extensively explored, particularly at the global scale. Key gaps remain in our understanding how local biotic interactions contribute to patterns of beta-diversity across large-scale gradients^18^. In particular, the importance of mutualistic biotic interactions in determining latitudinal gradients in beta-diversity of trees remains largely unknown.

Mutualistic interactions among plants and mycorrhizal fungi may be one of the most important, but least studied, biotic interactions that contribute to patterns of plant beta-diversity across latitudes. Arbuscular mycorrhizal fungi (AM fungi) and ectomycorrhizal fungi (EcM fungi) form symbioses with more than 80% of terrestrial plants globally^19,20^. Although plant-mycorrhizal associations are ubiquitous, the geographic variation in relative abundance, diversity, and distributions of AM and EcM plant species may influence patterns of plant beta-diversity via several ecological and evolutionary mechanisms^21–23^.

Mycorrhizal associations may influence latitudinal variation in beta-diversity through differences in how AM and EcM plants adapt to habitat conditions (i.e., habitat adaptation)^24–27^. Greater adaptation of species to specific habitat conditions may enhance speciation rates and reduce extinction rates^25,28^. AM plants and EcM plants differ in their soil nutrient uptake capacities and trade-offs of carbon cost. AM plants are superior competitors for available inorganic nutrients compared to EcM plants. EcM plants, however, have greater capacity to mineralize nutrients from organic matter directly than AM plants^21,29^. Furthermore, AM and EcM plants respond differently to climate conditions, with AM plants preferring to wet and warm conditions, while EcM plants are better adapted to dry and cold conditions. Thus, the warm, wet, and aseasonal tropical regions with high decomposition rates are primarily dominated by AM trees despite of some exceptional EcM-tree-dominated forests, whereas the dry, cold, and seasonal temperate regions are primarily dominated by EcM trees^24,25^. This latitudinal gradient in habitat adaptation may provide AM trees in tropical regions with higher speciation rates but lower selective extinction rates than in temperate regions^23–25,30,31^. Such higher speciation rates and/or lower extinction rates of trees may in turn increase total beta-diversity and species turnover in the tropics by increasing the number of species in the regional species pool^15,32^. Lower selective extinction rates may decrease species loss across environmental gradients (i.e., species nestedness) in the tropics^5^. In contrast, EcM trees in temperate regions may have higher speciation rates, lower extinction rates, and a larger species pool than in tropical regions due to habitat adaptation. This may lead to higher species turnover and lower species nestedness of EcM trees in temperate than in tropical regions^5,33–36^.

Mycorrhizal associations may also influence forest beta-diversity via differences in plant-soil feedbacks (PSFs) between AM and EcM trees^26,27,34–37^. For example, AM trees generally perform better in soils from heterospecifics than from conspecifics (i.e., a negative PSF), whereas EcM trees perform better in soils from conspecifics than from heterospecifics (i.e., a positive PSF)^26,38^. Differences in PSFs between AM and EcM tree species may be the result of less protection of roots of AM trees from soil pathogens due to the lack of mantle formation on the host root surface^26,39,40^, or because EcM trees are better able to mine nutrients from soil organic matter arising from host trees^27^. In communities dominated by AM trees, negative PSFs are predicted to reduce the performance of conspecific individuals of abundant species and promote the persistence of rare species^26,41^. In turn, rare species that are habitat specialists may increase species turnover by promoting uniqueness of species composition among localities^33^. Negative PSFs may also increase species turnover by limiting the spatial extent of species ranges, resulting in more species unique to different localities and fewer species found in common among localities^34,36^. Thus, the negative PSFs common among AM trees may promote species turnover. In contrast, positive PSFs in more EcM-dominated communities could promote the performance of conspecific individuals of abundant species and inhibit rare species, leading to selective loss of rare species due to competitive exclusion^26,35^, which generates patterns of species nestedness. The strength of conspecific negative-density dependence and the prevalence of AM trees have been demonstrated to decrease with increasing latitude^42,43^. The weaker negative PSFs and lower predominance of AM plants may lead to a decrease in species turnover with increasing latitude^33,34,36^. In contrast, species nestedness of EcM plants may increase with latitude due to the greater prevalence of EcM species with positive PSFs^5,24–26,35^. Despite widespread interest in how mycorrhizal associations influence host population dynamics, biodiversity maintenance, and ecosystem functioning at various spatial scales^25–27^, the effects of mycorrhizal associations on the latitudinal gradient in tree beta-diversity remain unexplored.

**Fig. 1:**
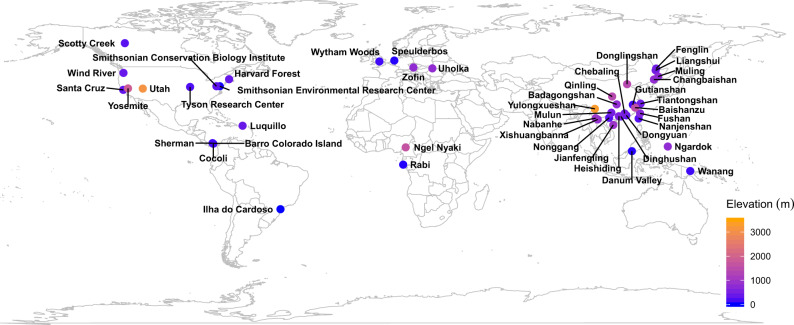
Global distribution of 45 forest plots. Plots range in size from 2.1 ha (Nanjenshan) to 60 ha (Jianfengling) and in latitude from 21.5 °S (Ilha do Cardoso, Brasil) to 61.3 °N (Scotty Creek, Canada), covering all continents with forests (i.e., Asia, Africa, Europe, South America, North America, and Oceania).

In this study, we examine how mycorrhizal associations and environmental factors (climate and topography) may influence the latitudinal gradient in beta-diversity of forest trees. Using data from 45 large, stem-mapped forest plots across the globe (Fig. 1), we calculate total beta-diversity, the abundance-weighted species turnover component (hereafter species turnover), and the abundance-weighted species nestedness component (hereafter species nestedness) for AM trees, EcM trees, and all trees (a combination of AM trees, EcM trees, and other trees). We expect that total beta-diversity and species turnover decrease with increasing latitude, whereas species nestedness increases. In particular, we test three hypotheses: (1) Latitudinal gradients in beta-diversity and its components are highly dependent on mycorrhizal types of host trees; (2) Latitudinal gradients in beta-diversity and its components are mainly shaped by environmental rather than spatial variables; and (3) Effects of environmental and spatial variables on beta-diversity and its components are highly dependent on types of mycorrhizal associations. We find that latitudinal beta-diversity patterns and environmental effects on these patterns are highly dependent on mycorrhizal types. AM rather than EcM trees predominantly contribute to decreasing total beta-diversity and turnover and increasing nestedness with increasing latitude.

## Results

### Latitudinal beta-diversity patterns contributed by AM trees

**Fig. 2:**
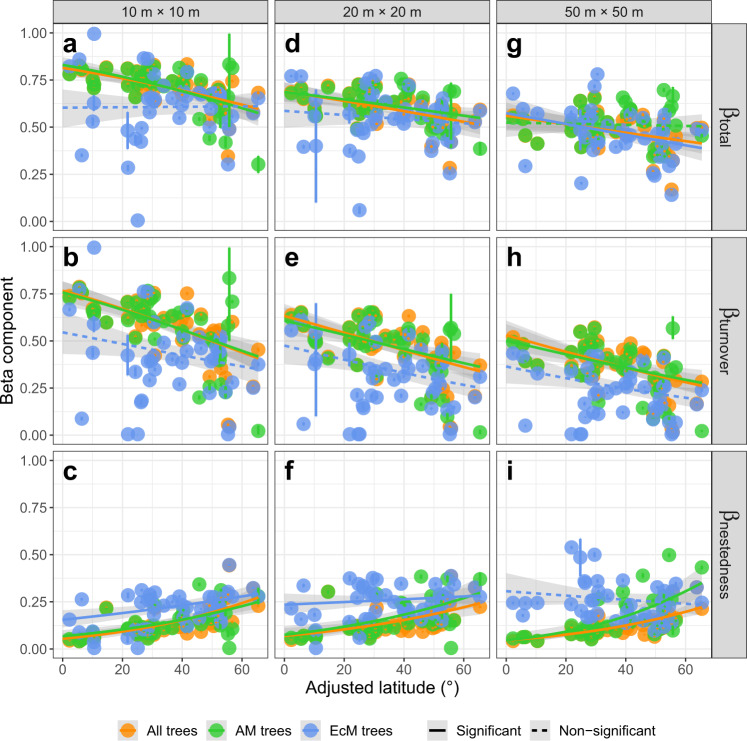
Latitudinal gradients in tree beta-diversity. Total beta-diversity, species turnover, and species nestedness of all trees, AM trees, and EcM trees across latitudes at quadrat scales of 10 m × 10 m (**a**–**c**), 20 m × 20 m (**d**–**f**), and 50 m × 50 m (**g**–**i**). Orange points represent total beta-diversity and its two components (species turnover & nestedness) of all trees and orange lines represent their latitudinal patterns. Green points represent total beta-diversity and its components of AM trees and green lines represent their latitudinal patterns. Blue points represent total beta-diversity and its components of EcM trees and blue lines represent their latitudinal patterns. Points are the mean values and the error bars are the 95% confidence intervals, estimated using the non-parametric bootstrapping method (*n* = 200). In total, 200 replicates of average pairwise beta-diversity and its components were calculated based on 30, 15, and 15 randomly sampled quadrats of 10 m × 10 m, 20 m × 20 m, and 50 m × 50 m from each forest plot, respectively. Solid lines indicate significant relationships with latitude whereas dashed lines indicate non-significant relationships fitted using the beta regression. The error bands (shaded areas) are the 95% confidence intervals of the fitted relationships, with sample size *n* = 45 for all trees, *n* = 44 for AM trees, and *n* = 43 for EcM trees at the 10 m × 10 m scale; with *n* = 45 for all trees, *n* = 44 for AM trees, and *n* = 44 for EcM trees at the 20 m × 20 m scale; and with *n* = 41 for all trees, *n* = 40 for AM trees, and *n* = 41 for EcM trees at the 50 m × 50 m scale.

Latitudinal patterns of beta-diversity of AM trees mirrored patterns of beta-diversity of all trees (Fig. 2). Total beta-diversity and species turnover of all trees and of AM trees generally decreased with increasing latitude (Fig. 2). The only exception was total beta-diversity of AM trees at the 50 m × 50 m quadrat scale, which was not significantly related to latitude (Beta regression: Pseudo *R*^*2*^ = 0.002, *P* = 0.765; Fig. 2g). Species nestedness of all trees, AM trees, and EcM trees increased with latitude at all quadrat scales with the exception of species nestedness of EcM trees at the 50 m × 50 m quadrat scale (Fig. 2c, f, i). In contrast, total beta-diversity and species turnover of EcM trees were generally unrelated to latitude (Fig. 2), with one exception: total beta-diversity of EcM trees decreased with increasing latitude at the 50 m × 50 m quadrat scale (Beta regression: Pseudo *R*^*2*^ = 0.123, *P* = 0.016; Fig. 2g). When AM trees were excluded from all trees, the latitudinal trends of total beta-diversity, species turnover, and species nestedness disappeared or highly deviated from those of all trees, whereas these patterns remained unchanged when excluding EcM trees (from all trees) (Supplementary Fig. 1).

The simulation experiment (Supplementary Methods) to remove the effect of disproportionate latitudinal distributions of abundance or species richness between AM and EcM trees by sampling equal numbers of individuals or equal numbers of species of AM and EcM trees also showed that the latitudinal patterns of total beta-diversity and species turnover of AM trees paralleled those of all trees, whereas those of EcM trees were not correlated with latitudes, although the patterns for species nestedness were mixed. These simulated results indicate that the contribution of AM trees to the latitudinal beta-diversity patterns of all trees was not driven simply by latitudinal variation in the abundance or species richness of AM trees (Supplementary Figs. 2 and 3). Together, these observed and simulated results indicate that AM trees are predominantly responsible for observed latitudinal patterns of total beta-diversity, species turnover, and species nestedness of all trees.

The relative contribution of species turnover to total beta-diversity for both AM trees and all trees decreased with increasing latitude, while the relative contribution of species nestedness increased (Supplementary Fig. 4a, b, 4d, e, and 4g, h). In contrast, the contributions of species turnover and nestedness of EcM trees remained relatively unchanged across latitude except at the scale of 10 m × 10 m (Supplementary Fig. 4c, f, i). In general, the relative contribution of species turnover was significantly larger than that of species nestedness for AM trees, EcM trees, and all trees across quadrat sizes (Mann–Whitney U test: *P* < 0.05; Supplementary Fig. 4a–e and 4g, h), with one exception: the differences between contributions of species turnover and species nestedness were not significant for EcM trees at the 20 m × 20 m quadrat size (Mann–Whitney U test: W = 1029, *P* = 0.614; Supplementary Fig. 4f).

### Effects of environmental and spatial variables

**Fig. 3:**
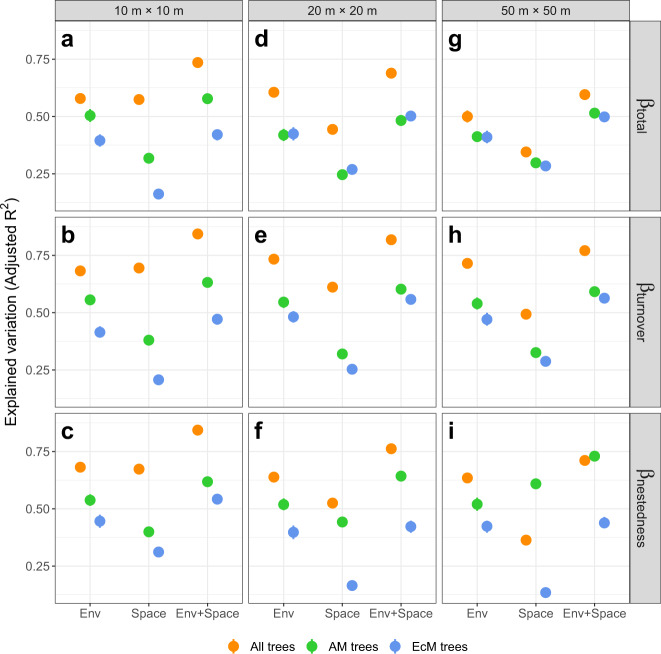
Variation partitioning of tree beta-diversity. Variation of total beta-diversity, species turnover, and species nestedness of all trees, AM trees, and EcM trees at quadrats scales of 10 m × 10 m (**a**–**c**), 20 m × 20 m (**d**–**f**), and 50 m × 50 m (**g**–**i**) explained by spatial and environmental variables. Orange, green, and blue points represent total beta-diversity and its two components (species turnover & nestedness) of all trees, AM trees, and EcM trees. “Env”, “Space”, and “Env + Space” represent the effects of environmental variables, spatial variables, and both, respectively. Average total beta-diversity and its two components were calculated based on 30, 15, and 15 randomly sampled quadrats of 10 m × 10 m, 20 m × 20 m, and 50 m × 50 m from each forest plot, respectively. The calculation and variation partitioning of total beta-diversity and its components were repeated 200 times. Means and 95% confidence intervals (95% CIs) of explained variation of total beta-diversity and its components were estimated using the non-parametric bootstrapping (*n* = 200 replicates). The means were showed as points and 95% CIs were showed as error bars. Differences between the variation explained by spatial and environmental variables were tested for significance using two-sided Mann–Whitney U tests: n.s. *P* ≥ 0.05, * *P* < 0.05, ** *P* < 0.01, *** *P* < 0.001. W = 19225 and *P* = 0.5029 for species turnover, W = 21765 and *P* = 0.127 for species nestedness, and W = 20557 and *P* = 0.6303 for total beta-diversity at the scale of 10 m × 10 m, and *P* < 0.0001 for others.

Environmental and spatial variables jointly generally explained at least 50% of the variation in total beta-diversity, species turnover, and species nestedness across latitudes for all trees and AM trees (Fig. 3). Generally, environmental and spatial variables jointly explained larger variations in total beta-diversity, species turnover, and species nestedness for AM trees and all trees than for EcM trees (Fig. 3). Variation explained by environmental variables was generally much larger than the variation explained by spatial variables for AM trees and EcM trees (Mann–Whitney U test: *P* < 0.0001; Fig. 3) except for species nestedness of AM trees at the scale of 50 m × 50 m (Mann–Whitney U test: *P* < 0.0001; Fig. 3). For all trees, compared to spatial variables, environmental variables explained equal variation at the scale of 10 m × 10 m (Mann–Whitney U test: W = 20557, *P* = 0.630 for total beta-diversity, W = 19225, *P* = 0.503 for species turnover, and W = 21765, *P* = 0.127 for species nestedness; Fig. 3), but much greater variation at the scales of 20 m × 20 m (Mann–Whitney U test: *P* < 0.0001; Fig. 3) and 50 m × 50 m (Mann–Whitney U test: *P* < 0.0001; Fig. 3) in total beta-diversity, species turnover, and species nestedness.

**Fig. 4:**
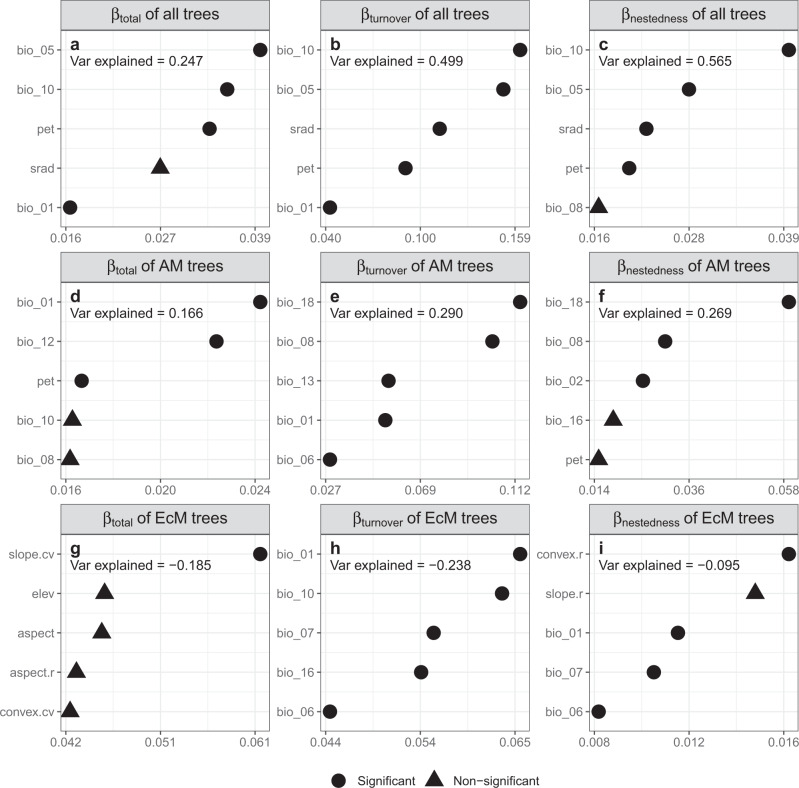
Specific effects of environmental variables on latitudinal gradients in tree beta-diversity. Relative importance of five most important environmental factors for total beta-diversity, species turnover, and species nestedness of all trees (**a**–**c**), AM trees (**d**–**f**), and EcM trees (**g**–**i**) at the scale of 20 m × 20 m. Total beta-diversity and its components are the mean values of 200 replicates of average pairwise beta-diversity and its component metrics calculated based on 15 randomly sampled quadrats of 20 m × 20 m from each forest plot. The relative importance of variables was ranked by the increase in node purity (horizontal axis). The proportion of variance displayed was explained by all of 34 environmental variables. Circle points indicate significant importance of predictors whereas triangles indicate non-significant importance of predictors. The meanings of environmental variables are as follows: bio_01 = Annual Mean Temperature, bio_02 = Mean Diurnal Range (Mean of monthly (max temp - min temp)), bio_05 = Max Temperature of Warmest Month, bio_06 = Min Temperature of Coldest Month, bio_07 = Temperature Annual Range (BIO5-BIO6), bio_08 = Mean Temperature of Wettest Quarter, bio_10 = Mean Temperature of Warmest Quarter, bio_12 = Annual Precipitation, bio_13 = Precipitation of Wettest Month, bio_16 = Precipitation of Wettest Quarter, bio_18 = Precipitation of Warmest Quarter, srad = Solar Radiation, pet = Potential Evapotranspiration, elev = Elevation, aspect = Slope Aspect, aspect.r = Range of Aspect, convex.r = Range of Curvature, slope.cv = Coefficient of Variation of Slope, convex.cv = Coefficient of Variation of Curvature.

Random forest analyses indicated that multiple environmental variables were generally not correlated with total beta-diversity, species turnover, or species nestedness of EcM trees, except for species nestedness of EcM trees at the scale of 10 m × 10 m (Fig. 4 and Supplementary Figs. 5, 6). In contrast, environmental variables jointly and differentially affected total beta-diversity, species turnover, and species nestedness of AM trees and all trees across quadrat scales. Specifically, mean, maximum, and minimum values of temperature and precipitation, solar radiation, aridity index, and potential evapotranspiration were positively associated with total beta-diversity and species turnover, but negatively with species nestedness of AM trees and all trees (Supplementary Fig. 7.1–7.6, 7.10–7.15, and 7.19–7.24). In contrast, temperature variability (i.e., mean diurnal range of temperature, isothermality, temperature seasonality, and temperature annual range), precipitation seasonality, and topographic variables were generally not correlated with total beta-diversity, species turnover, or species nestedness of trees (Fig. 4 and Supplementary Figs. 5–7). Total beta-diversity, species turnover, and species nestedness of all trees and AM trees were affected most by temperature and precipitation of the warmest quarter (Fig. 4 and Supplementary Figs. 5–7).

## Discussion

### AM trees influence the latitudinal gradient in tree beta-diversity

Despite widespread interest in patterns of forest beta-diversity across biogeographic gradients, the role of mutualistic biotic interactions in shaping these gradients remains largely unknown. Our findings based on 45 large forest plots covering a wide range of latitudes (25.1° S ~ 61.3° N) and on simulation experiments provided insights into the roles of mutualistic mycorrhizal associations, as well as climate, on patterns of beta-diversity. First, we found that latitudinal patterns of total beta-diversity, species turnover, and species nestedness are strongly associated with mutualistic associations among tree species and mycorrhizal fungi. Specifically, we found that community-wide patterns of total beta-diversity, species turnover, and species nestedness of all trees largely reflect those same patterns for AM trees (Fig. 2 and Supplementary Figs. 1–3). In contrast, for EcM trees, total beta-diversity and species turnover generally lacked significant latitudinal patterns, although patterns of species nestedness were mixed (Fig. 2 and Supplementary Figs. 1–3). This suggests that AM trees are the predominant contributors to overall latitudinal gradients in tree beta diversity. Second, for AM trees and all trees, total beta-diversity, species turnover, and species nestedness were generally largely explained by environmental factors, especially temperature and precipitation, suggesting the latitudinal gradients in beta-diversity may be largely driven by deterministic processes. In contrast, for EcM trees, beta-diversity, species turnover, and species nestedness were not strongly associated with environmental variables.

Mutualistic associations between mycorrhizal fungi and host plants may contribute to latitudinal gradients in beta-diversity observed in the present study via several mechanisms. First, mycorrhizal associations may affect beta-diversity by mediating the strength of interspecific competition and local distribution ranges of species through plant-soil feedbacks (PSFs), which are generally negative for AM trees and positive for EcM trees^26,34–36^. Negative PSFs for AM trees may enhance the recruitment of heterospecific trees, especially those rare tree species^26,39,41^. Replacement of conspecific individuals by heterospecific individuals represents the abundance-weighted species turnover. The maintenance of more rare species especially those with specific habitat preferences, may lead to higher species turnover among localities potentially due to habitat filtering^33^. Moreover, negative PSFs may restrict local distribution ranges of species, generating higher species turnover among localities due to fewer shared species with narrower distributions^34,36^. As negative PSFs may decrease with increasing latitude^44^, weaker negative PSFs for AM trees at higher latitudes could decrease species turnover^25,33,34,36^. Our result that species turnover of AM trees and all trees decreased with increasing latitude is in line with our expectation. In contrast, positive PSFs for EcM trees, which may be better protected against soil pathogens by fungal root mantle, may instead promote success of conspecific individuals, and may consequently lead to competitive exclusion of competitively inferior species^26,35^. In turn, selective species loss from competitive exclusion increases species nestedness^5,26,35^. Increasing prevalence of EcM-associations at higher latitudes should increase species nestedness of EcM trees with positive PSFs^5,25,26,35^. Our result that species nestedness of EcM trees generally increased with latitude, supports our expectation.

Second, mycorrhizal associations may affect beta-diversity by influencing speciation rates and extinction rates through habitat adaptation^23–25,30,31^. Mycorrhizal associations may promote habitat adaptation of host plants by increasing access to soil nutrients. However, the lack of comparable soil-nutrient data for most of forest plots in this study (data from online soil databases are too coarse at the forest plot scale as soil properties vary substantially across space even in a short distance) prevents us from explicitly testing the soil nutrient effects on the latitudinal beta-diversity gradients of AM and EcM trees. However, this will be a promising direction for the future studies. Global biogeography of AM trees was reported to be primarily driven by high litter decomposition rates whereas EcM trees was primarily driven by low litter decomposition rates^25^. Tropical habitats with higher decomposition rates may promote AM trees whereas temperate habitats with lower decomposition rates may promote EcM trees^25^. Adaptation to tropical regions may increase speciation rates and decrease extinction rates of AM trees, and may consequently lead to larger species pools of AM trees compared to those in temperate regions^24,25,31^. Larger species pools may increase beta-diversity by strengthening species uniqueness among localities, i.e., species turnover, due to narrow local distribution ranges of AM trees^34,36^. In addition, decreased extinction rates in tropical regions may consequently reduce the loss of species that creates species nestedness^5,24,25^. Our results that total beta-diversity and species turnover of AM trees generally decreased with increasing latitude while species nestedness of AM trees increased are in accordance with our expectation. Similarly, adaptation to temperate habitats may decrease extinction rates and increase speciation rates and species pools of EcM trees at temperate compared to tropical latitudes^30,31^. Larger species pools may in turn influence species turnover of EcM trees^15,32^. However, we found that species turnover of EcM trees was generally not correlated with latitude (Fig. 2 and Supplementary Figs. 1–3), which partly violated our expectation that species turnover of trees decreased from tropics towards poles. Two reasons, not mutually exclusive, may explain this unexpected result. First, the species pool of EcM trees was generally not (or extremely weakly) correlated with latitude (Supplementary Fig. 8); because positive PSFs may increase local distribution ranges and inhibit heterospecifics, this may counteract the effects of greater speciation rates on the species pool of EcM trees at temperate latitudes. Second, EcM trees with wide local distribution ranges may not significantly influence species turnover among localities^26,34,36^. We found that AM trees predominantly contributed to the latitudinal gradients in beta-diversity of all trees, supporting our first hypothesis. One potential reason may be the local distribution ranges mediated by PSFs. AM trees with narrow local distribution ranges may disproportionately contribute to the overall composition dissimilarity among localities (beta-diversity), whereas EcM trees with wide local distribution ranges may homogenize the overall species composition^26,34,36^. The simulation experiments confirmed that the strong contribution of AM trees to the latitudinal gradient is not simply a function of disparate abundance or species richness between AM and EcM trees (Supplementary Figs. 2 and 3). These observed and simulated results jointly suggested that processes strongly relevant to mycorrhizal associations, such as PSFs and habitat adaptation, rather than sampling bias (i.e., greater abundance or richness of AM trees) may contribute to the latitudinal gradients in beta-diversity of trees.

Although general latitudinal patterns of beta-diversity were detected, we also found three exceptional patterns at the scale of 50 m × 50 m: (1) total beta-diversity of AM trees was not correlated with latitude; (2) total beta-diversity of EcM trees decreased with increasing latitude; and (3) species nestedness of EcM trees was not correlated with latitude. These exceptions may result from the scale-dependent strength of PSFs, a possibility in need of further exploration. Distance between conspecific individuals in different quadrats may increase with quadrat size, and consequently PSFs of conspecific individuals among quadrats may decrease^45^. In turn, weaker positive PSFs may decrease species nestedness^5,26,35^. This may be more prevalent in temperate regions than in tropical regions because temperate regions have higher prevalence of EcM trees with positive PSFs compared to tropical regions^5,25,26,35,45^. Thus, species nestedness may decrease faster in temperate than in tropical regions, which may consequently shape a neutral trend in species nestedness of EcM trees at the scale of 50 m × 50 m. Similarly, weaker PSFs may also shape a decreasing trend of total beta-diversity for EcM trees and a neutral trend of total beta-diversity for AM trees via species turnover and species nestedness because total beta-diversity is the sum of species turnover and species nestedness.

### Effects of climatic factors on mycorrhizal-mediated tree beta-diversity, species turnover, and species nestedness

The result that environmental variables generally explained more variations in total beta-diversity, species turnover, and species nestedness than did spatial variables suggests a predominant role of habitat filtering in shaping the latitudinal patterns of total beta-diversity and its two components, supporting our second hypothesis. Climatic factors have been found to be extremely important for beta-diversity^3,32^. Temperature and precipitation, in particular, were significantly associated with total beta-diversity, species turnover, and species nestedness of AM trees and all trees (Figs. 3, 4 and Supplementary Figs. 5–7). In contrast, total beta-diversity, species turnover, and species nestedness of EcM trees were not correlated with climatic variables in most cases (Fig. 4 and Supplementary Figs. 5–7). Climatic variables can exert effects directly and indirectly through biotic interactions on the relatively local-scale diversity patterns^46^. We found that the latitudinal beta-diversity gradients and the effects of climatic factors on beta-diversity were highly dependent on the mycorrhizal types of trees, supporting our third hypothesis. These findings suggest that climate may likely affect latitudinal gradients in beta-diversity, species turnover, and species nestedness of host trees indirectly through its influence on mycorrhizal associations^24,25,34,36^, although climate may also directly affect processes shaping beta-diversity such as speciation, extinction, and dispersal limitation^5,7–11^.

Previous studies have shown that AM fungi are physiologically less tolerant than EcM fungi to low temperatures and decrease colonization below 15 °C, due to the lack of cold-tolerant traits^24,47,48^. In contrast, EcM fungi with cold-tolerant traits are well adapted to low temperatures^24,25^. In addition, temperature and precipitation are positively correlated with litter decomposition rate which has been reported to be the primary driver differentiating mycorrhizal associations between AM and EcM trees^24,25^. Thus, the prevalence of AM-associations was positively correlated with higher temperature and greater precipitation toward the equator, whereas the prevalence of EcM-associations was more common at low temperature and precipitation toward the poles (Supplementary Figs. 8–10)^24,25^. In turn, AM-associations may contribute to the decreasing trends in beta-diversity and species turnover and the increasing trends in species nestedness of host trees across latitudes (Fig. 2) via negative PSFs and habitat adaptation. In contrast, EcM-associations may contribute to the neutral latitudinal trends in beta-diversity and species turnover, but contribute to an increasing trend in species nestedness of host trees with latitude (Fig. 2), possibly through positive PSFs and habitat adaptation^26,34,36^.

In summary, we found that total beta-diversity and species turnover of both AM trees and all trees significantly decreased with increasing latitude, while species nestedness increased. Species nestedness of EcM trees also generally increased with latitude, whereas total beta-diversity and species turnover of EcM trees were generally not correlated with latitude, probably due to the wide local distributions of EcM trees which did not influence the overall compositional differences among localities. The latitudinal patterns of total beta-diversity, species turnover, and species nestedness of all trees were largely contributed by AM rather than EcM trees. Environmental factors were generally much more important than spatial factors in shaping latitudinal patterns of beta-diversity and its components of AM trees and all trees, suggesting that habitat filtering on mycorrhizal associations may be a major ecological process that determines the latitudinal beta-diversity gradient in trees. In particular, temperature and precipitation were the most important environmental factors. Environmental variables likely drive latitudinal gradients in total beta-diversity, species turnover, and species nestedness by affecting mycorrhizal associations of trees. The major contribution of AM trees in forests to the latitudinal gradient in beta-diversity of forest communities underscores the importance of AM trees for global biodiversity conservation. However, the causal relationships between mycorrhizal associations and tree beta-diversity need further exploration in following studies. Future research is also needed to discover the mycorrhizal associations of more tree species and the characterization of individual mycorrhizae species, their specificity for tree hosts and environmental adaptations.

## Methods

### Study sites, topographic and climatic data

Our study included 45 large, stem-mapped forest-dynamics plots mainly from the ForestGEO network (http://www.forestgeo.si.edu/; Fig. 1)^49^. Plots were established and censused using a standardized protocol^50^. Plot size ranges from 2.1 ha (Nanjenshan) to 60 ha (Jianfengling) and plot latitude ranges from 25.1°S (Ilha do Cardoso, Brazil) to 61.3°N (Scotty Creek, Canada), covering all continents with forests (i.e., Asia, Africa, Europe, South America, North America, and Oceania; Fig. 1 and Supplementary Table 1). In each plot, all free-standing woody stems with a diameter at breast (DBH) ≥ 1 cm were identified to species, tagged, measured, and mapped. The Uholka plot in Ukraine was an exception as woody stems were censused from a DBH ≥ 6 cm^51^. We focused only on trees and excluded plants of other growth forms (i.e., lianas, palms, and shrubs). In total, the 45 plots included 2,804,270 trees of 3840 tree species and 156 plant families.

As previous studies have shown that average pairwise beta-diversity using abundance data is relatively insensitive to sampling effort^52–54^, we used tree-species abundances (numbers of individuals per species) to calculate beta-diversity as Bray-Curtis dissimilarity. The total beta-diversity was partitioned into the abundance-weighted species turnover component (hereafter species turnover) and the abundance-weighted species nestedness component (hereafter species nestedness)^55^. Beta-diversity and its two component measures were calculated using the following equations, respectively.

Total beta-diversity:

1 $${\beta }_{total}=\frac{B+C}{2A+B+C}$$

Abundance-weighted turnover component:

2 $${\beta }_{turnover}=\frac{{\rm{min }}(B,C)}{A+{\rm{min}}(B,C)}$$

Abundance-weighted nestedness component:

3 $${\beta }_{nestedness}=\frac{|B-C|}{2A+B+C}\ast \frac{A}{A+{\rm{min }}(B,C)}$$

where *A* is the sum of the abundances of all species that occur in both of a pair of quadrats, while *B* and *C* are the sum of the abundances of the species that are unique to one or the other of the pair of quadrats^55^.

To account for the potential scale-dependence of beta-diversity patterns, plots were divided into 10 m × 10 m, 20 m × 20 m, and 50 m × 50 m quadrats^6,56^. At the scale of 50 m × 50 m, we excluded plots smaller than 8 ha (Cocoli, Sherman, Nanjenshan, Ngardok) to ensure adequate sample size and statistical power. At each of the three quadrat sizes in each forest plot, we calculated the average total beta-diversity, species turnover, and species nestedness across all quadrats. To facilitate comparisons with other studies, we further controlled sampling effort by randomly sampling 30 non-overlapping quadrats of 10 m × 10 m, 15 quadrats of 20 m × 20 m, and 15 quadrats of 50 m × 50 m in each plot^57,58^. This sampling procedure was repeated 200 times for each quadrat size and the results were averaged for each plot with the 95% confidence interval (95% CI) calculated using nonparametric bootstrap without assuming normality^58^.

All trees were assigned to one of three mycorrhizal types: AM trees; EcM trees; and other trees including ErM trees (Ericoid mycorrhizal trees), NM trees (non-mycorrhizal trees), and trees with two or more mycorrhizal types recorded in literature^59,60^. We first assigned mycorrhizal type at the genus level (97.76% of all species) and then the family level (2.24% of all species). As AM- and EcM-associations are the most common mycorrhizal types for trees^60^, we focused predominantly on AM and EcM trees in the present study. Beta-diversity and its component measures were calculated for all trees (a combination of AM trees, EcM trees, and other trees), AM trees, EcM trees, all trees excluding AM trees, and all trees excluding EcM trees.

We examined the influence of environmental variables on beta-diversity using topographic and climatic variables. Topography may affect beta-diversity through its effects on microclimate and the resulting mycorrhizal associations^24,61,62^. Topographic variables included the elevation, aspect, and slope of each quadrat for all three quadrat sizes based on the measured or interpolated elevation values of four corners of each quadrat. We also included convexity, which is based upon the elevation of a quadrat relative to the eight adjoining quadrats that surround the focal quadrat^46,63^. Aspect was sin-transformed so that ‘sunward’ facing slopes in both the northern and southern hemispheres were treated equivalently. To calculate the mean value of each topographic variable for each forest plot, we averaged the values across quadrats for each of the three quadrat sizes. To test the effect of topographic heterogeneity on beta-diversity and its components, we calculated the ranges and the coefficients of variation of each of four topographic variables.

Climatic variables included 19 bioclimatic variables and solar radiation for each forest plot from the WorldClim Database (http://worldclim.org/version2; accessed on 2019-9-24) and potential evapotranspiration and aridity index from the Global Aridity Index (Global-Aridity) and Global Potential Evapo-Transpiration (Global-PET) Geospatial Database (https://cgiarcsi.community/data/global-aridity-and-pet-database/; accessed on 2019-9-24) based on the resolution of 30 arc seconds at the equator. Climatic variables were extracted from the database using the R package ‘raster’^64^.

### Statistical analyses

We used a beta regression model, without random effects, to examine the relationships of beta-diversity and its components with latitude using a single value of beta-diversity or its component for each forest plot (i.e., where each forest plot represents a data point). A beta regression model is a generalized linear model with a beta distribution for proportion data within an open interval between 0 and 1^65,66^. We set the data points with values of 0 and 1 to be 0.005 and 0.995, respectively. We used the logit link function in the beta regression model. As forest plots in the present study substantially differ in elevation (from 2.5 to 3285.7 m above sea level), which could strongly affect temperature and consequently affect tree distribution, we adjusted the absolute latitude weighted by elevation to examine the relationships of beta-diversity and its components with latitude. Previous studies have demonstrated that a 100-m upward shift is thermally equivalent to a 100-km poleward shift^13,67^. In addition, one degree of latitude is equivalent to 111 km of geographic distance. Thus, we adjusted the absolute latitude using the following equation to obtain the adjusted latitude used in the analyses:

4 $${Adjusted}{latitude}={elevation}/111+{\rm{|}}{latitude}{\rm{|}}$$

To explore the contributions of AM and EcM trees to beta-diversity of all trees, we first tested latitudinal relationships for AM trees, EcM trees, and all trees. To demonstrate the robustness of analyses to the limitation of the ambiguous mycorrhizal associations, we also tested the patterns by running models where species with dual mycorrhizal statuses were first classified as one type and then as the other type. We found that results remained qualitatively unchanged (Fig. 2 and Supplementary Figs. 11–14). Thus, we conducted further analyses by classifying trees with dual mycorrhizal statuses as “other trees” and displayed patterns using other classification methods only in the supplementary material (Supplementary Figs. 11–14). However, the latitudinal patterns of AM trees may parallel the patterns of trees with other mycorrhizal types. Thus, the contributions of AM trees to the patterns of all trees may be obscured by trees with other mycorrhizal types. This may also be the case for EcM trees. Therefore, we further tested latitudinal relationships of all trees excluding AM trees, as well as of all trees excluding EcM trees. Latitudinal patterns of all trees excluding AM trees and all trees excluding EcM trees were highly consistent with the latitudinal patterns of EcM trees and AM trees, respectively (Fig. 2 and Supplementary Fig. 1), which suggested the patterns of all trees were mainly determined by AM and EcM trees and were not obscured by trees with other mycorrhizal types. Based on these findings, we focused only on three groups for further analyses: all trees, AM trees, and EcM trees.

We used a beta regression model to test the latitudinal patterns of the relative contributions of species turnover and species nestedness to the total beta-diversity. Differences between the relative contributions of species turnover and species nestedness were tested for significance using two-sided Mann–Whitney U tests.

Preliminary analyses detected decreasing individual density and species richness for all trees and AM trees with increasing latitude, but increasing individual density and no trend of species richness for EcM trees (Supplementary Figs. 8 and 9). To account for the possible effect of disproportionate abundance or species richness between AM and EcM trees on latitudinal beta-diversity gradients of all trees, we further conducted simulation experiments (Supplementary Methods).

To investigate potential mechanisms underlying latitudinal patterns of total beta-diversity, species turnover, and species nestedness, we used the variation partitioning analysis (VPA) based on the partial regression to separate the unique and shared effects of environmental and spatial variables. The pure effects of spatial variables suggest the importance of dispersal limitation or unmeasured environmental variables, whereas the pure effects of environmental variables point to the importance of habitat filtering (i.e., filtering out species unsuitable to specific habitat conditions)^58,63^. The fractions of variation in response variables explained by spatial and environmental variables were tested for significance by 999 permutations. We tested for the significance and visualized both the independent (i.e., variation explained by spatial or environmental variables) and joint effects (i.e., variation explained by both) of environmental and spatial variables because the shared effect of spatial and environmental variables cannot be tested for significance^68^. Environmental variables were selected using forward selection of the principal components of 22 climatic variables (19 bioclimatic variables, potential evapotranspiration, solar radiation, and aridity index), and 12 topographic variables including elevation, aspect, slope, convexity, and their ranges and coefficients of variation using the principal component analysis (PCA). Spatial variables were selected using forward selection of spatial eigenfunctions with positive values calculated with latitude and longitude using the Principal Components of Neighbor Matrices (PCNM). Latitude and longitude were the same for each of the three quadrat sizes.

Variation partitioning is an algorithm based on linear models which are sensitive to error distributions of response variables. Environmental and spatial variables used in variation partitioning were obtained from PCA and PCNM which were eigenvector analyses and likely to overestimate the explained variations even when the forward selection was applied^69^. Thus, variation partitioning in the present study was used mainly to qualitatively tease apart the relative importance of spatial and environmental variables.

The specific effects of environmental variables on beta-diversity were further explored using the random-forest modeling which is based on multiple bootstrapped regression trees^25,70^. Random forest is a robust algorithm which is insensitive to missing values, multicollinearity of explanatory variables, and error distributions of response variables for classification and regression^70^. Variable importance was determined by the increase in node purity which was measured as the decrease in the residual sum of squares if the variable was excluded^25^. As we had 34 environmental variables, we focused on the relative importance of the five most-important predictors for each response variable (i.e., beta-diversity, species turnover, and species nestedness of AM trees, EcM trees, and all trees) but provide partial-dependence plots for all environmental variables (Fig. 4 and Supplementary Figs. 5–7). Through the partial-dependence plots, we can visualize the direction of the effect of the explanatory variables^25^. Variable importance was tested for significance by 999 permutations. As the results of random forests were relatively consistent across quadrat sizes, here we only displayed the results of the five most-important predictors for each response variables at the quadrat size of 20 m × 20 m (See Supplementary Figs. 5–6 for the results of other scales).

All calculations and statistical analyses were performed on the R platform version 3.5.3^71^. Calculations of beta-diversity and its components were conducted using the “betapart” package^72^. Mann–Whitney U tests and PCA were implemented in the “stats” package. Variation partitioning analyses and PCNM were performed using the ‘vegan’ package^68^. Non-parametric bootstrap was conducted using the “Hmisc” package^73^. Beta regression models were performed using the “betareg” package^66^. Random forest analyses and partial-dependence plot visualization were implemented in the “randomForest” and “rfPermute” packages^74,75^.

### Reporting summary

Further information on research design is available in the Nature Research Reporting Summary linked to this article.

## Supplementary information

Supplementary Information

Reporting Summary

## Data Availability

Full raw census data are available on reasonable request from the ForestGEO (https://www.forestgeo.si.edu/). Bioclimatic variables and solar radiation are available from the WorldClim Database (http://worldclim.org/version2) and potential evapotranspiration and aridity index are available from the Global Aridity Index (Global-Aridity) and Global Potential Evapo-Transpiration (Global-PET) Geospatial Database (https://cgiarcsi.community/data/global-aridity-and-pet-database/).

## Code availability

Custom codes for simulations are available in the Supplementary Information files.
