# Supplementary material for: Arbuscular mycorrhizal trees influence the latitudinal beta-diversity gradient of tree communities in forests worldwide
Source: Nat Commun. 2021 May 25;12:3137. doi: 10.1038/s41467-021-23236-3 (PMC8149669; doi:10.1038/s41467-021-23236-3)
Supplement: Supplementary file 1 — Supplementary Information [file 41467_2021_23236_MOESM1_ESM.pdf]

Arbuscular mycorrhizal trees influence the latitudinal beta-diversity gradient of tree communities in forests worldwide

Yonglin Zhong[1], Chengjin Chu[1*], Jonathan A. Myers[2], Gregory S. Gilbert[3], James A. Lutz[4], Jonas Stillhard[5], Kai Zhu[3], Jill Thompson[6], Jennifer L. Baltzer[7], Fangliang He[8,9,10], Joseph A. LaManna[11], Stuart J. Davies[12], Kristina J. Aderson-Teixeira[12,13], David F.R.P. Burslem[14], Alfonso Alonso[15], Kuo-Jung Chao[16], Xugao Wang[17], Lianming Gao[18], David A. Orwig[19], Xue Yin[1], Xinghua Sui[1], Zhiyao Su[20], Iveren Abiem[21,22,23], Pulchérie Bissiengou[24], Norm Bourg[13], Nathalie Butt[25,26], Min Cao[27], Chia-Hao Chang-Yang[28], Wei-Chun Chao[29], Hazel Chapman[23], Yu-Yun Chen[30], David A. Coomes[31], Susan Cordell[32], Alexandre A. de Oliveira[33], Hu Du[34], Suqin Fang[1], Christian P. Giardina[32], Zhanqing Hao[35], Andrew Hector[36], Stephen P. Hubbell[37], David Janík[38], Patrick A. Jansen[12,39], Mingxi Jiang[40], Guangze Jin[41], David Kenfack[12,42], Kamil Král[38], Andrew J. Larson[43], Buhang Li[1], Xiankun Li[44], Yide Li[45], Juyu Lian[46], Luxiang Lin[27], Feng Liu[47], Yankun Liu[48], Yu Liu[9,10], Fuchen Luan[49], Yahuang Luo[18], Keping Ma[50], Yadvinder Malhi[51], Sean M. McMahon[12,52], William McShea[13], Hervé Memiaghe[24], Xiangcheng Mi[50], Mike Morecroft[53], Vojtech Novotny[54], Michael J. O'Brien[55], Jan den Ouden[56], Geoffrey G. Parker[57], Xiujuan Qiao[40], Haibao Ren[50], Glen Reynolds[58], Pavel Samonil[38], Weiguo Sang[59], Guochun Shen[10], Zhiqiang Shen[1], Guo-Zhang Michael Song[60], I-Fang Sun[30], Hui Tang[1], Songyan Tian[48], Amanda L. Uowolo[32], María Uriarte[61], Bin Wang[44], Xihua Wang[10], Youshi Wang[1], George D. Weiblen[62], Zhihong Wu[49], Nianxun Xi[1], Wusheng Xiang[44], Han Xu[45], Kun Xu[63], Wanhui Ye[46], Mingjian Yu[64], Fuping Zeng[34], Minhua Zhang[9,10], Yingming Zhang[49], Li Zhu[50], Jess K. Zimmerman[65]

[1]Department of Ecology, State Key Laboratory of Biocontrol and School of Life Sciences, Sun Yat-sen University, 510275 Guangzhou
[2]Department of Biology, Washington University in St. Louis, St. Louis, MO, USA
[3]Department of Environmental Studies, University of California, Santa Cruz, CA 95064, USA
[4]Wildland Resources Department, Utah State University, Logan, UT, USA
[5]Swiss Federal Research Institute for Forest, Snow and Landscape Research WSL, Forest Resources and Management, Zürcherstrasse 111, 8903 Birmensdorf, Switzerland
[6]UK Centre for Ecology & Hydrology Bush Estate, Penicuik, Midlothian EH26 0QB, UK
[7]Biology Department, Wilfrid Laurier University, Waterloo, ON, Canada
[8]Department of Renewable Resources, University of Alberta, Edmonton, Alberta, T6G 2H1, Canada
[9]ECNU-Alberta Joint Lab for Biodiversity Study, Tiantong National Station for Forest Ecosystem Research, East China Normal University, 200241 Shanghai

[10]Zhejiang Tiantong Forest Ecosystem National Observation and Research Station, School of Ecology and Environmental Sciences, East China Normal University, 200241 Shanghai
[11]Department of Biological Sciences, Marquette University, Milwaukee, WI 53201-1881, USA
[12]Forest Global Earth Observatory, Smithsonian Tropical Research Institute, Washington, DC, USA
[13]Conservation Ecology Center, Smithsonian Conservation Biology Institute, National Zoological Park, Front Royal, VA, USA
[14]School of Biological Sciences, University of Aberdeen, Aberdeen, UK
[15]Center for Conservation and Sustainability, Smithsonian Conservation Biology Institute, National Zoological Park, Washington, DC, USA
[16]International Master Program of Agriculture, National Chung Hsing University, 40227 Taichung
[17]CAS Key Laboratory of Forest Ecology and Management, Institute of Applied Ecology, Chinese Academy of Sciences, 110016 Shenyang
[18]CAS Key Laboratory for Plant Diversity and Biogeography of East Asia, Kunming Institute of Botany, Chinese Academy of Sciences, 650201 Kunming
[19]Harvard Forest, Harvard University, Petersham, MA, USA
[20]College of Forestry and Landscape Architecture, South China Agricultural University, 510642 Guangzhou
[21]Department of Plant Science and Technology, University of Jos, Jos, Nigeria
[22]The Nigerian Montane Forest Project, Taraba State, Nigeria
[23]School of Biological Sciences, University of Canterbury, Christchurch, New Zealand
[24]Institut de Recherche en Ecologie Tropicale/Centre National de la Recherche Scientifique et Technologique, Libreville, Gabon
[25]School of Biological Sciences, The University of Queensland, St. Lucia, QLD, Australia
[26]Centre for Biodiversity and Conservation Science, The University of Queensland, St. Lucia, QLD, Australia
[27]CAS Key Laboratory of Tropical Forest Ecology, Xishuangbanna Tropical Botanical Garden, Chinese Academy of Sciences, 650201 Kunming
[28]Department of Biological Sciences, National Sun Yat-sen University, 80424 Kaohsiung
[29]Department of Forestry and Natural Resources, National Chiayi University, 60004 Chiayi
[30]Department of Natural Resources and Environmental Studies, National Dong Hwa University, 97401 Hualien
[31]Department of Plant Sciences, University of Cambridge, Cambridge CB2 3EA, UK
[32]Institute of Pacific Islands Forestry, Pacific Southwest Research Station, USDA Forest Service, 60 Nowelo Street, Hilo, Hawaii 96720-6805, USA
[33]Departamento Ecologia, Universidade de São Paulo, Instituto de Biociências, Cidade Universitária, São Paulo, SP, Brazil

[34]Key Laboratory of Agro-ecological Processes in Subtropical Region, Institute of Subtropical Agriculture, Chinese Academy of Sciences, 410125 Changsha
[35]School of Ecology and Environment, Northernwest Polytechnical University, 710072 Xi'an
[36]Department of Plant Sciences, University of Oxford, Oxford, UK
[37]Department of Ecology and Evolutionary Biology, University of California, Los Angeles, Los Angeles, CA, USA
[38]Department of Forest Ecology, Silva Tarouca Research Institute, Brno, Czech Republic
[39]Wildlife Ecology and Conservation Group, Wageningen University and Research, PO Box 47, 6700 AA Wageningen, The Netherlands
[40]Key Laboratory of Aquatic Botany and Watershed Ecology, Wuhan Botanical Garden, Chinese Academy of Sciences, 430074 Wuhan
[41]Center for Ecological Research, Northeast Forestry University, 150040 Harbin
[42]Department of Botany, National Museum of Natural History, Washington, DC, USA
[43]Wilderness Institute and Department of Forest Management, University of Montana, Missoula, MT, 59812, USA
[44]Guangxi Key Laboratory of Plant Conservation and Restoration Ecology in Karst Terrain, Guangxi Institute of Botany, Guangxi Zhuang Autonomous Region and Chinese Academy of Sciences, 541006 Guilin
[45]Research Institute of Tropical Forestry, Chinese Academy of Forestry, 510520 Guangzhou
[46]Key Laboratory of Vegetation Restoration and Management of Degraded Ecosystems, South China Botanical Garden, Chinese Academy of Sciences, 510650 Guangzhou
[47]The Administrative Bureau of Naban River Watershed National Nature Reserve, No.19 Yuanlin Road, 666100 Jinghong
[48]Heilongjiang Key Laboratory of Forest Ecology and Forestry Ecological Engineering, Heilongjiang Forestry Engineering and Environment Institute, 150040 Harbin
[49]Guangdong Chebaling National Nature Reserve, 512500 Shaoguan
[50]State Key Laboratory of Vegetation and Environmental Change, Institute of Botany, Chinese Academy of Sciences, 100093 Beijing
[51]Environmental Change Institute, School of Geography and the Environment, University of Oxford, Oxford OX1 3QY, UK
[52]Smithsonian Environmental Research Center, Edgewater, Maryland, USA
[53]Natural England, UK
[54]Biology Center of the Czech Academy of Sciences, Institute of Entomology and the University of South Bohemia, Branisovska 31, 37005 Ceske Budejovicve, Czech Republic
[55]Área de Biodiversidad y Conservación, Universidad Rey Juan Carlos, Móstoles, Madrid, Spain
[56]Forest Ecology and Management Group, Wageningen University, P.O. box 47, Wageningen, The Netherlands

[57]Forest Ecology Group, Smithsonian Environmental Research Center, Edgewater, MD, USA
[58]Southeast Asia Rainforest Research Partnership, Danum Valley Field Centre, PO Box 60282, 91112, Lahad Datu, Sabah, Malaysia
[59]College of Life and Environmental Science, Minzu University of China, 100081 Beijing
[60]Department of Soil and Water Conservation, National Chung Hsing University, 40227 Taichung
[61]Department of Ecology, Evolution and Environmental Biology, Columbia University, New York, NY 10027, USA
[62]Department of Plant & Microbial Biology, University of Minnesota, St. Paul, MN, USA
[63]Yunnan Lijiang Forest Ecosystem National Observation and Research Station, Kunming Instituted of Botany, Chinese Academy of Sciences, 674100 Lijiang
[64]MOE Key Laboratory of Biosystems Homeostasis & Protection, College of Life Sciences, Zhejiang University, 310058 Hangzhou
[65]Department of Environmental Sciences, University of Puerto Rico, San Juan, Puerto Rico 00925, USA

Corresponding author: Chengjin Chu, chuchjin@mail.sysu.edu.cn

Supporting information

Supplementary Methods

S1 Simulation experiments

S1.1 Controlling for abundance

For each forest plot, for example at the scale of 20 m × 20 m, we randomly selected 15 non-overlapping quadrats from the plot for the analyses of beta-diversity (we termed these 15 quadrats as the observed community). At the same time, we constructed a simulated community (similarly, consisting of 15 quadrats). The general steps of simulations are as follows:

1) Defining the size of the simulated community. First, we set the total number of individuals (i.e., the simulated community size) in the simulated communities to be the same as the total number of individuals in the observed communities along the adjusted latitude gradient (See Supplementary Fig. 9).

2) Constructing the simulated communities. To remove the possible effect of abundance of AM trees on the beta-diversity of all trees, we set the number of AM trees equal to the number of EcM trees in the simulated communities; the sum of the number of AM and EcM trees equals the community size described above. For AM trees, we sampled individuals with replacement from the species list of all AM trees in the forest plot. The species was selected based on the probability equal to the relative abundance of this species in the forest plot. We did the same selection process for the EcM trees.

3) Maintaining the spatial structure of individuals. To hold the spatial aggregation of species i in the simulated community as similar as possible to the observed community, we set the frequency of each species in the simulated community (Fi_sim) according to the following equation: Fi_obs / Nplot_obs = Fi_sim / Nplot_sim. Here, Nplot_obs is the total number of quadrats in the forest plot (not the observed community but the forest plot). Fi_obs is the occurrence frequency of species i (i.e., the number of quadrats in which species i was found in the forest plot) across the

quadrats consisting of the forest plot. For example, for the BCI 50-ha plot, at the scale of 20 m × 20 m, we have 1250 quadrats. Then Fi_obs is the occurrence frequency of species i in these 1250 quadrats and Nplot_obs is 1250 quadrats. Accordingly, Nplot_sim is the total number of quadrats in the simulated community, which is determined by the Step 2 above. Fi_sim is the occurrence frequency of species i in the simulated community across the quadrats (for example, 15 quadrats). When Fi_sim is known (estimated from the above equation), we can obtain the number of quadrats in which species i should occur. The abundance of species i, Ni_sim in the simulated community was sampled from the forest plot with the relative abundance of species i as sampling probability. Then we randomly assign Ni_sim individuals of species i to Fi_sim quadrats with one quadrat at least having one individual.

4) Selecting forest plots. To ensure adequate numbers of species of both AM and EcM trees, we only used forest plots with at least four AM tree species and four EcM tree species for the 10 m × 10 m quadrat size, at least nine AM tree species and nine EcM tree species for the 20 m × 20 m quadrat size, and at least nine AM tree species and nine EcM tree species for the 50 m × 50 m quadrat size. Ultimately, 31 out of 45 forest plots were retained for the 10 m × 10 m quadrat size, 21 forest plots for the 20 m × 20 m quadrat size, and 21 forest plots for the 50 m × 50 m quadrat size.

5) Calculating the metrics. When the simulated community is ready (consisting of a given number of quadrats), we calculated the metrics for beta-diversity as demonstrated in the main text.

Simulations were repeated 200 times and results were averaged. Simulations were conducted for 10 m × 10 m, 20 m × 20 m, and 50 m × 50 m quadrat sizes, respectively.

S1.2 Controlling for species richness

Simulations controlling for species richness were similar to the steps described above, with two exceptions. First, we set equal numbers of species rather than equal numbers

of individuals for AM and EcM trees in the simulated communities described in the Step 2 above. Second, all simulated communities at a given scale contained the same total number of species.

S1.3 R codes for simulations

1) Controlling for abundance

```
library(dplyr);library(reshape2);library(stringr);library(tibble);library(betapart)
### take a simulated dataset as an example at the scale of 10 m × 10 m
df1<-read.csv("df.test.csv", stringsAsFactors=F)[ ,c("latin.short","mycorr","plot")]
n.plot<-30 # number of plots of the observed/simulated communities
n.tree<-1000 # number of trees of the observed/simulated communities
bet.const<-data.frame(matrix(nrow = 200,ncol = 9))
Nplot<-n_distinct(df1$plot)
df<-group_by(df1,mycorr,latin.short)%>%summarise(n=n(),fr=n_distinct(plot))%>%
ungroup()%>%mutate(fr=fr/Nplot,rN=n/sum(n))
df$mycorr[df$mycorr!="AM"&df$mycorr!="EcM"]<-"others"
rm(df1)
  if(sum(unique(df$mycorr)%in%c("AM","EcM"))==2){
    for(j in 1:200){
      df.am<-sample(df$latin.short[df$mycorr=="AM"],size=n.tree/2,
prob=df$rN[df$mycorr=="AM"],replace = TRUE)
      df.em<-sample(df$latin.short[df$mycorr=="EcM"], size=n.tree/2, prob =
df$rN[df$mycorr=="EcM"],replace = TRUE)
      dft<-data.frame(table(c(df.am,df.em)));names(dft)[1]<-"latin.short"
      dft<-left_join(dft,distinct(df[,c("latin.short","n","fr","rN","mycorr")]))
      dft$np<-ceiling(n.plot*dft$fr)
      dft$np[dft$np>n.plot]<-n.plot
      df.mat<-list()
      for(k in seq_len(nrow(dft))){
```

```
        if(dft$Freq[k]>=dft$np[k]){
          pp1<-sample(paste("P",str_pad(1:n.plot,width = 2,side = "left", pad=0), 
sep = "") ,size = dft$np[k])
df.mat[[k]]<-data.frame(latin.short=rep(dft$latin.short[k],time=dft$Freq[k]), 
plot=c(pp1, sample(pp1,size = dft$Freq[k]-dft$np[k], 
replace=TRUE))%>%as.character())
        }else{
          pp1<-sample(paste("P",str_pad(1:n.plot,width = 2,side = "left", pad =0), 
sep = ""), size = dft$Freq[k])
df.mat[[k]]<-data.frame(latin.short=rep(dft$latin.short[k],time=dft$Freq[k]), 
plot=pp1)
        }
      }
      df.mat<-Reduce(rbind.data.frame,df.mat)
      df.new<-left_join(df.mat,df[,c("latin.short","mycorr")])
sp.mat<-dcast(df.new,plot~latin.short,length)%>%column_to_rownames("plot")%>%
as.matrix()
      bet<-beta.pair.abund(sp.mat)%>%lapply(as.vector)%>%
as.data.frame()%>%as.matrix()
      if(length(dim(bet))<2){
        bet.p<-rep(NA,3)
      }else{bet.p<-bet%>%colMeans(na.rm=TRUE)}
      ## AM
      df.am<-subset(df.new,mycorr=="AM")
      sp.mat.am<-dcast(df.am,plot~latin.short,length)%>%
column_to_rownames("plot")%>%as.matrix()
      bet.am<-beta.pair.abund(sp.mat.am)%>%lapply(as.vector)%>%
as.data.frame()%>%as.matrix()
      if(length(dim(bet.am))<2){
        bet.am.p<-rep(NA,3)
```

```
        }else{bet.am.p<-bet.am%>%colMeans(na.rm=TRUE)}
        ## EcM
        df.em<-subset(df.new,mycorr=="EcM")
        sp.mat.em<-dcast(df.em,plot~latin.short,length)%>%
column_to_rownames("plot")%>%as.matrix()
        bet.em<-beta.pair.abund(sp.mat.em)%>%lapply(as.vector)%>%
as.data.frame()%>%as.matrix()
        if(length(dim(bet.em))<2){
          bet.em.p<-rep(NA,3)
        }else{bet.em.p<-bet.em%>%colMeans(na.rm=TRUE)}
        bet.const[j,1:9]<-c(bet.p,bet.am.p,bet.em.p)
      }
  }
  names(bet.const)<-c("bal","gra","tot","bal.am","gra.am","tot.am",
"bal.em","gra.em","tot.em")
  dat<-summarise_all(bet.const,mean,na.rm=TRUE)
```

2) Controlling for species richness

```
library(dplyr);library(reshape2);library(stringr);library(tibble);library(betapart)
### take a simulated dataset as an example at the scale of 10 m × 10 m
df1<-read.csv("df.test.csv", stringsAsFactors=F)[ ,c("latin.short","mycorr","plot")]
n.plot<-30 # number of plots of the observed/simulated communities
n.tree<-1000 # number of individuals of the observed/simulated communities
s.tree<-4 #number of species of the simulated communities
bet.const<-data.frame(matrix(nrow = 200,ncol = 9))
  Nplot<-n_distinct(df1$plot)
  df<-group_by(df1,mycorr,latin.short)%>%
    summarise(n=n(),fr=n_distinct(plot))%>%ungroup()%>%
mutate(fr=fr/Nplot,rN=n/sum(n))
```

```
    df$mycorr[df$mycorr!="AM"&df$mycorr!="EcM"]<-"others"
    rm(df1)
    if(sum(unique(df$mycorr)%in%c("AM","EcM"))==2){
      for(j in 1:200){
        dfs<-group_by(df[df$mycorr=="AM"|df$mycorr=="EcM",],mycorr)%>%
          sample_n(size = s.tree)
        dff1<-dfs$latin.short
        dff2<-sample(dfs$latin.short, size = n.tree-length(dff1), prob = dfs$rN,replace
= TRUE)
        dft<-c(dff1,dff2)%>%table()%>%as.data.frame();names(dft)[1]<-"latin.short"
        dft<-left_join(dft,distinct(df[,c("latin.short","n","fr","rN","mycorr")]))
        dft$np<-ceiling(n.plot*dft$fr)
        dft$np[dft$np>n.plot]<-n.plot
        df.mat<-list()
        for(k in seq_len(nrow(dft))){
          if(dft$Freq[k]>=dft$np[k]){
            pp1<-sample(paste("P",str_pad(1:n.plot,width = 2,side = "left",pad
=0),sep = ""),size = dft$np[k])
df.mat[[k]]<-data.frame(latin.short=rep(dft$latin.short[k],time=dft$Freq[k]),
plot=c(pp1, sample(pp1,size = dft$Freq[k]-dft$np[k],replace =
TRUE))%>%as.character())
          }else{
            pp1<-sample(paste("P",str_pad(1:n.plot,width = 2, side = "left", pad=0),
sep = ""), size = dft$Freq[k])
df.mat[[k]]<-data.frame(latin.short=rep(dft$latin.short[k],time=dft$Freq[k]),
plot=pp1)
          }
        }
        df.mat<-Reduce(rbind.data.frame,df.mat)
        df.new<-left_join(df.mat,df[,c("latin.short","mycorr")])
```

```
sp.mat<-dcast(df.new,plot~latin.short,length)%>%column_to_rownames("plot")%>%
as.matrix()
        bet<-beta.pair.abund(sp.mat)%>%lapply(as.vector)%>%
as.data.frame()%>%as.matrix()
        if(length(dim(bet))<2){
          bet.p<-rep(NA,3)
        }else{bet.p<-bet%>%colMeans(na.rm=TRUE)}
        ## AM
        df.am<-subset(df.new,mycorr=="AM")
        sp.mat.am<-dcast(df.am,plot~latin.short,length)%>%
column_to_rownames("plot")%>%as.matrix()
        bet.am<-beta.pair.abund(sp.mat.am)%>%lapply(as.vector)%>%
as.data.frame()%>%as.matrix()
        if(length(dim(bet.am))<2){
          bet.am.p<-rep(NA,3)
        }else{bet.am.p<-bet.am%>%colMeans(na.rm=TRUE)}
        ## EcM
        df.em<-subset(df.new,mycorr=="EcM")
        sp.mat.em<-dcast(df.em,plot~latin.short,length)%>%
column_to_rownames("plot")%>%as.matrix()
        bet.em<-beta.pair.abund(sp.mat.em)%>%lapply(as.vector)%>%
as.data.frame()%>%as.matrix()
        if(length(dim(bet.em))<2){
          bet.em.p<-rep(NA,3)
        }else{bet.em.p<-bet.em%>%colMeans(na.rm=TRUE)}
        bet.const[j,1:9]<-c(bet.p,bet.am.p,bet.em.p)
      }
  }
names(bet.const)<-c("bal","gra","tot","bal.am","gra.am","tot.am","bal.em","gra.em","
tot.em")
```

```
dat<-summarise_all(bet.const,mean,na.rm=TRUE)
```

S1.4 Instruction to run the R codes

Simulations were performed on the R software version 3.5.3 on a computer with a two-core i5 CPU and with an operating system of Windows 10.

Type the following commands in R for package installation:
install.packages(c("reshape2","stringr","dplyr","tibble","betapart")).

Typical install time of the R software and the packages on a "normal" desktop was generally relatively short, but highly dependent on the internet used.

Run the simulation codes in Supplementary Methods 1.3 on the example data.

AM and EcM trees contributed relatively equally to the simulated beta-diversity and its components, because the example data were randomly generated without preserving the spatial distributions of AM and EcM trees, which meant ecological equivalence between AM and EcM trees in the example data.

The expected run time for demo was relatively short on a "normal" desktop.

Supplementary Figures

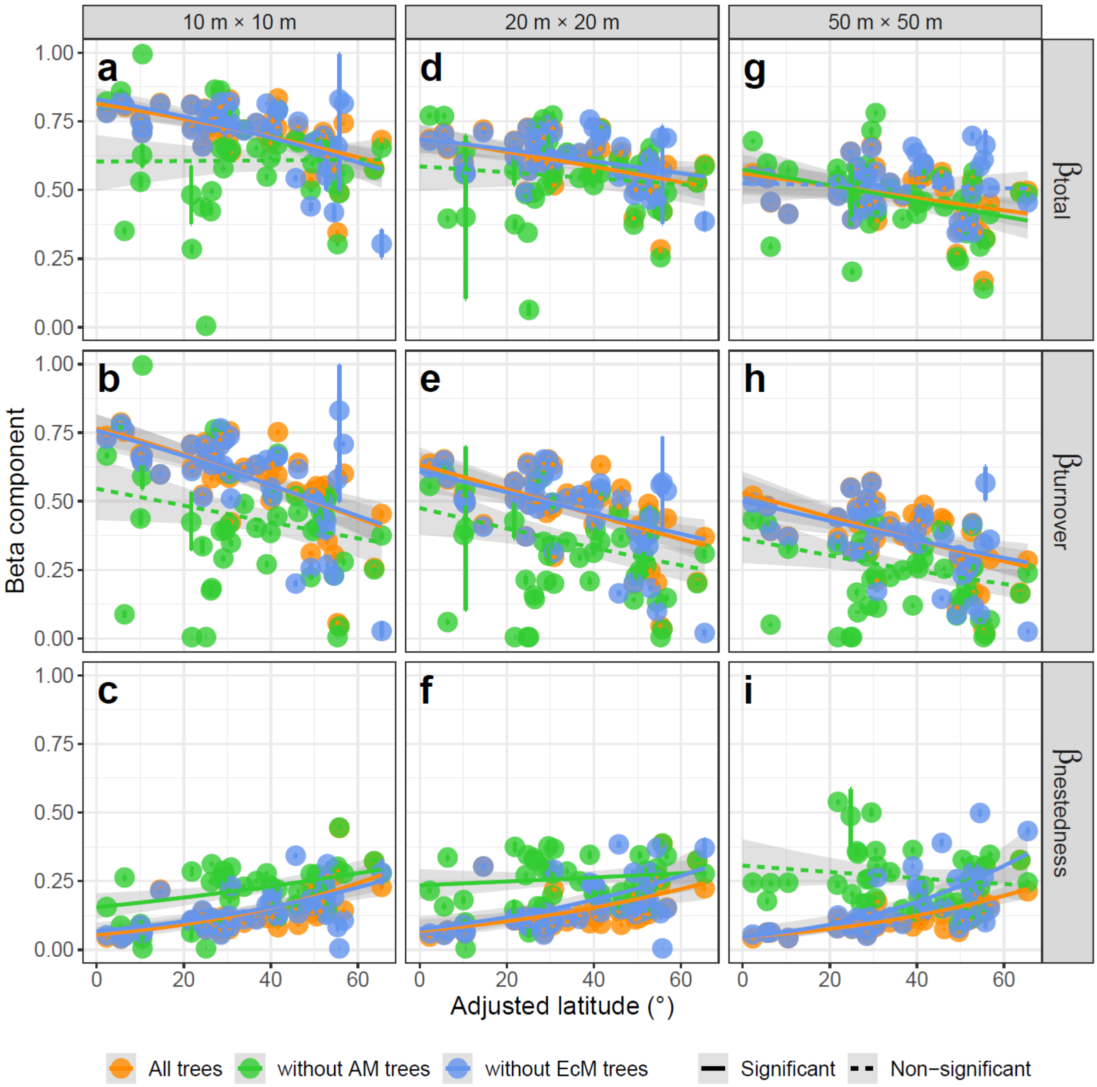

Figure S1 Total beta-diversity, species turnover, and species nestedness of all trees, trees excluding AM trees, and trees excluding EcM trees along latitude at the scale of 10 m × 10 m (a-c), 20 m × 20 m (d-f), and 50 m × 50 m (g-i). Orange points represent beta-diversity and its components of all trees and orange lines represent their latitudinal patterns. Green points represent beta-diversity and its components of trees excluding AM trees and green lines represent their latitudinal patterns. Blue points represent beta-diversity and its components of trees excluding EcM trees and blue lines represent their latitudinal patterns. Points are the mean values and the error bars are the 95% confidence intervals, estimated using non-parametric bootstrapping (n = 200). 200 replicates of average pairwise beta-diversity and its components were calculated based on 30, 15, and 15 randomly sampled quadrats of 10 m × 10 m, 20 m

× 20 m, and 50 m × 50 m from each forest plot, respectively. Solid lines indicate significant relationships with latitude whereas dashed lines indicate non-significant relationships fitted using the beta regression. The error bands (shaded areas) are the 95% confidence intervals of the fitted relationships, with sample size n = 45 for all trees, n = 43 for trees excluding AM trees, and n = 44 for trees excluding EcM trees at the 10 m × 10 m scale; with n = 45 for all trees, n = 44 for trees excluding AM trees, and n = 44 for trees excluding EcM trees at the 20 m × 20 m scale; and with n = 41 for all trees, n = 41 for trees excluding AM trees, and n = 40 for trees excluding EcM trees at the 50 m × 50 m scale.

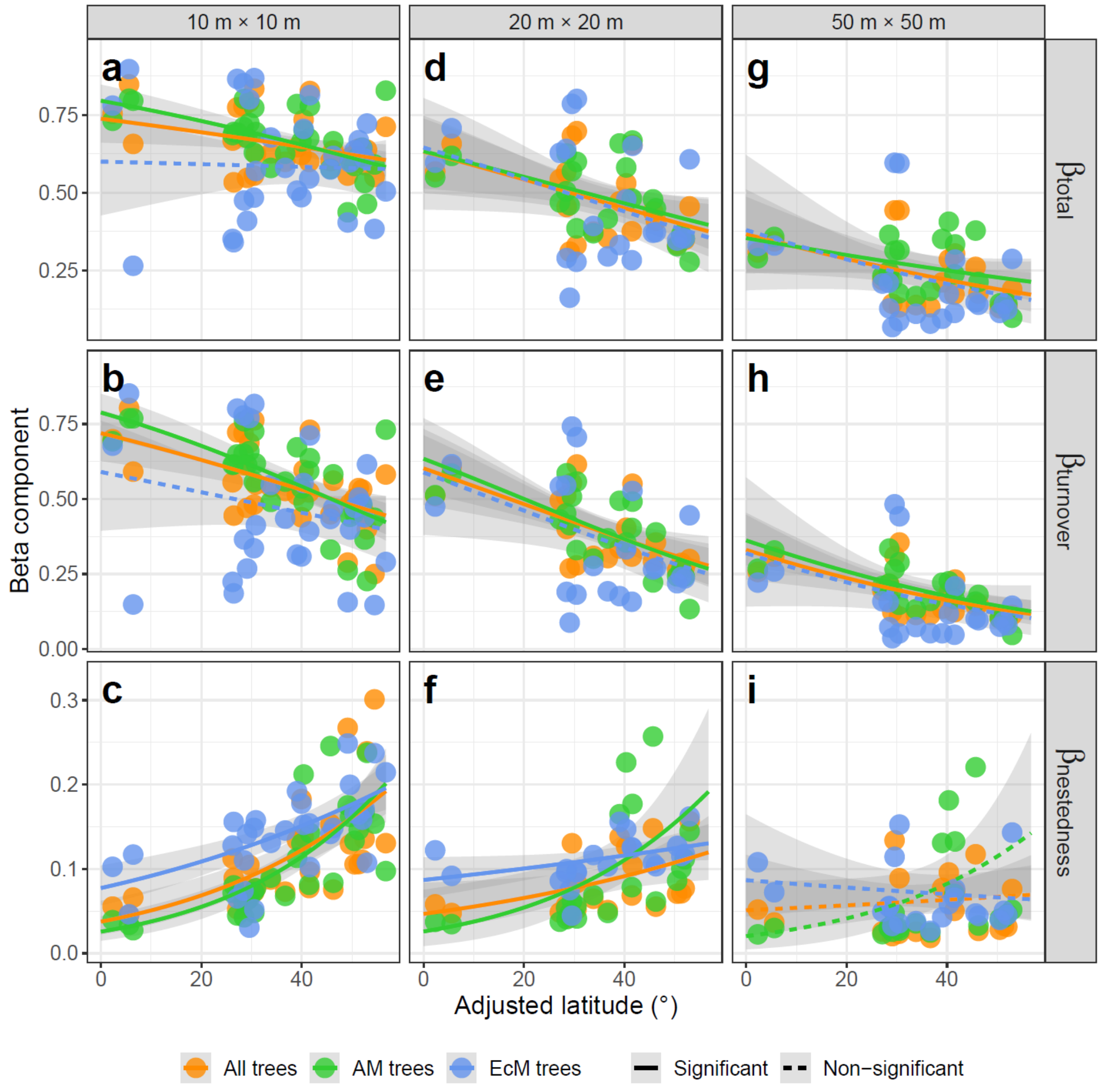

Figure S2 Total beta-diversity, species turnover, and species nestedness of all trees, AM trees, and EcM trees along latitude at the scale of 10 m × 10 m (a-c), 20 m × 20 m (d-f), and 50 m × 50 m (g-i) from the simulation experiment removing the effect of disproportionate abundance between AM and EcM trees. Orange points represent beta-diversity and its components of all trees and orange lines represent their latitudinal patterns. Green points represent beta-diversity and its components of AM trees and green lines represent their latitudinal patterns. Blue points represent beta-diversity and its components of EcM trees and blue lines represent their latitudinal patterns. Points are the mean values of 200 replicates of average pairwise beta-diversity and its components calculated based on 30, 15, and 15 simulated quadrats of 10 m × 10 m, 20 m × 20 m, and 50 m × 50 m from each forest plot, respectively. Solid lines indicate significant relationships with latitude whereas dashed lines indicate non-significant relationships fitted using the beta regression. The error bands (shaded areas) are the 95% confidence intervals of the fitted relationships, with sample size n = 31 at the 10 m × 10 m scale, n = 21 at the 20 m × 20 m scale, and n = 21 at the 50 m × 50 m scale.

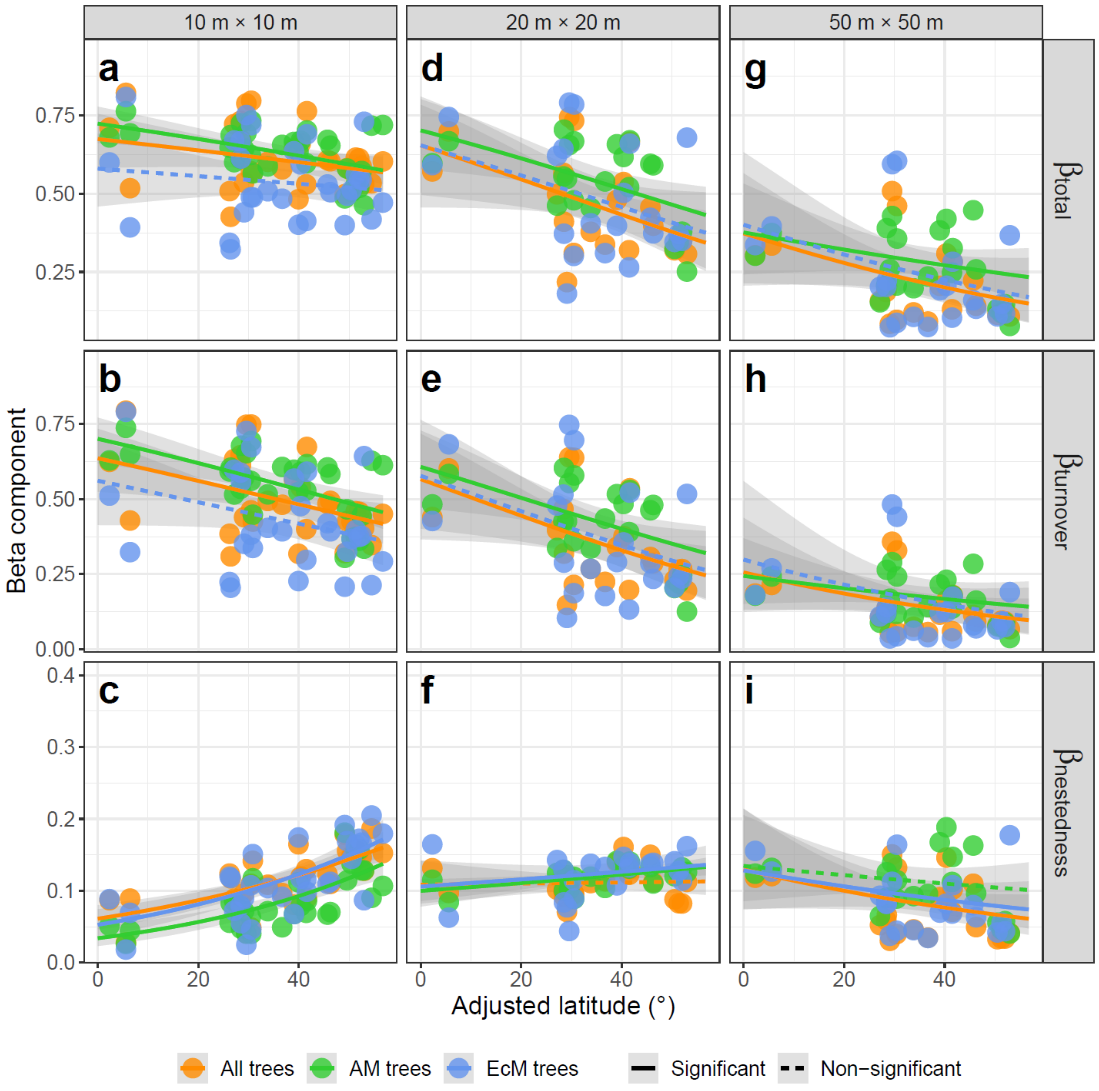

Figure S3 Total beta-diversity, species turnover, and species nestedness of all trees, AM trees, and EcM trees along latitude at the scale of 10 m × 10 m (a-c), 20 m × 20 m (d-f), and 50 m × 50 m (g-i) from the simulation experiment removing the effect of disproportionate species richness between AM and EcM trees. Orange points represent beta-diversity and its components of all trees and orange lines represent their latitudinal patterns. Green points represent beta-diversity and its components of AM trees and green lines represent their latitudinal patterns. Blue points represent beta-diversity and its components of EcM trees and blue lines represent their latitudinal patterns. Points are the mean values of 200 replicates of average pairwise beta-diversity and its components calculated based on 30, 15, and 15 simulated quadrats of 10 m × 10 m, 20 m × 20 m, and 50 m × 50 m from each forest plot, respectively. Solid lines indicate significant relationships with latitude whereas dashed

lines indicate non-significant relationships fitted using the beta regression. The error bands (shaded areas) are the 95% confidence intervals of the fitted relationships, with sample size n = 31 at the 10 m × 10 m scale, n = 21 at the 20 m × 20 m scale, and n = 21 at the 50 m × 50 m scale.

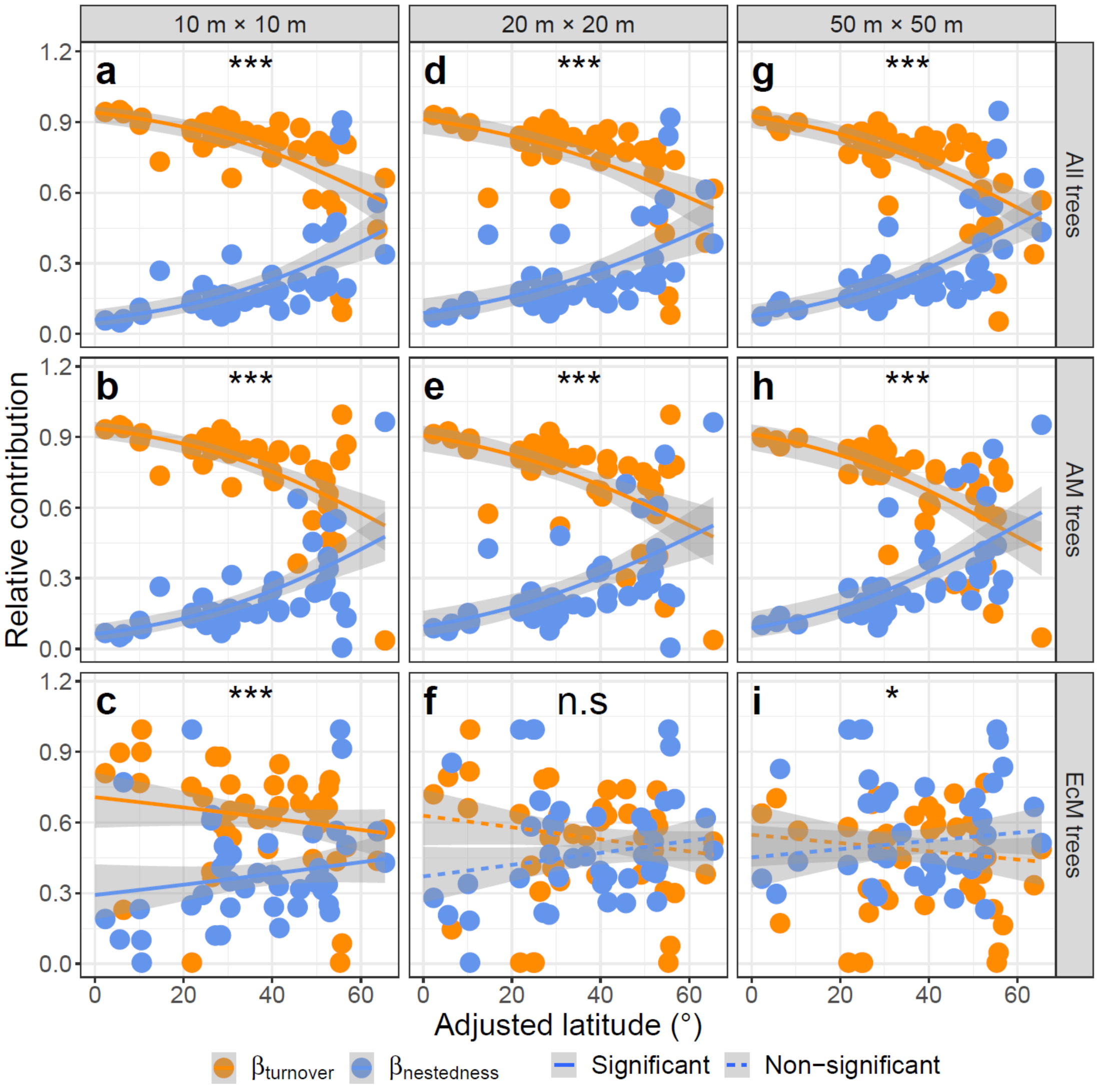

Figure S4 Relative contributions of species turnover and species nestedness to total beta-diversity of all trees, AM trees, and EcM trees across latitudes at quadrat scales of 10 × 10 m (a-c), 20 × 20 m (d-f), and 50 × 50 m (g-i). Orange points represent the relative contribution of species turnover component and orange lines represent their latitudinal patterns. Dark blue points represent the relative contribution of species nestedness and dark blue lines represent their latitudinal patterns. Relative contributions of species turnover and species nestedness to total beta-diversity were

calculated using the mean values of 200 replicates of average pairwise beta-diversity and its components calculated based on 30, 15, and 15 randomly sampled quadrats of 10 m × 10 m, 20 m × 20 m, and 50 m × 50 m from each forest plot, respectively. Solid lines indicate significant relationships whereas dashed lines indicate non-significant relationships fitted using the beta regression. The error bands (shaded areas) are the 95% confidence intervals of the fitted relationships. Results from two-sided Mann-Whitney U tests of differences between the relative contributions of species turnover and nestedness to total beta-diversity: n.s. $P \geq 0.05$, * $P < 0.05$, ** $P < 0.01$, *** $P < 0.001$. W = 1029 and $P = 0.6136$ for EcM trees at the scale of 20 m × 20, W = 582 and $P = 0.0167$ for EcM trees at the scale of 50 m × 50 m, and $P < 0.0001$ for others.

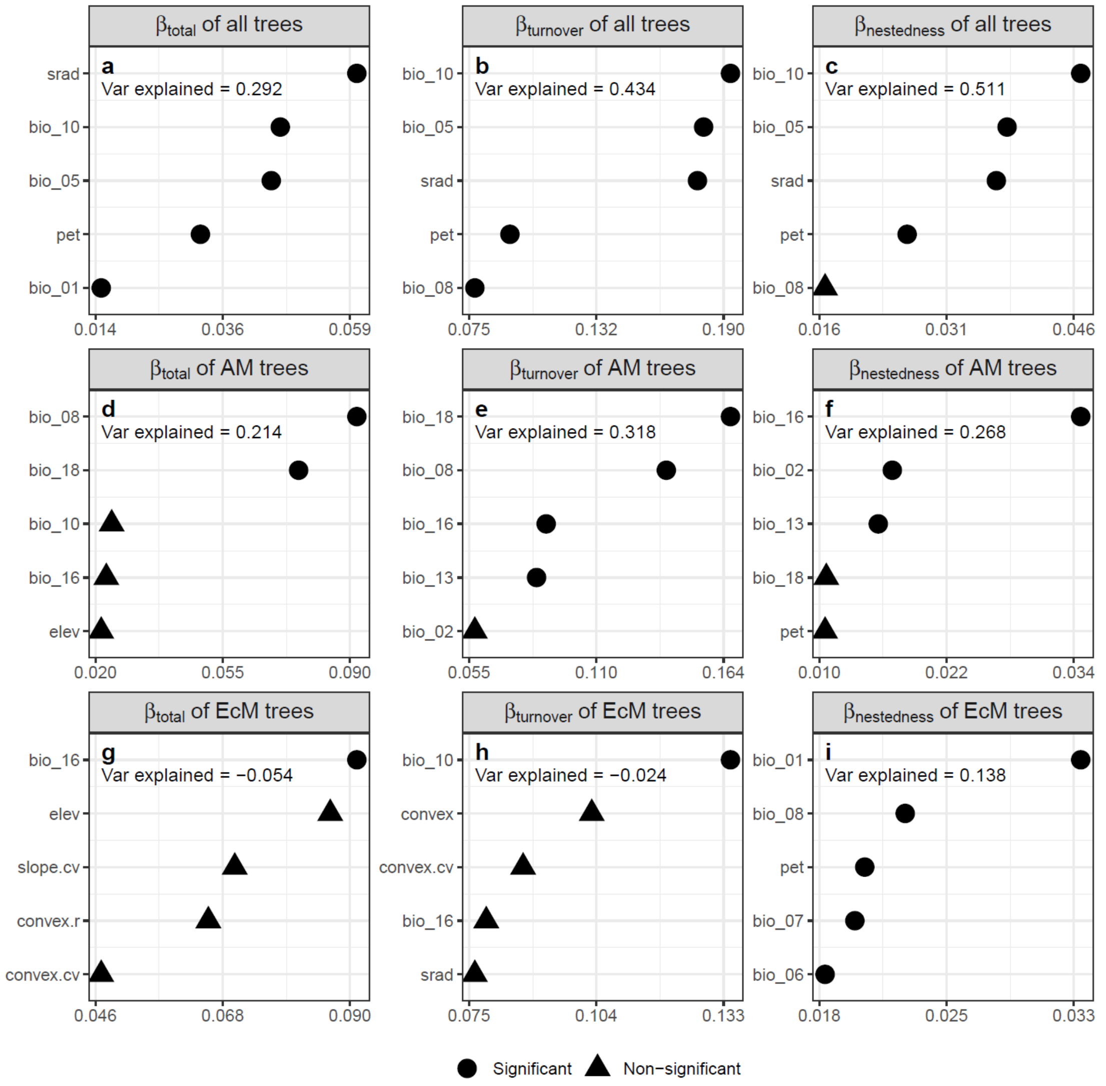

Figure S5 Relative importance of five most important environmental factors for total beta-diversity, species turnover, and species nestedness of all trees (a-c), AM trees (d-f), and EcM trees (g-i) at the scale of 10 m × 10 m. Total beta-diversity and its components are the mean values of 200 replicates of average pairwise beta-diversity and its component metrics calculated based on 30 randomly sampled quadrats of 10 m × 10 m from each forest plot. The relative importance of variables was ranked by the increase in node purity (horizontal axis). The proportion of variance displayed was that explained by all of 34 environmental variables. Circle points indicate significant importance of predictors whereas triangles indicate non-significant importance of predictors. The meanings of environmental variables are as follows: bio_01 = Annual Mean Temperature; bio_02 = Mean Diurnal Range (Mean of monthly (max temp - min temp)); bio_03 = Isothermality (BIO2/BIO7) (×100); bio_04 = Temperature

Seasonality (standard deviation ×100); bio_05 = Max Temperature of Warmest Month; bio_06 = Min Temperature of Coldest Month; bio_07 = Temperature Annual Range (BIO5-BIO6); bio_08 = Mean Temperature of Wettest Quarter; bio_09 = Mean Temperature of Driest Quarter; bio_10 = Mean Temperature of Warmest Quarter; bio_11 = Mean Temperature of Coldest Quarter; bio_12 = Annual Precipitation; bio_13 = Precipitation of Wettest Month; bio_14 = Precipitation of Driest Month; bio_15 = Precipitation Seasonality (Coefficient of Variation); bio_16 Precipitation of Wettest Quarter; bio_17 = Precipitation of Driest Quarter; bio_18 Precipitation of Warmest Quarter; bio_19 Precipitation of Coldest Quarter; srad = Solar radiation; AI = Aridity index; pet = Potential evapotranspiration; elev.r = range of elevation; aspect.r = range of aspect; slope.r = range of slope; convex.r = range of curvature; elev.cv = coefficient of variation of elevation; aspect.ccv = coefficient of variation of aspect; slope.cv = coefficient of variation of slope; convex.cv = coefficient of variation of curvature. The meanings of variable abbreviations in Supplementary Figs. 6- 7.27 are the same given here.

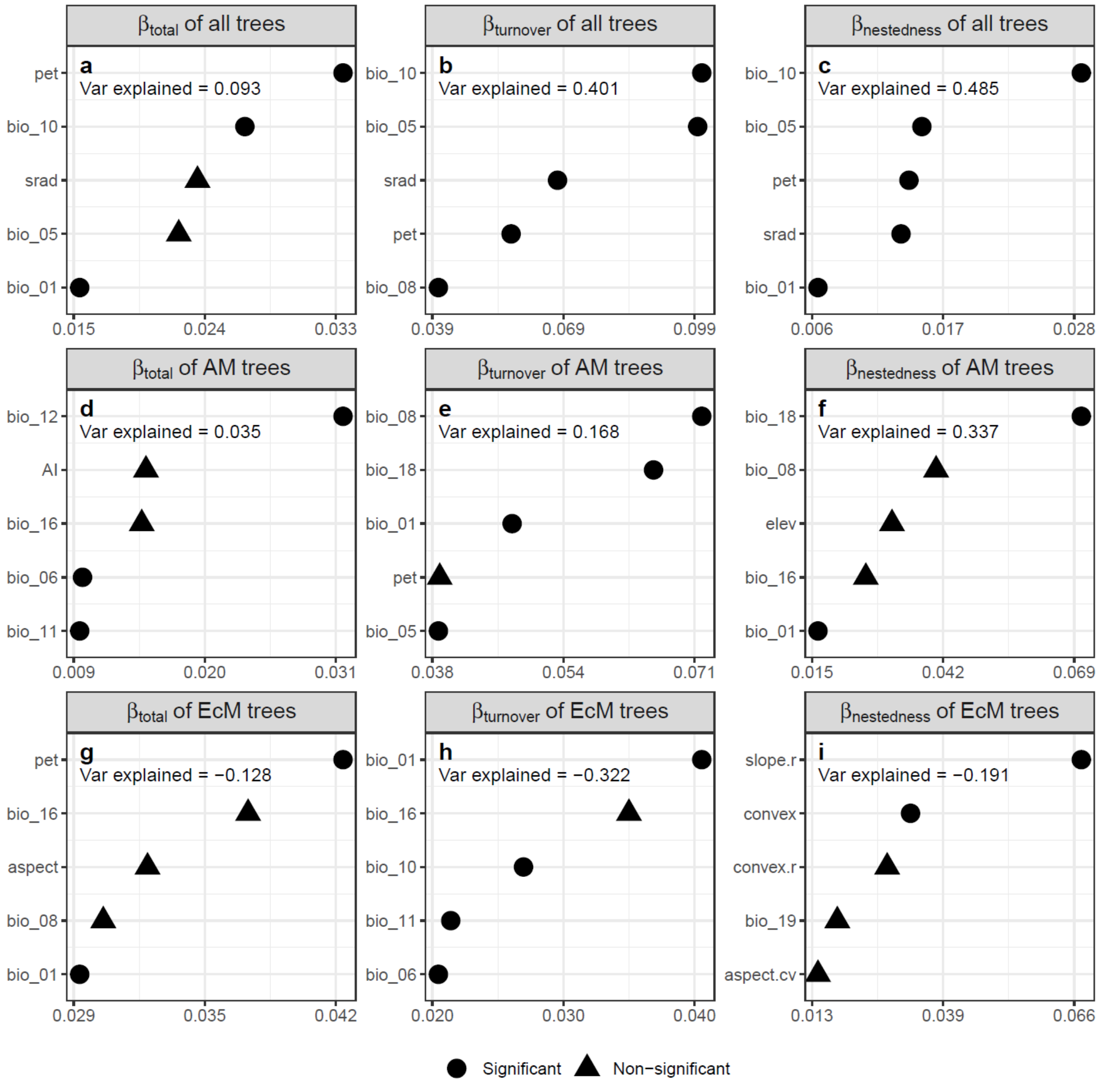

Figure S6 Relative importance of five most important environmental factors for total beta-diversity, species turnover, and species nestedness of all trees (a-c), AM trees (d-f), and EcM trees (g-i) at the scale of 50 m × 50 m. Total beta-diversity and its components are the mean values of 200 replicates of average pairwise beta-diversity and its component metrics calculated based on 15 randomly sampled quadrats of 50 m × 50 m from each forest plot. The relative importance of variables was ranked by the increase in node purity (horizontal axis). The proportion of variance displayed was that explained by all of 34 environmental variables. Circle points indicate significant importance of predictors whereas triangles indicate non-significant importance of predictors.

Figure S7 Partial dependence of total beta-diversity, species turnover, and species nestedness of all trees, AM trees, and EcM trees on each of 34 environmental variables at the scale of 10 m × 10 m (Supplementary Figs. 7.1-7.9), 20 m × 20 m (Supplementary Figs. 7.10-7.18), and 50 m × 50 m (Supplementary Figs. 7.19-7.27). Total beta-diversity and its components are the mean values of 200 replicates of average pairwise beta-diversity and its component metrics calculated based on 30, 15, and 15 randomly sampled quadrats of 10 m × 10 m, 20 m × 20 m, and 50 m × 50 m from each forest plot, respectively. Environmental variables were ranked according to the relative importance. Solid black lines indicate significant importance of predictors whereas dashed black lines indicate non-significant importance of predictors. The fine dashed red lines indicate linear fit of response variables against predictors, showing the direction of effects of predictors on the response variables.

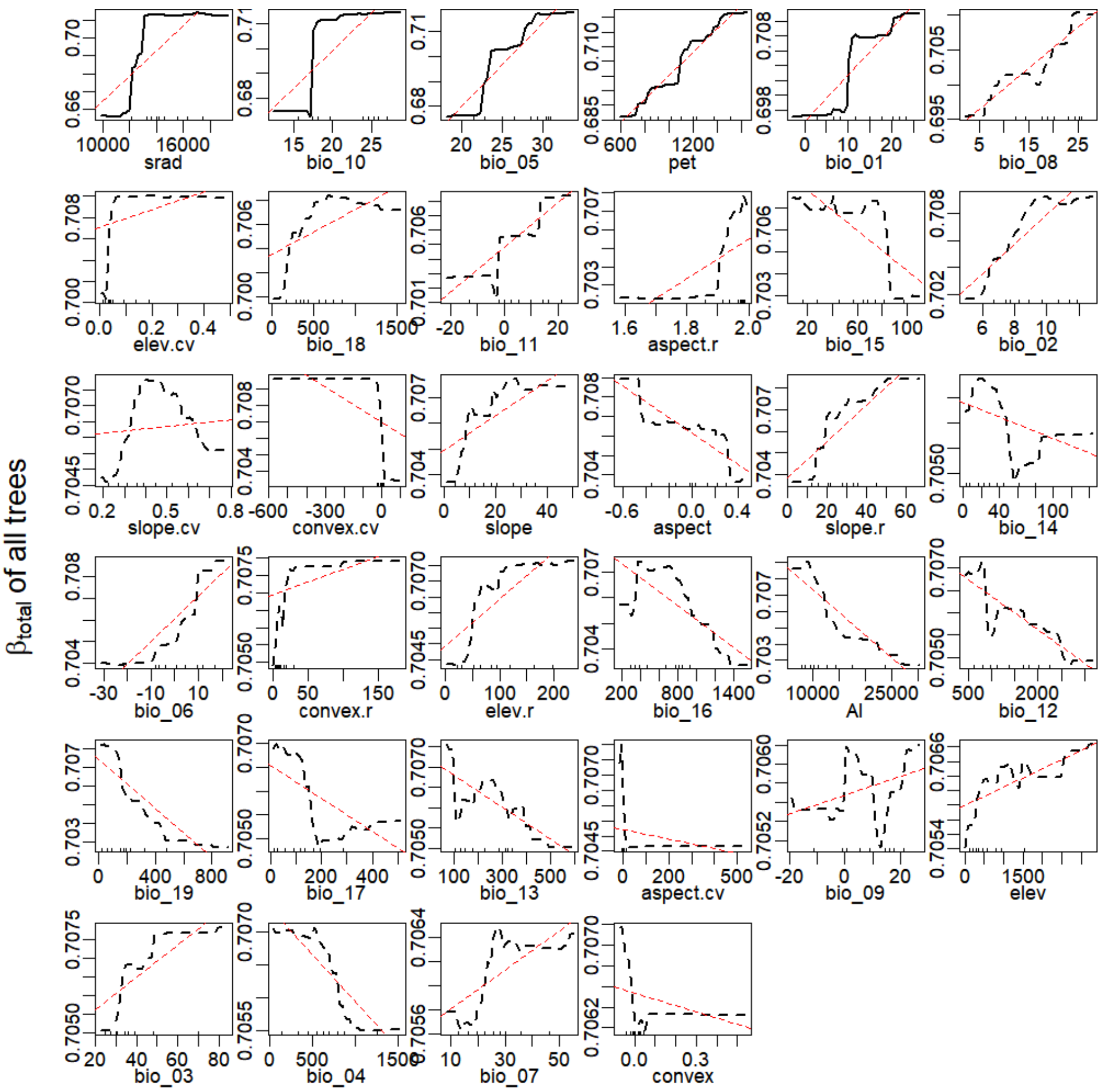

Figure S7.1 Partial dependence of total beta-diversity of all trees on 34 environmental variables at the scale of 10 m × 10 m.

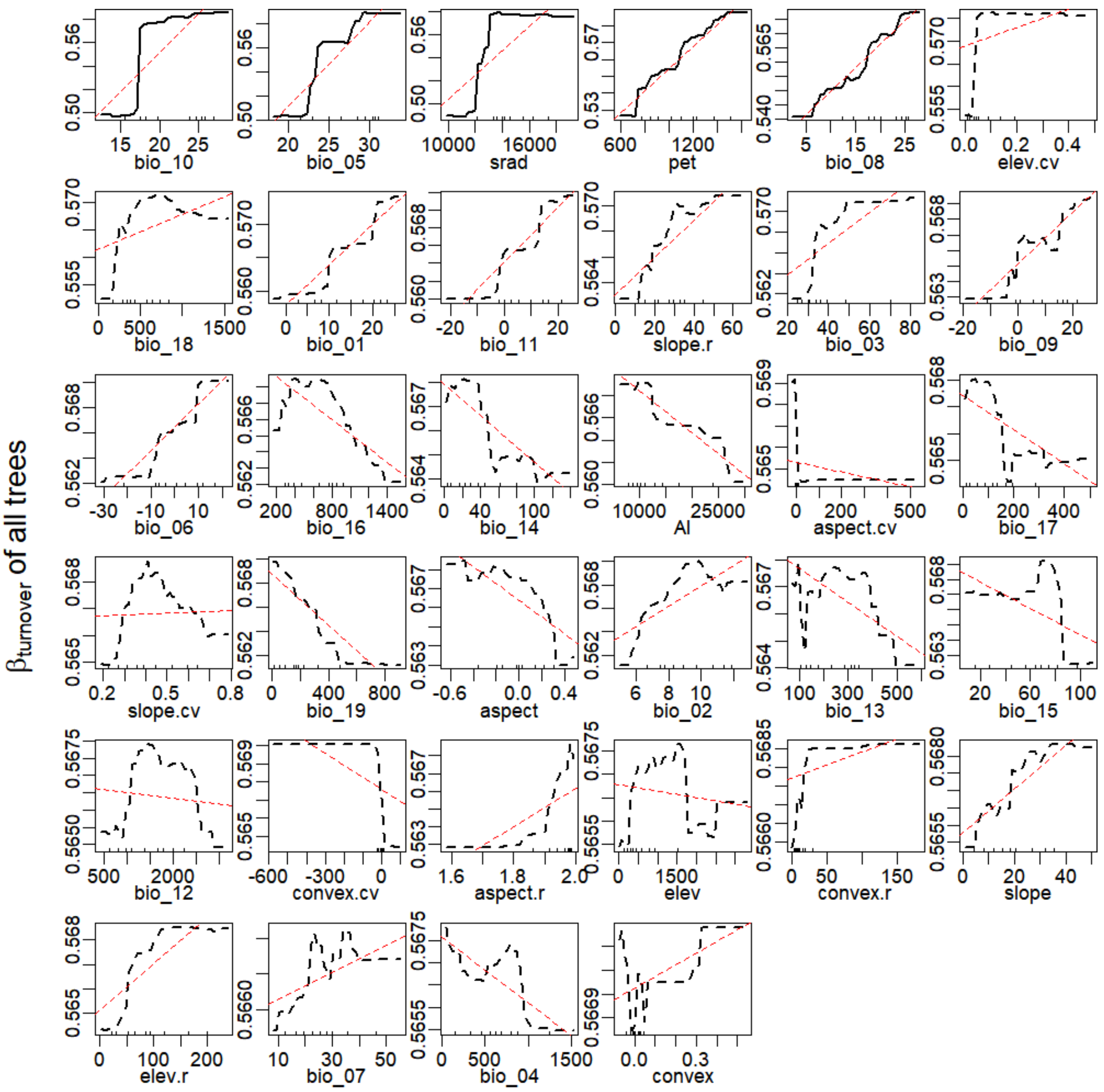

Figure S7.2 Partial dependence of turnover component of all trees on each of 34 environmental variables at the scale of 10 m × 10 m.

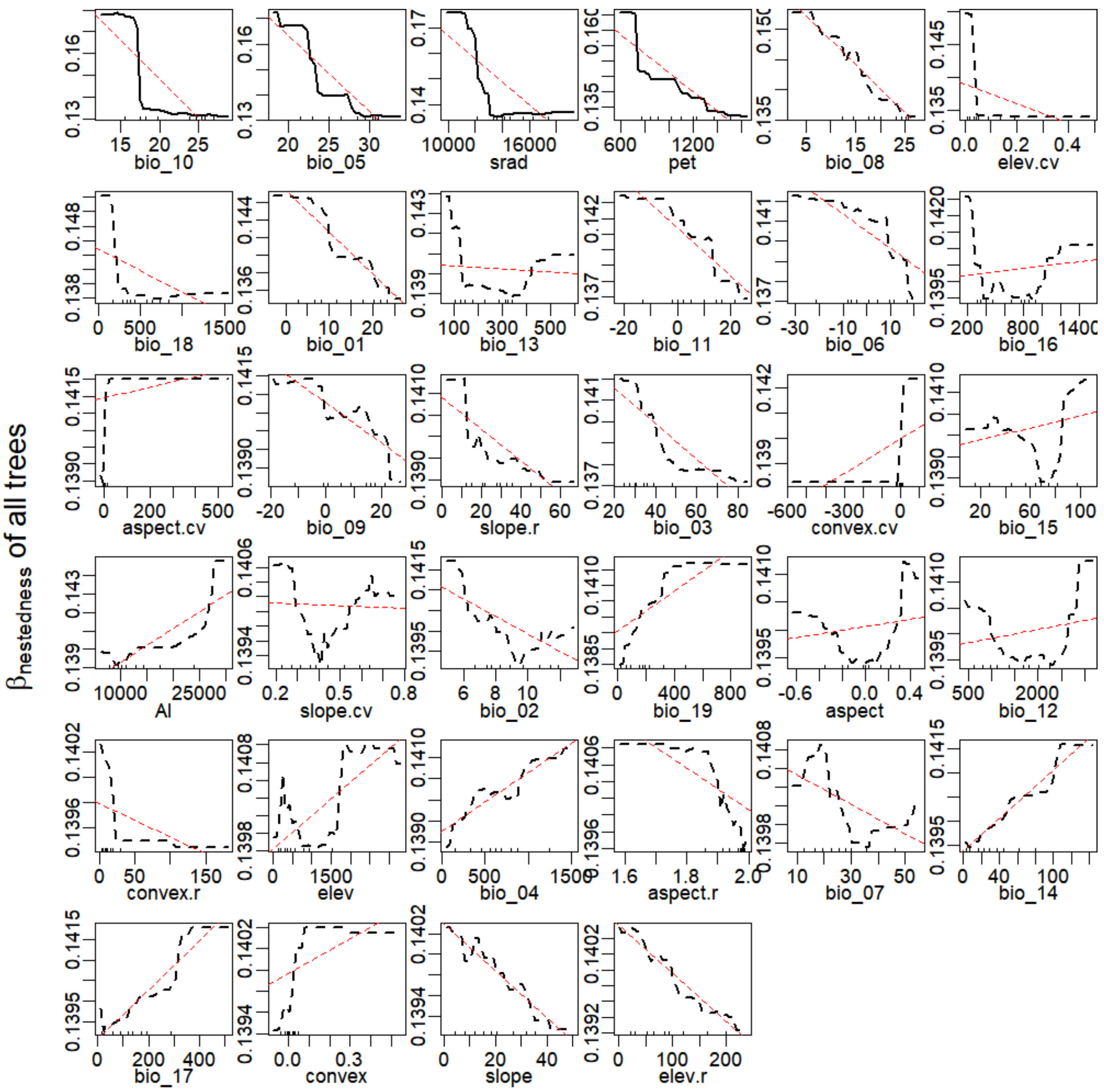

Figure S7.3 Partial dependence of nestedness component of all trees on each of 34 environmental variables at the scale of 10 m × 10 m.

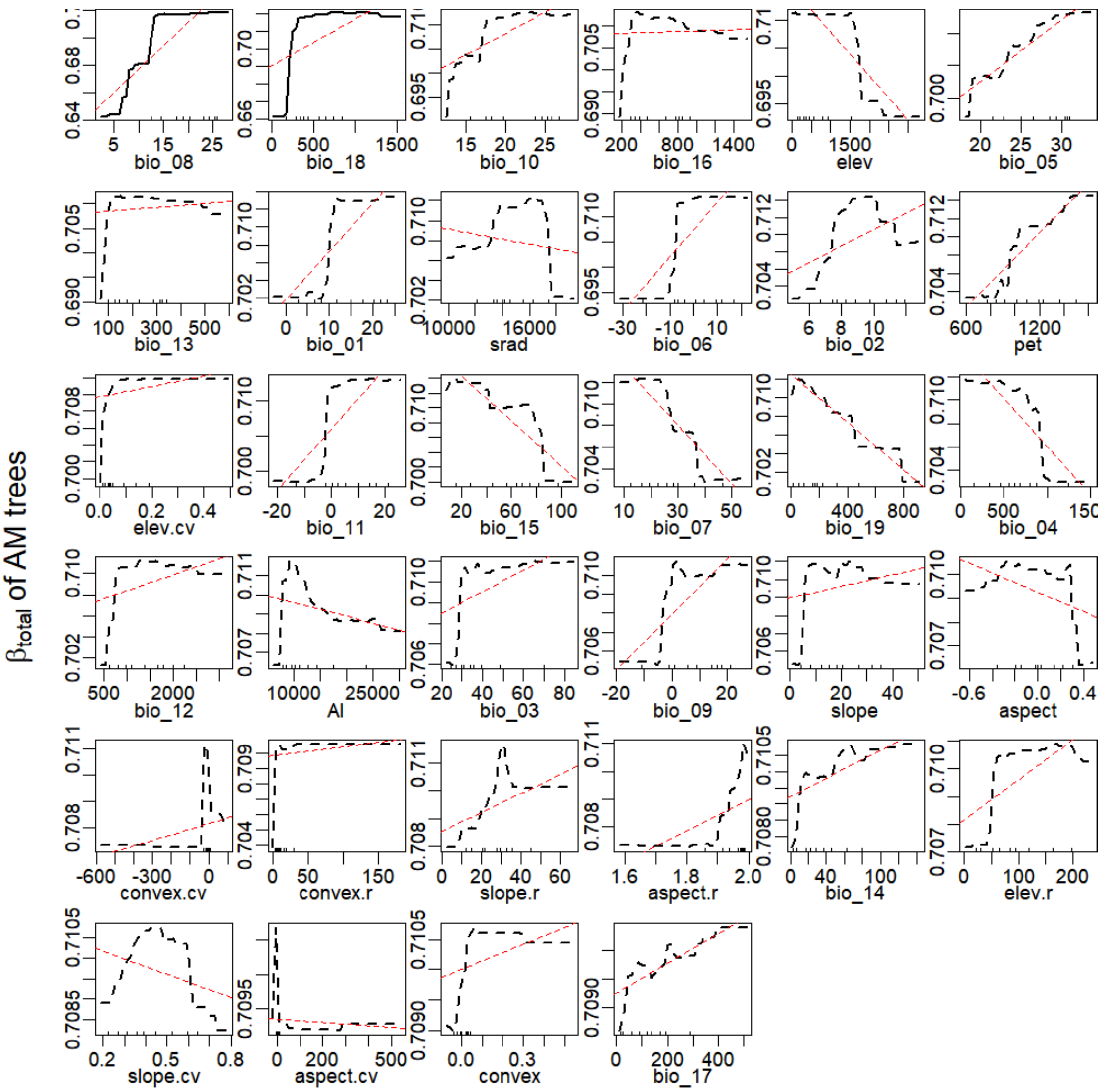

Figure S7.4 Partial dependence of total beta-diversity of AM trees on each of 34 environmental variables at the scale of 10 m × 10 m.

.

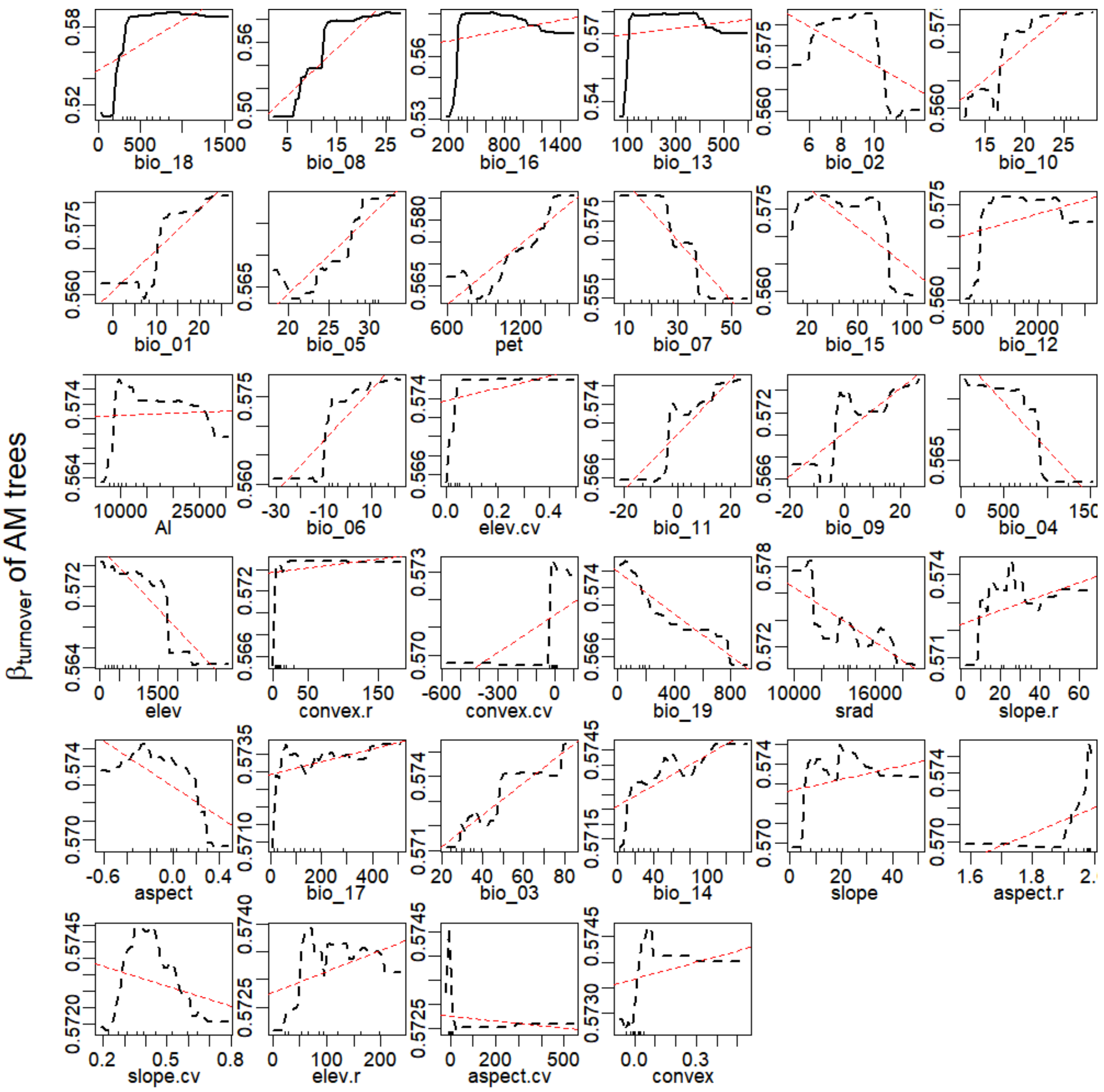

Figure S7.5 Partial dependence of the turnover component of AM trees on each of 34 environmental variables at the scale of 10 m × 10 m.

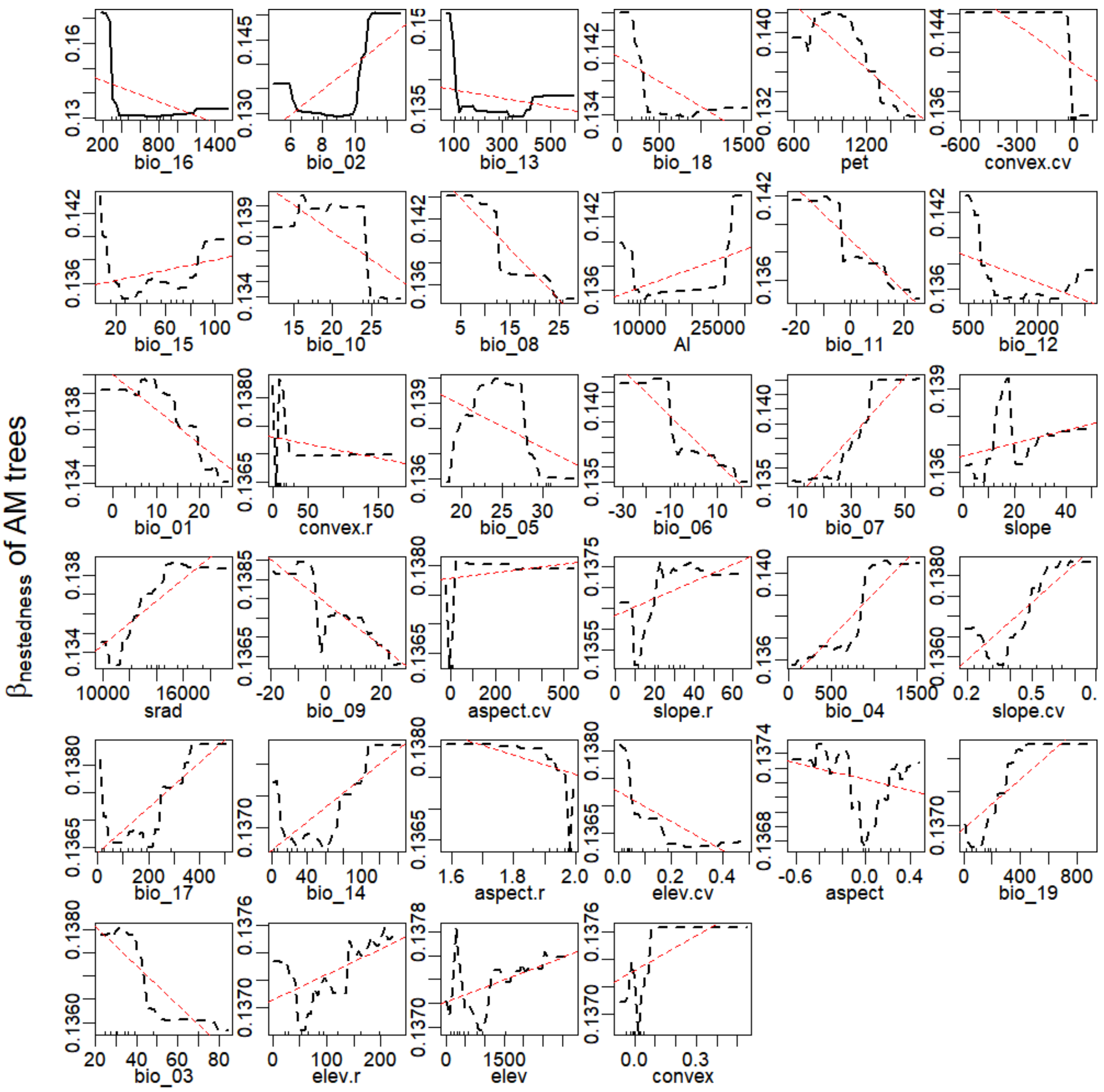

Figure S7.6 Partial dependence of the nestedness component of AM trees on each of 34 environmental variables at the scale of 10 m × 10 m.

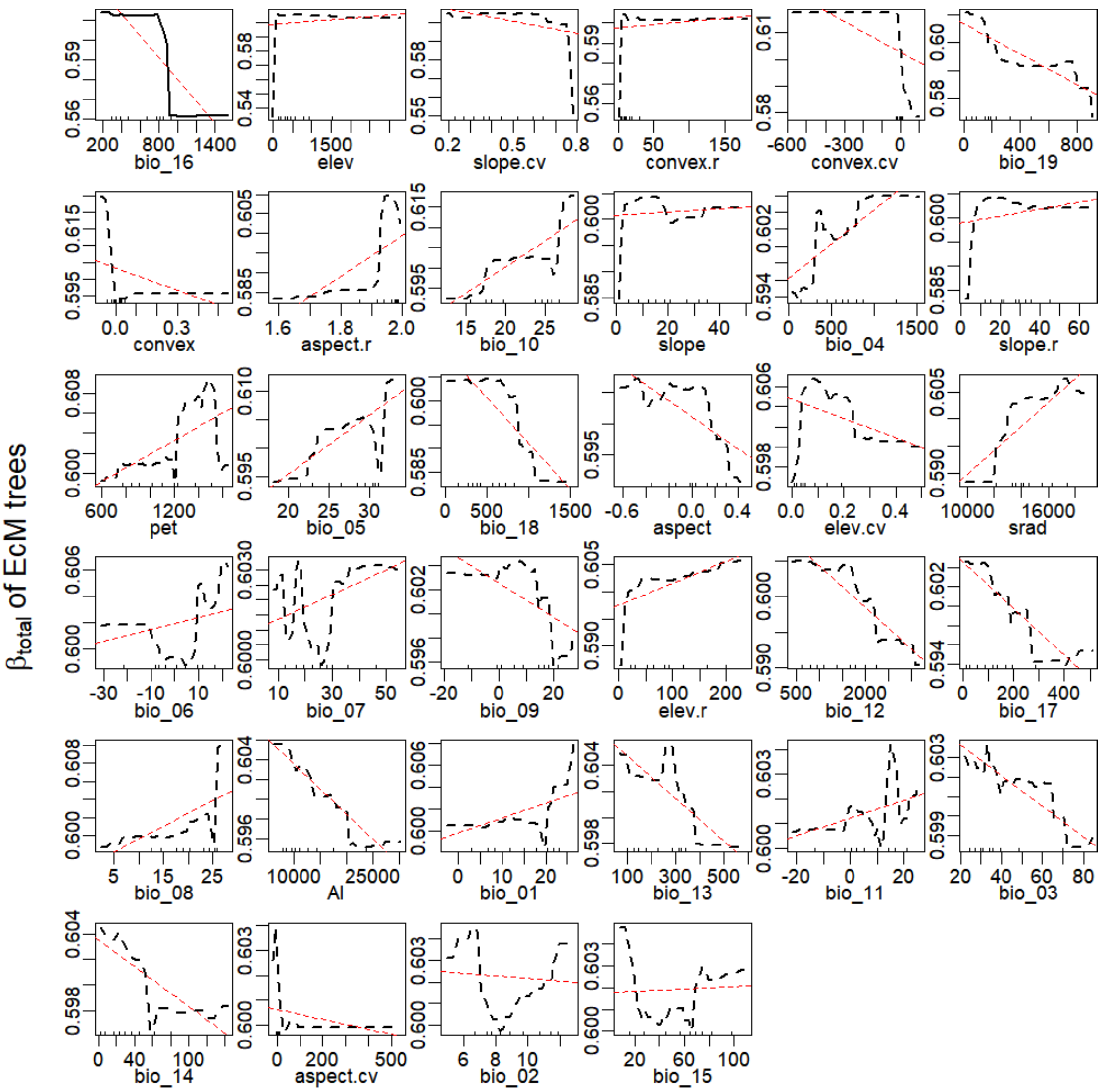

Figure S7.7 Partial dependence of total beta-diversity of EcM trees on each of 34 environmental variables at the scale of 10 m × 10 m.

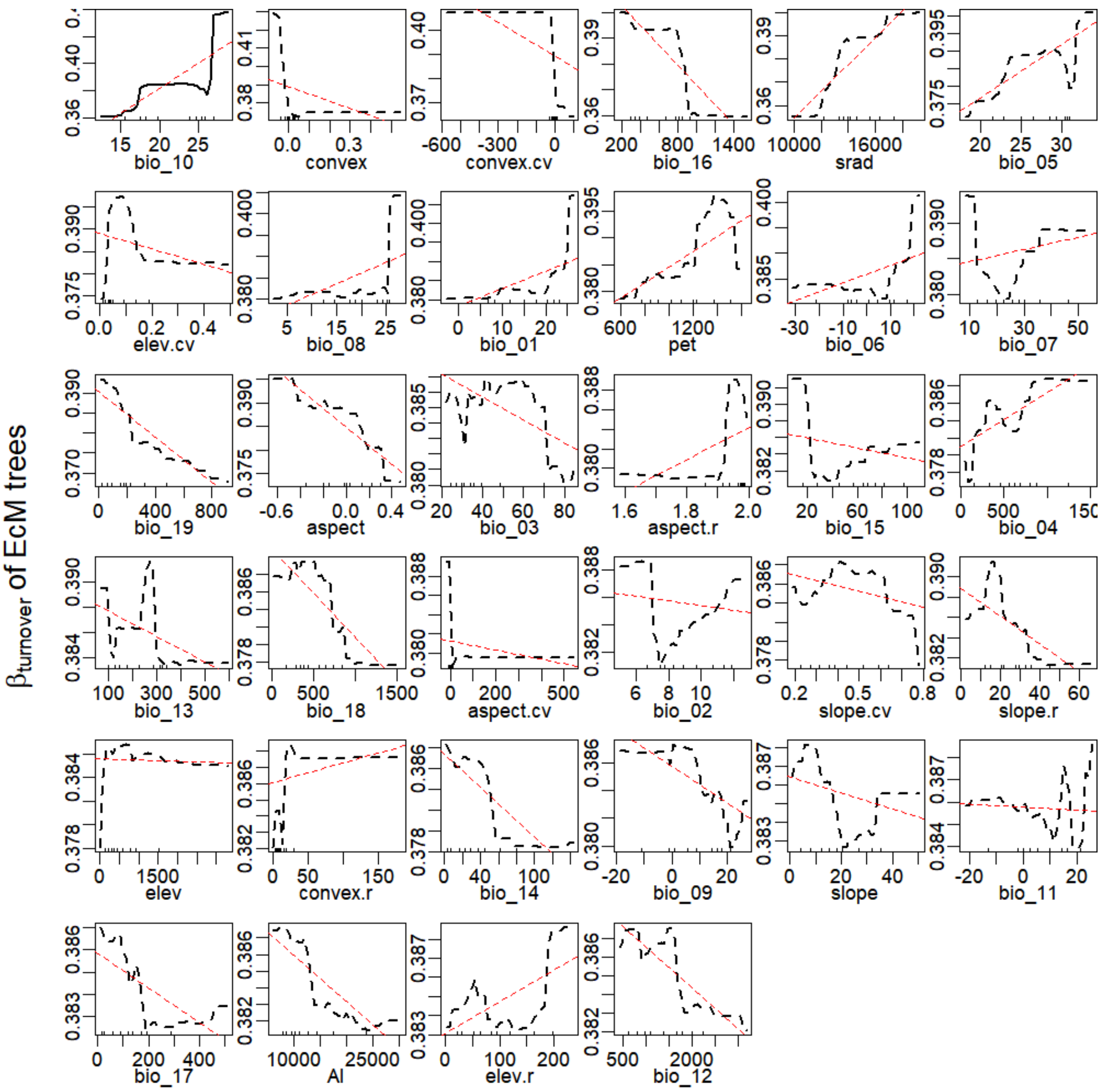

Figure S7.8 Partial dependence of the turnover component of EcM trees on each of 34 environmental variables at the scale of 10 m × 10 m.

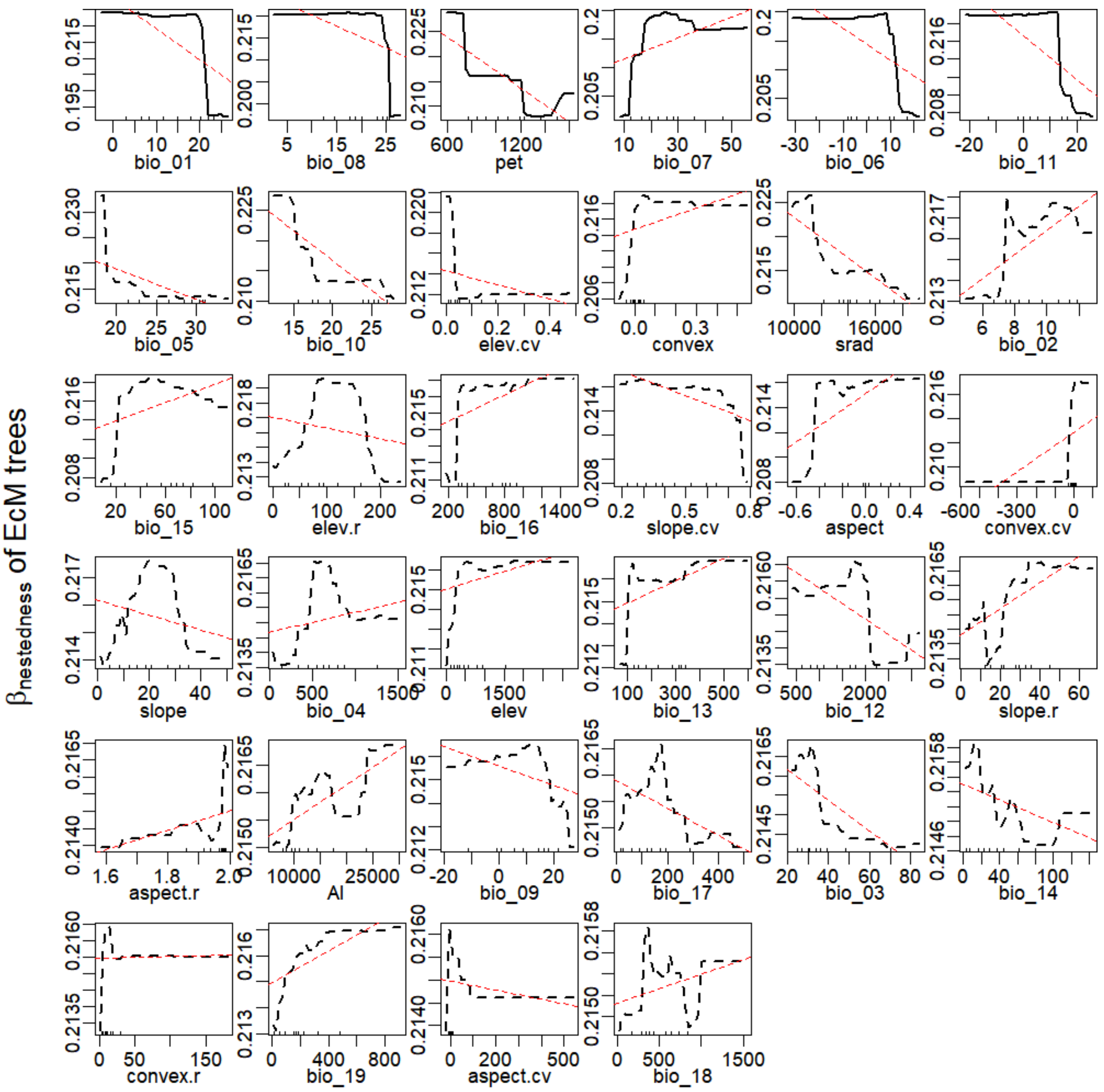

Figure S7.9 Partial dependence of the nestedness component of EcM trees on each of 34 environmental variables at the scale of 10 m × 10 m.

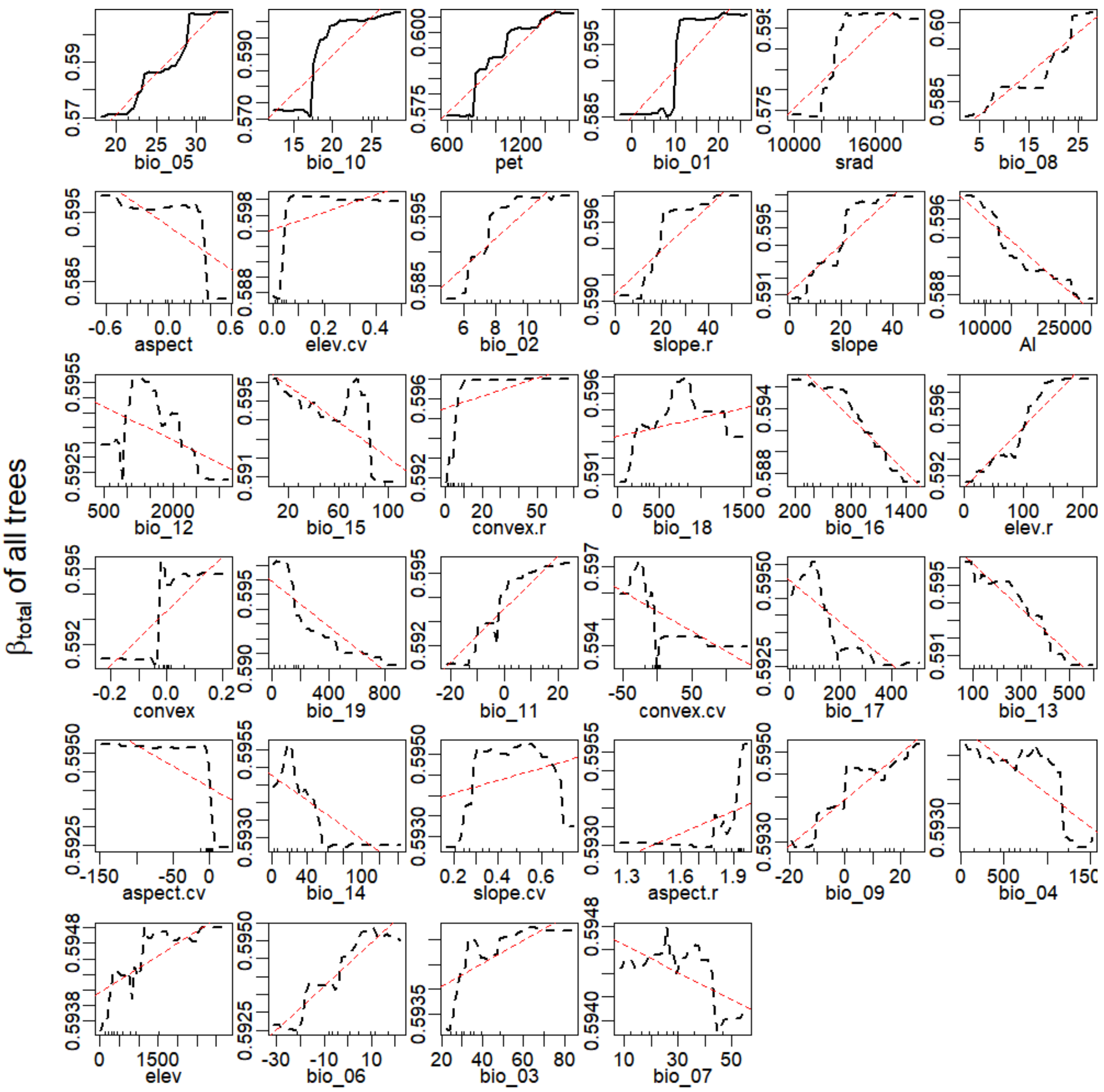

Figure S7.10 Partial dependence of total beta-diversity of all trees on 34 environmental variables at the scale of 20 m × 20 m.

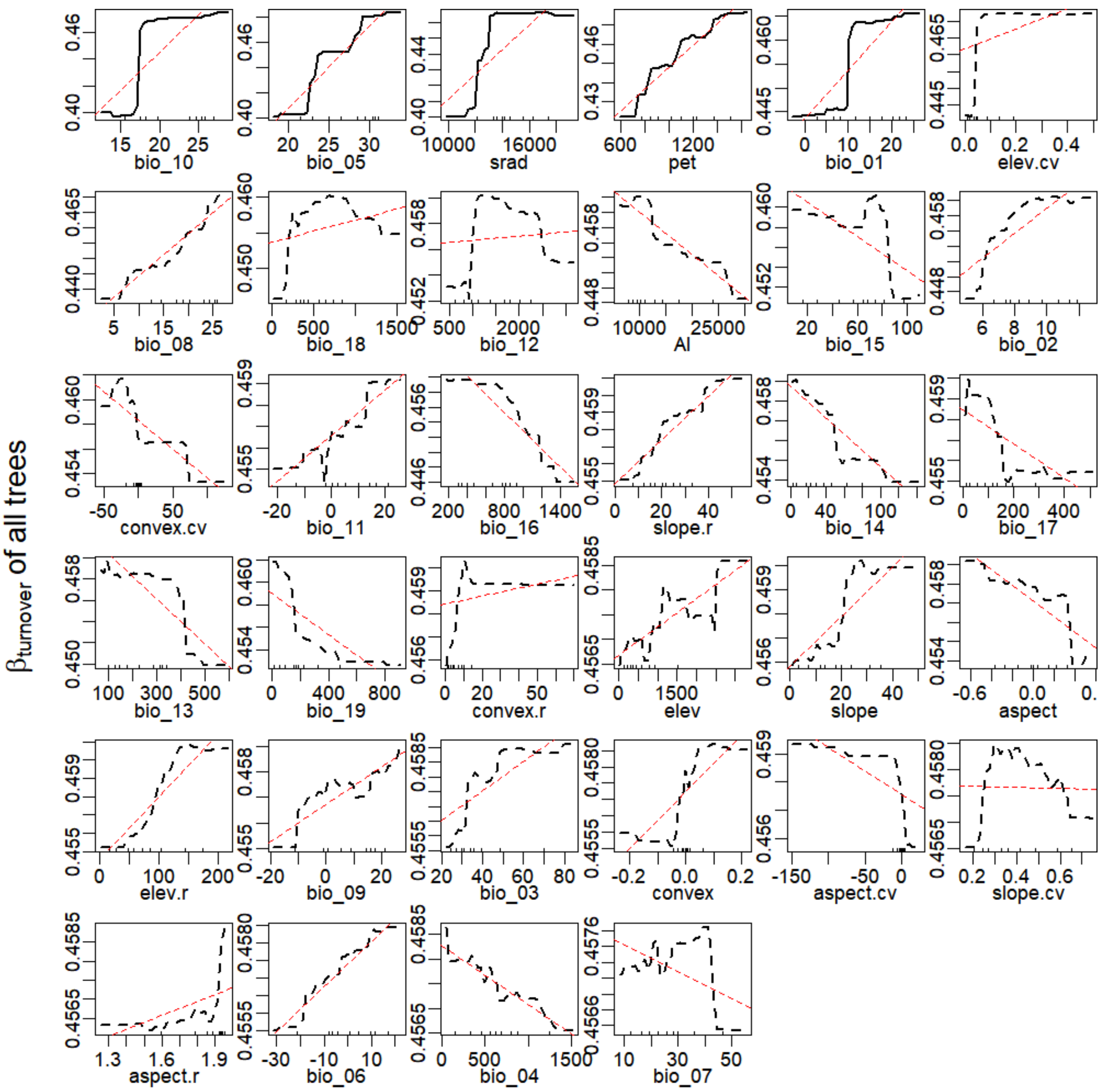

Figure S7.11 Partial dependence of turnover component of all trees on each of 34 environmental variables at the scale of 20 m × 20 m.

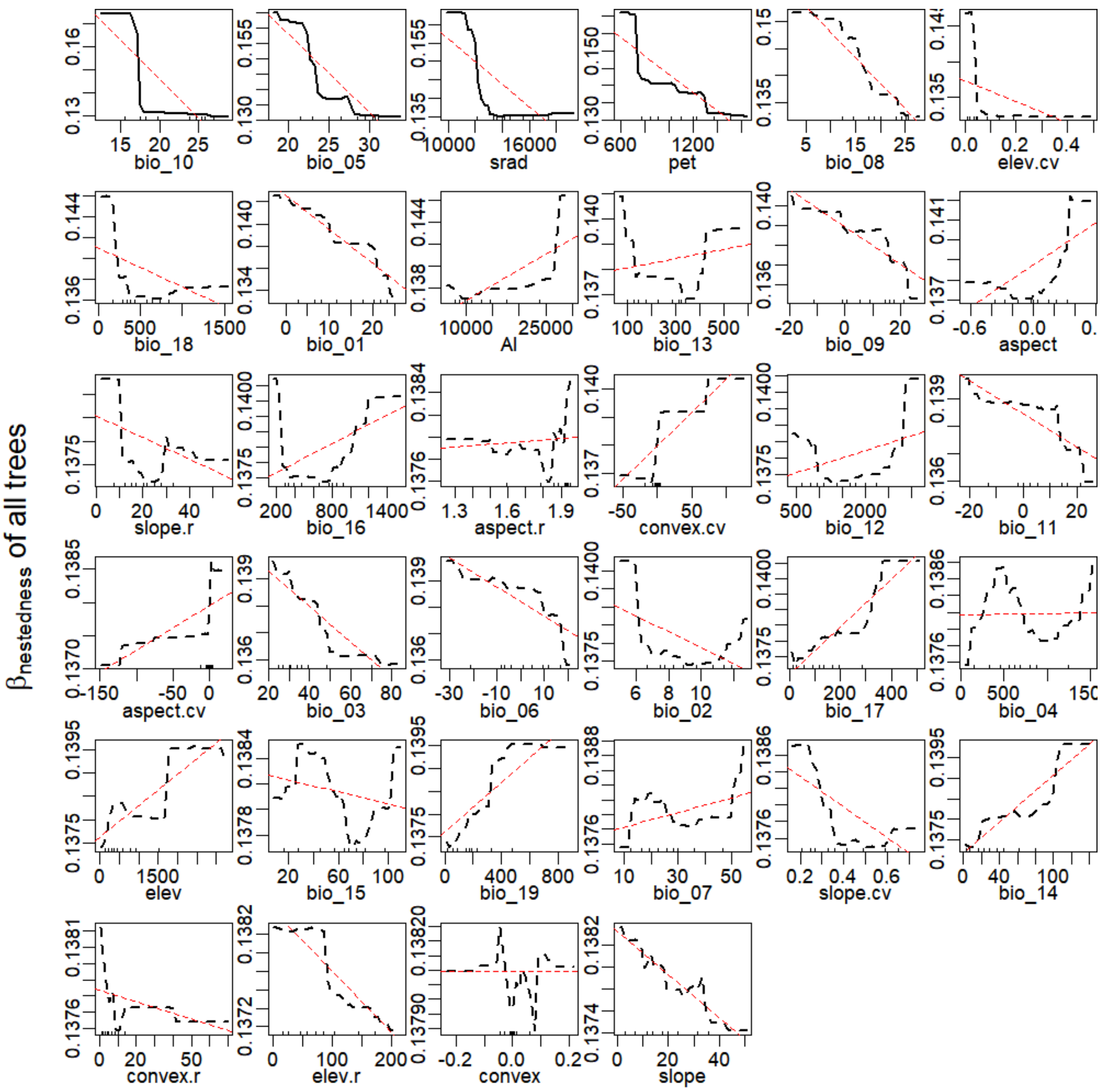

Figure S7.12 Partial dependence of nestedness component of all trees on each of 34 environmental variables at the scale of 20 m × 20 m.

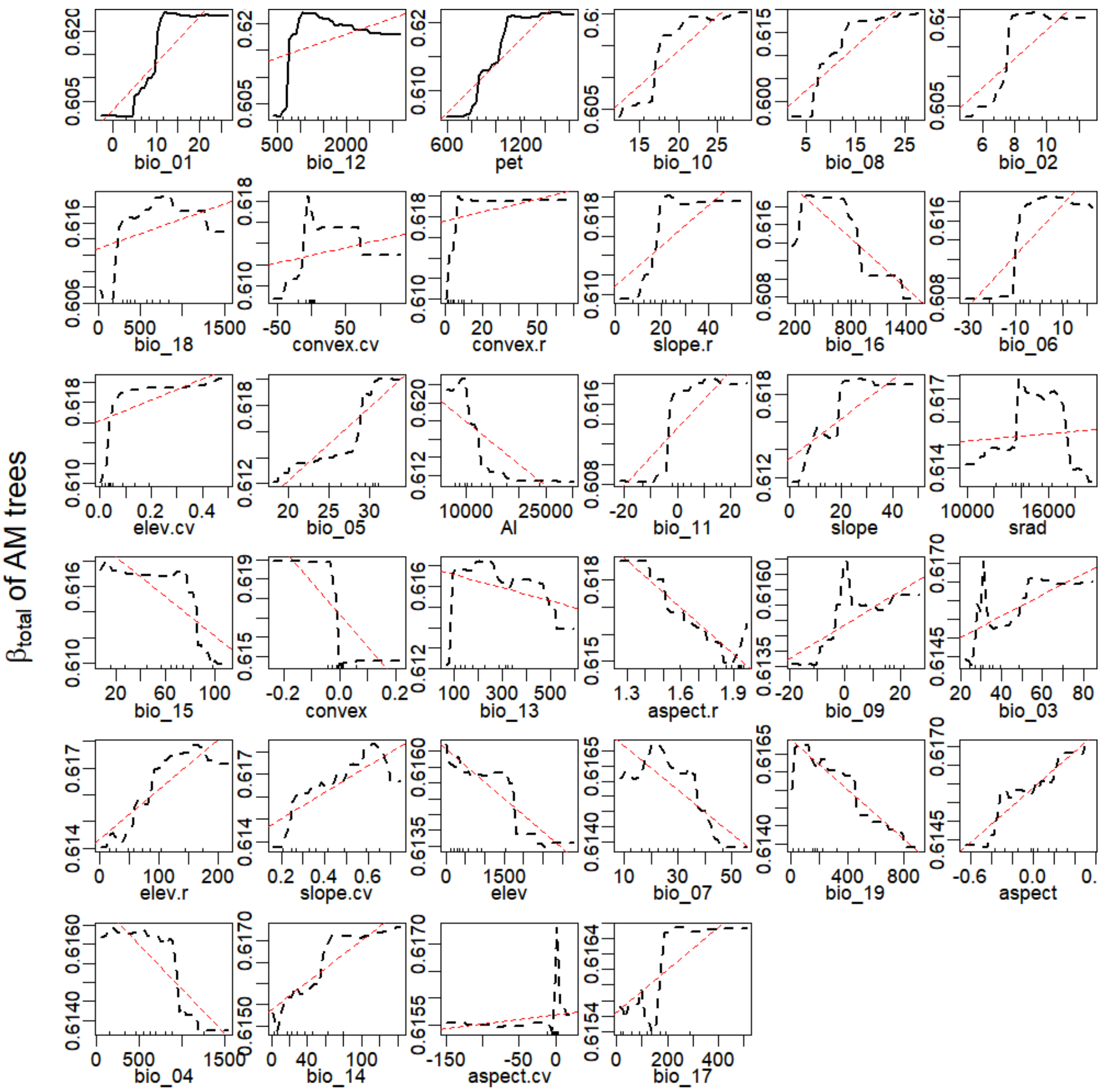

Figure S7.13 Partial dependence of total beta-diversity of AM trees on each of 34 environmental variables at the scale of 20 m × 20 m.

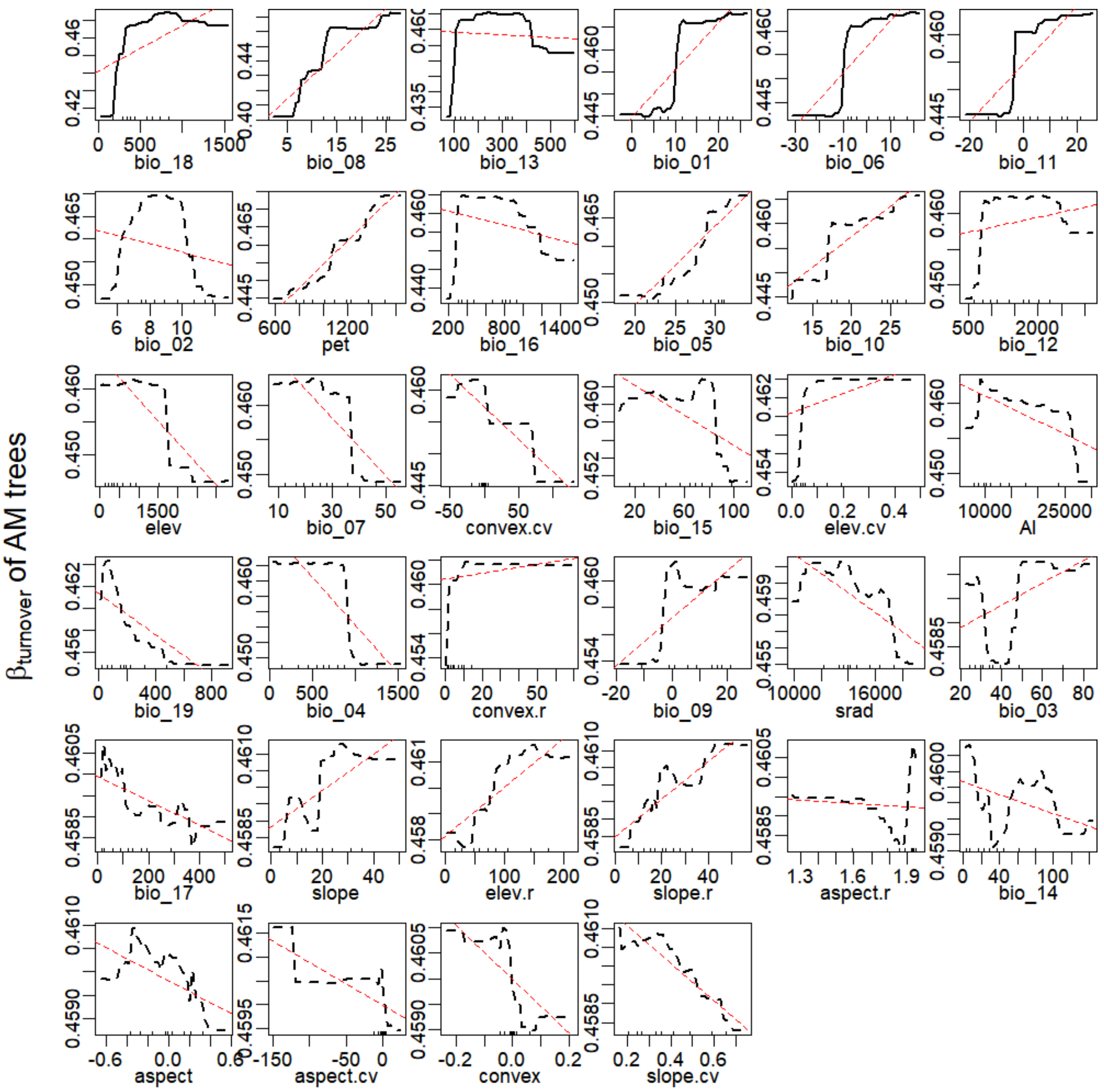

Figure S7.14 Partial dependence of the turnover component of AM trees on each of 34 environmental variables at the scale of 20 m × 20 m.

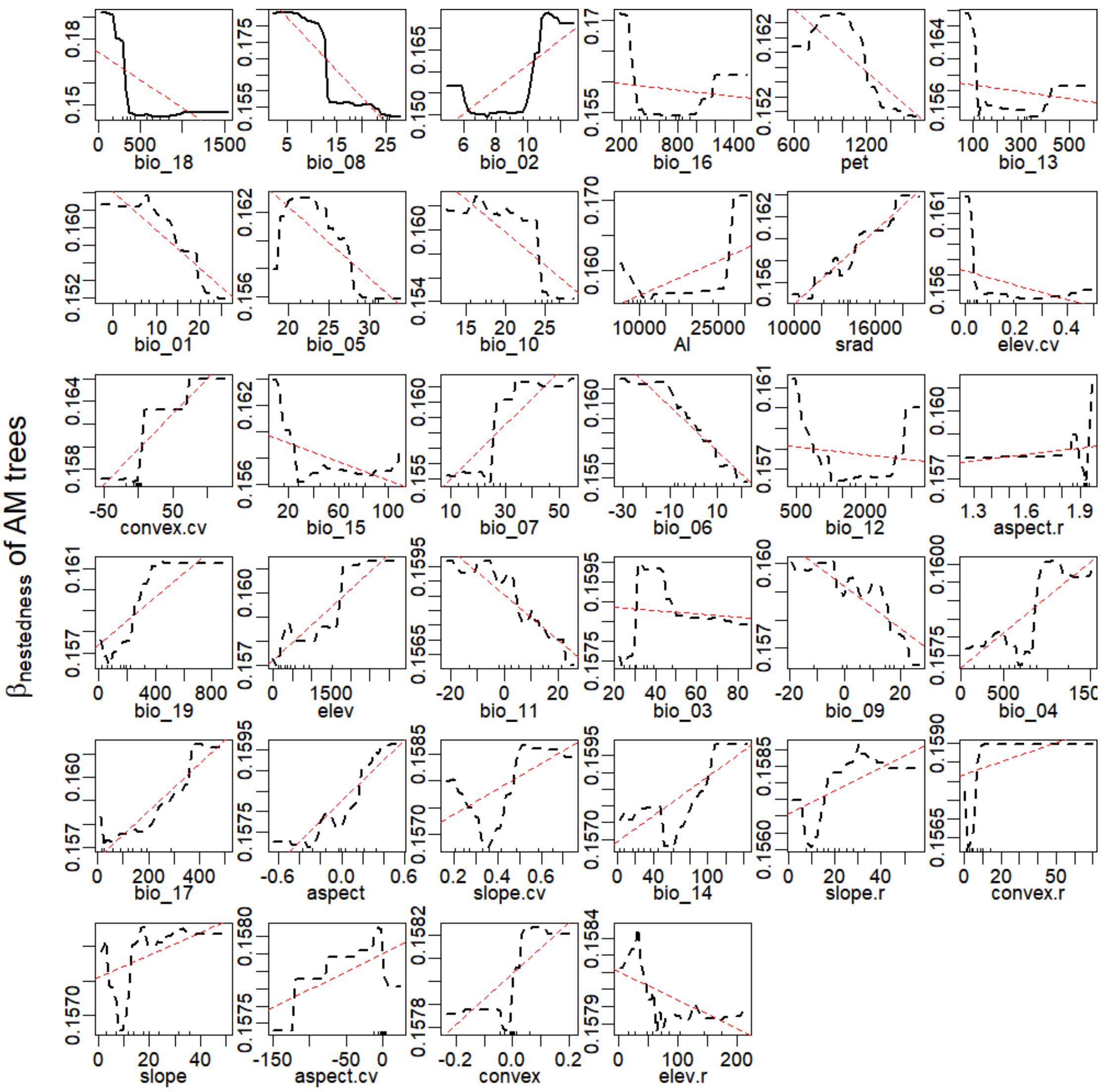

Figure S7.15 Partial dependence of the nestedness component of AM trees on each of 34 environmental variables at the scale of 20 m × 20 m.

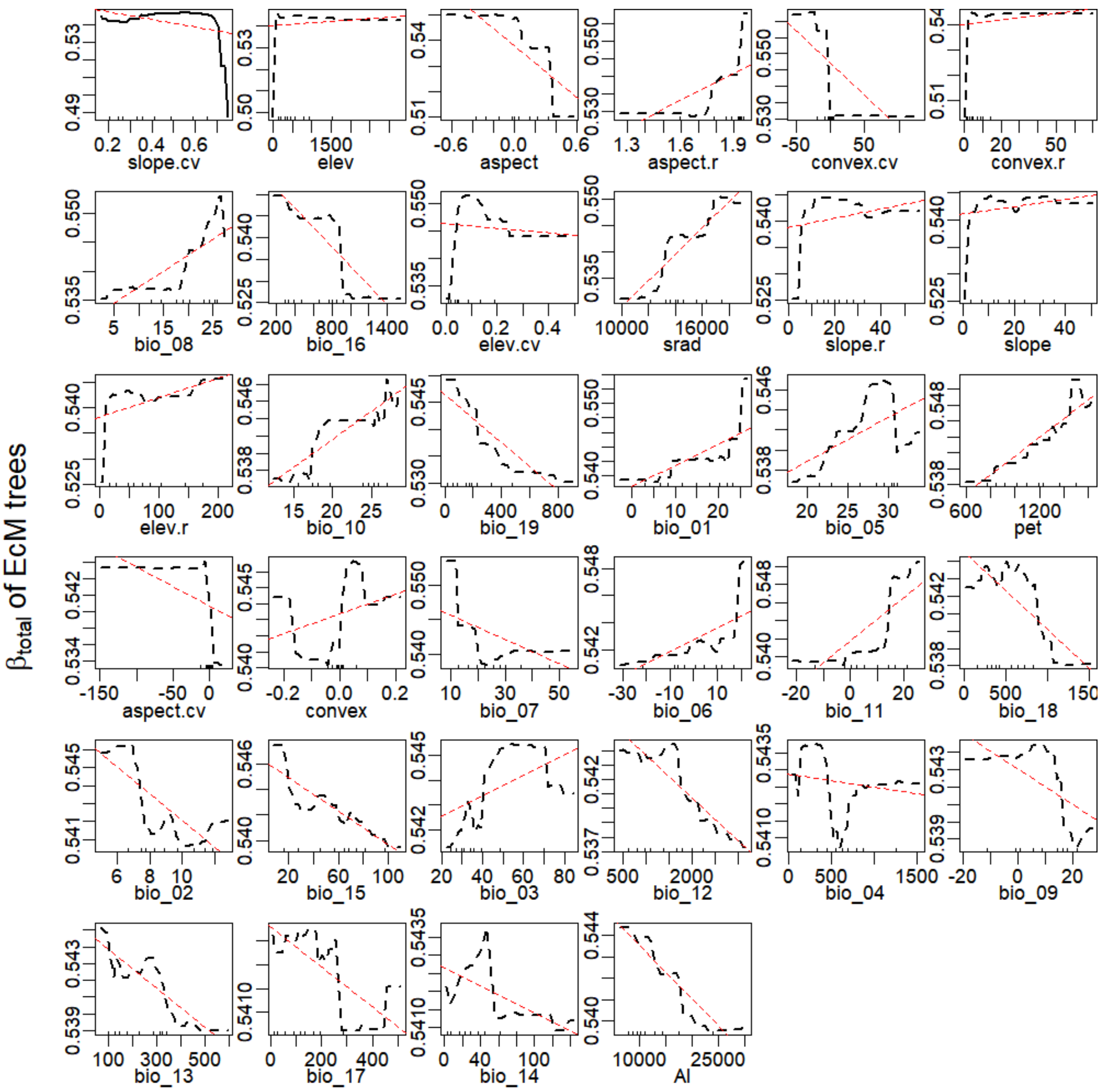

Figure S7.16 Partial dependence of total beta-diversity of EcM trees on each of 34 environmental variables at the scale of 20 m × 20 m.

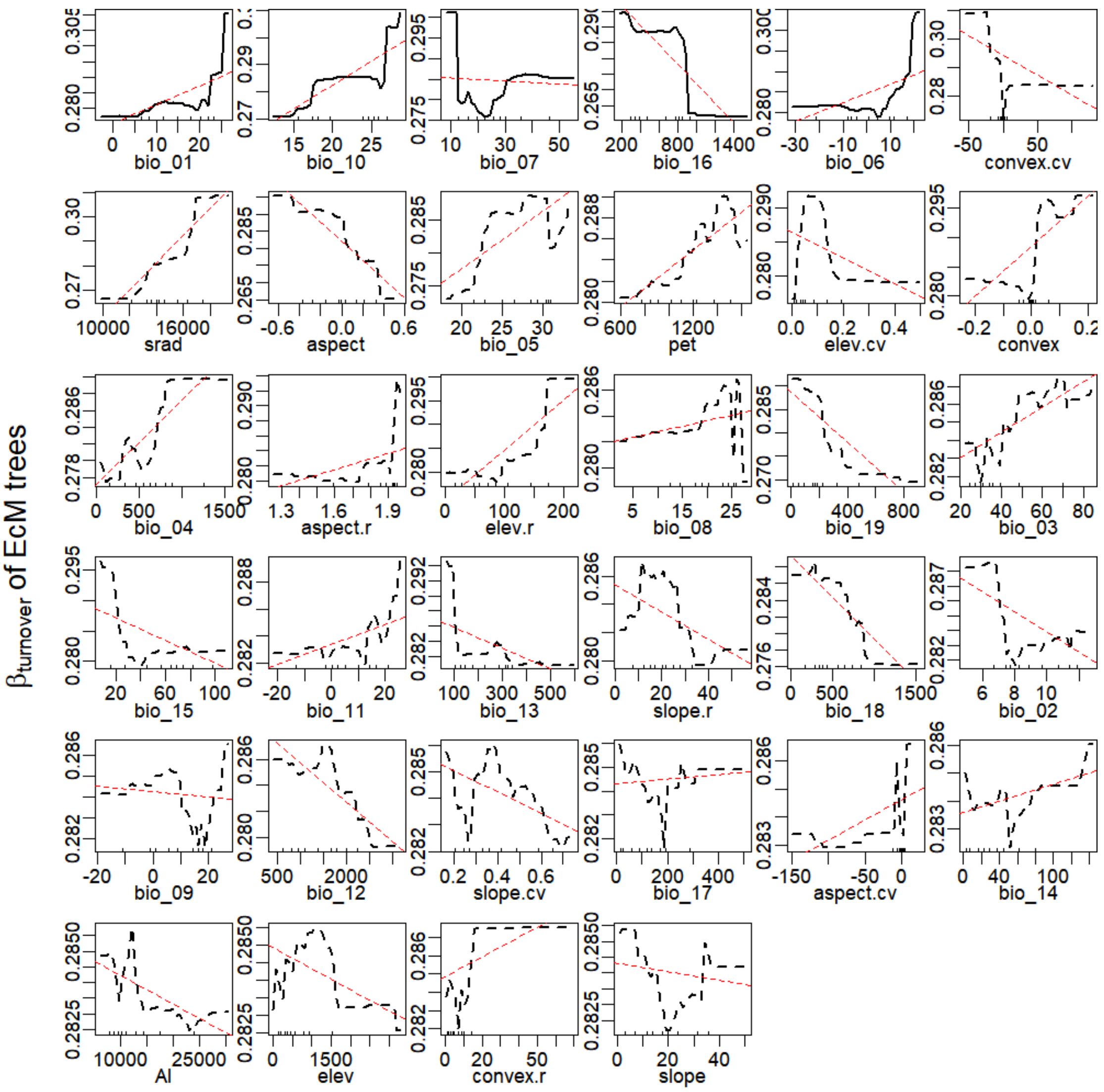

Figure S7.17 Partial dependence of the turnover component of EcM trees on each of 34 environmental variables at the scale of 20 m × 20 m.

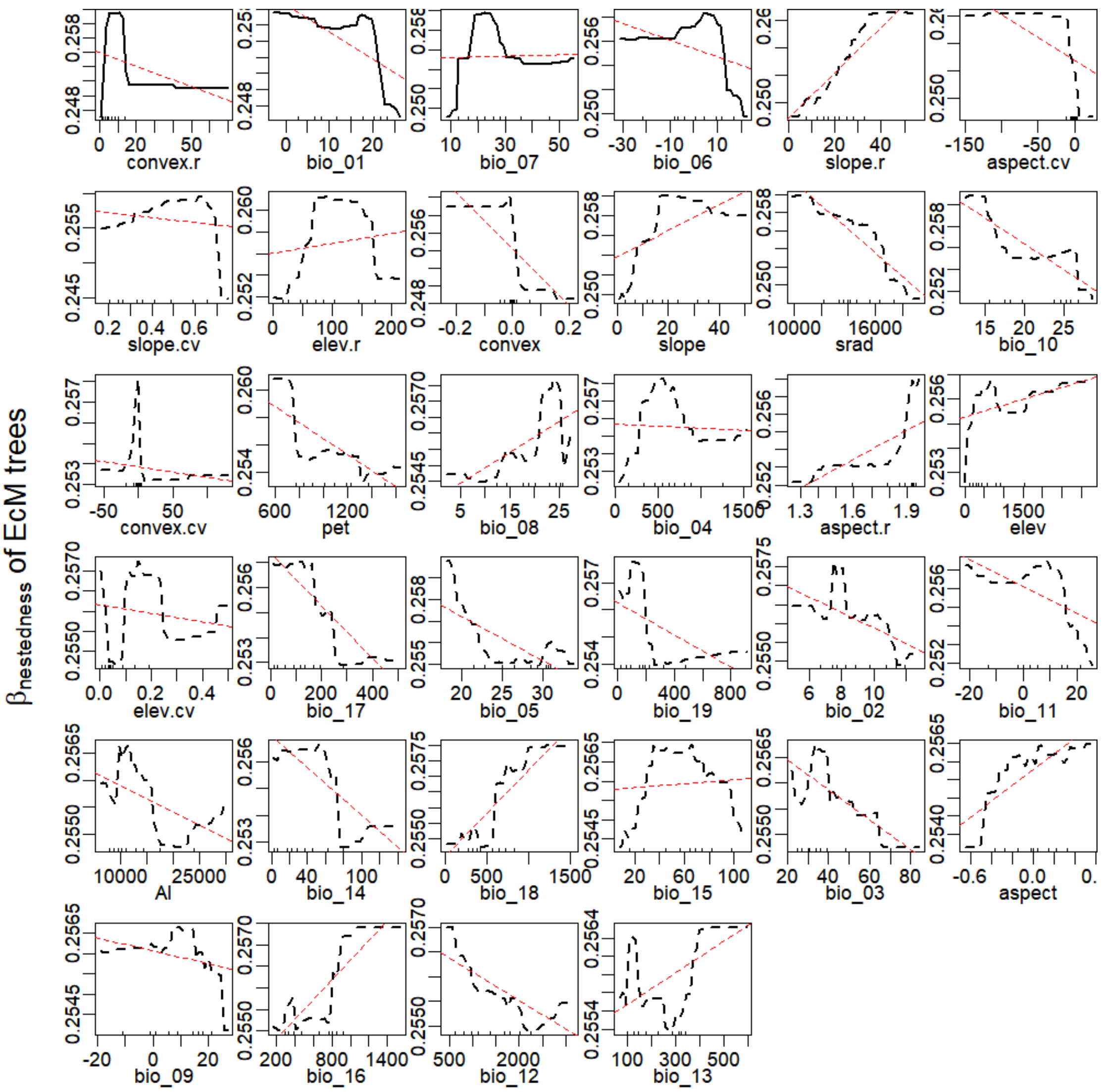

Figure S7.18 Partial dependence of the nestedness component of EcM trees on each of 34 environmental variables at the scale of 20 m × 20 m.

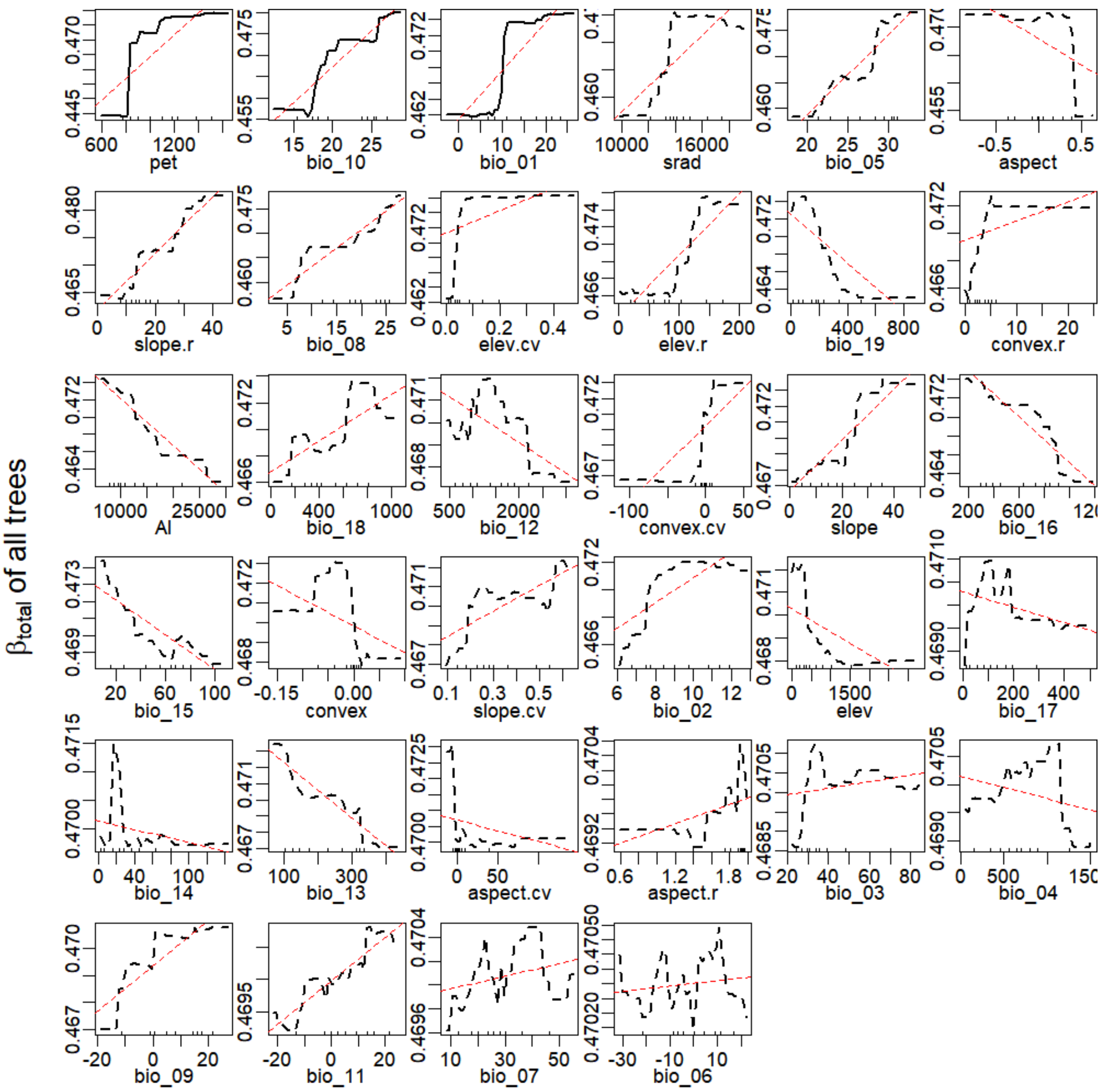

Figure S7.19 Partial dependence of total beta-diversity of all trees on 34 environmental variables at the scale of 50 m × 50 m.

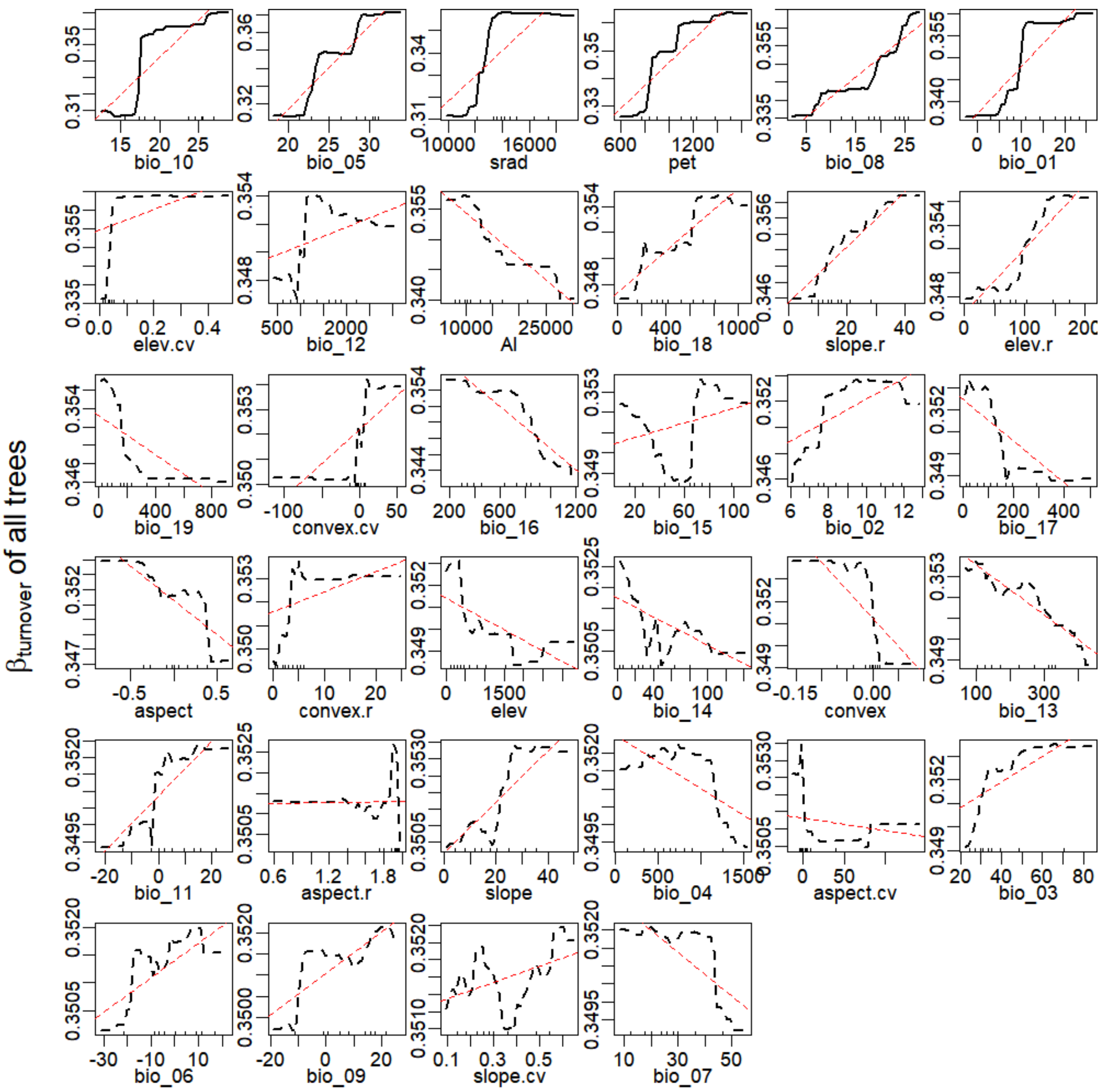

Figure S7.20 Partial dependence of turnover component of all trees on each of 34 environmental variables at the scale of 50 m × 50 m.

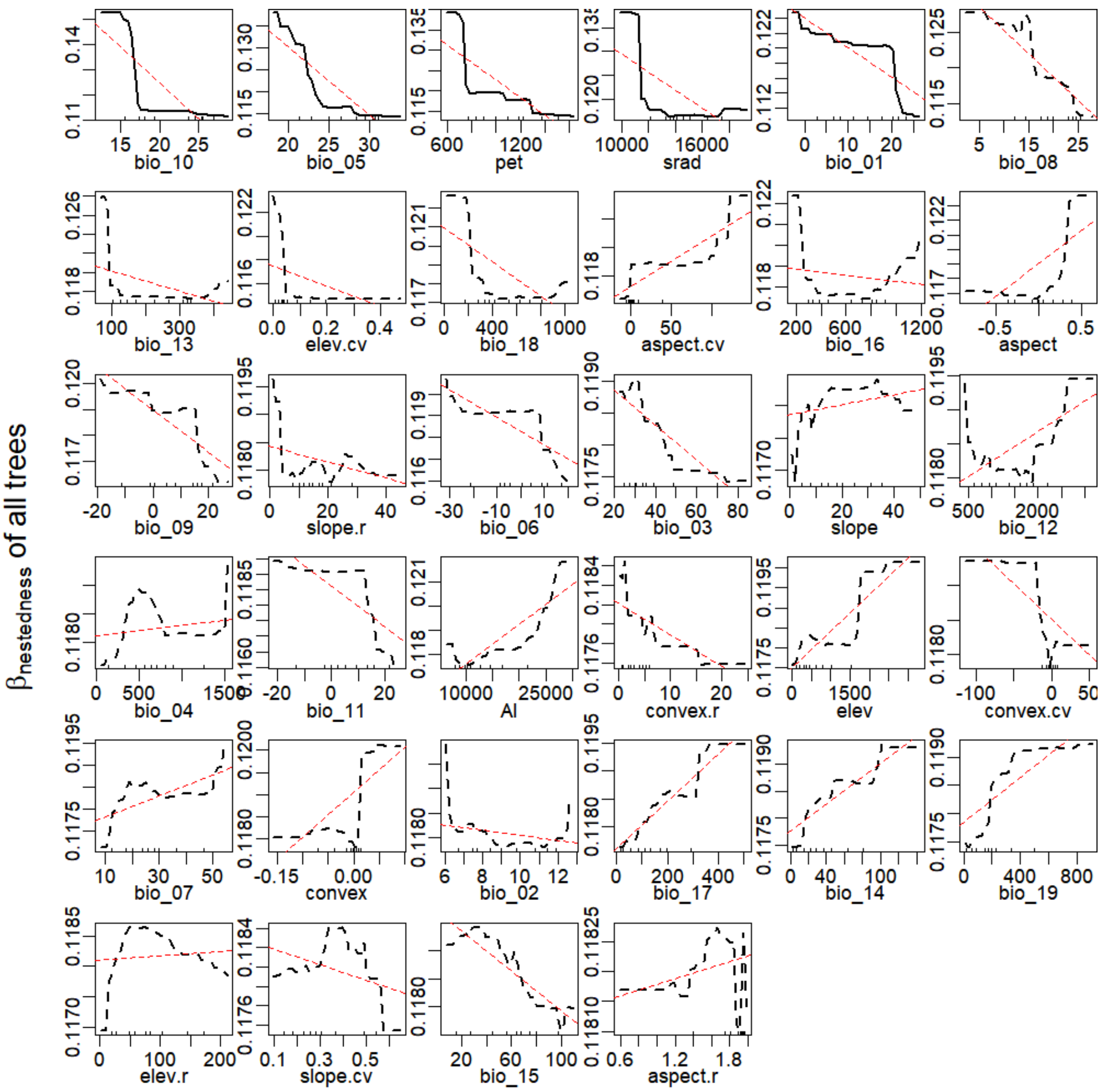

Figure S7.21 Partial dependence of nestedness component of all trees on each of 34 environmental variables at the scale of 50 m × 50 m.

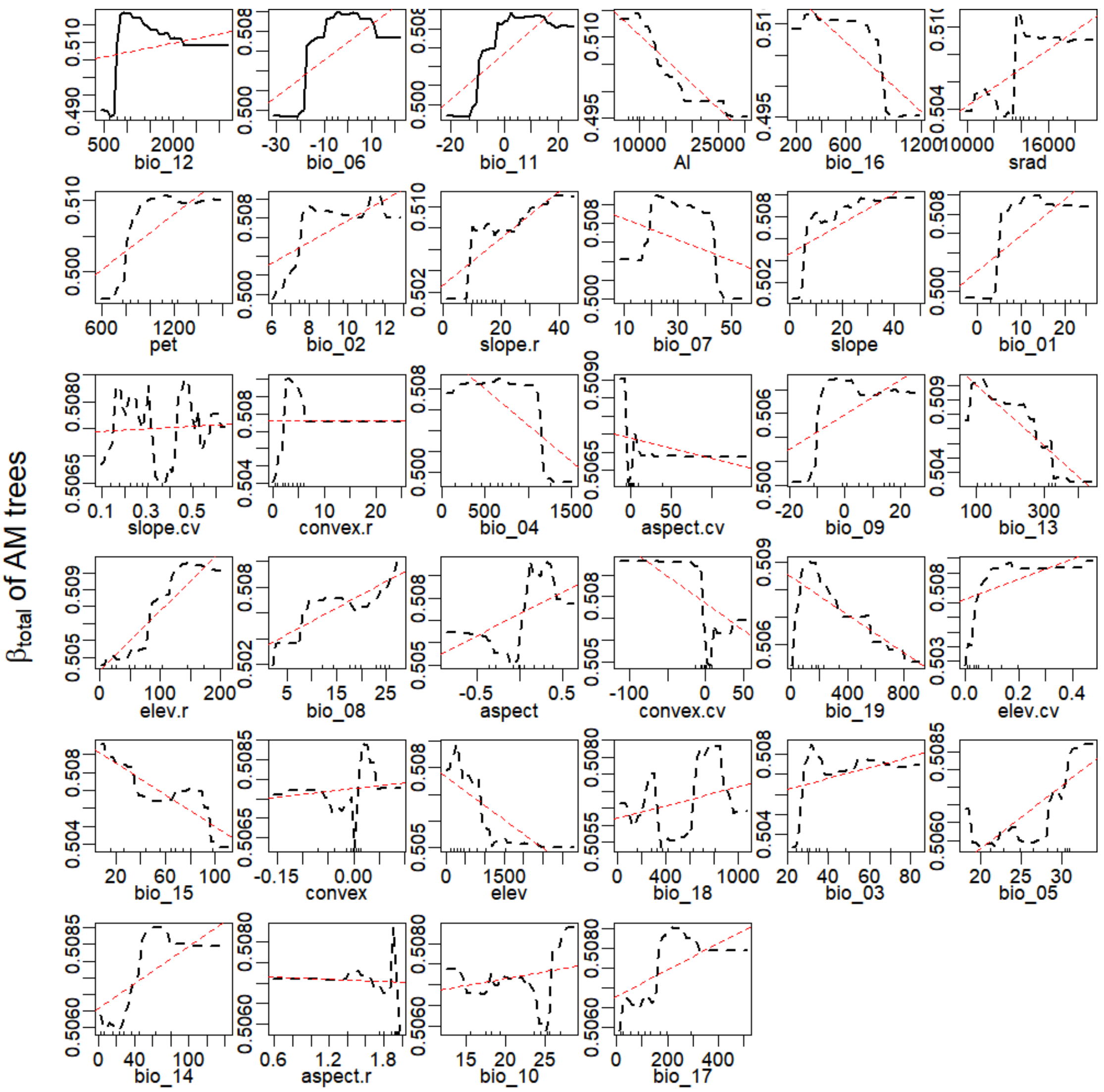

Figure S7.22 Partial dependence of total beta-diversity of AM trees on each of 34 environmental variables at the scale of 50 m × 50 m.

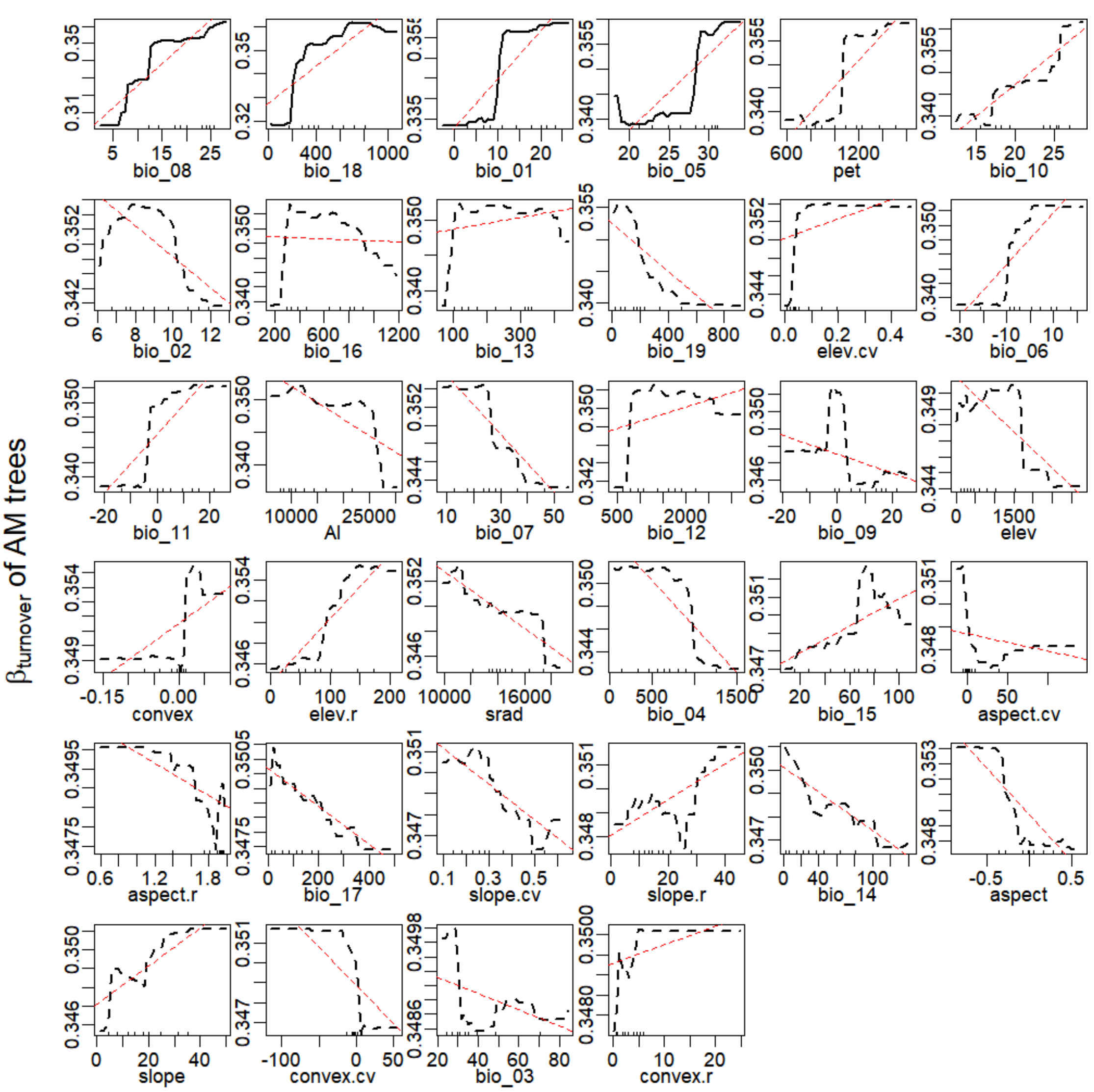

Figure S7.23 Partial dependence of the turnover component of AM trees on each of 34 environmental variables at the scale of 50 m × 50 m.

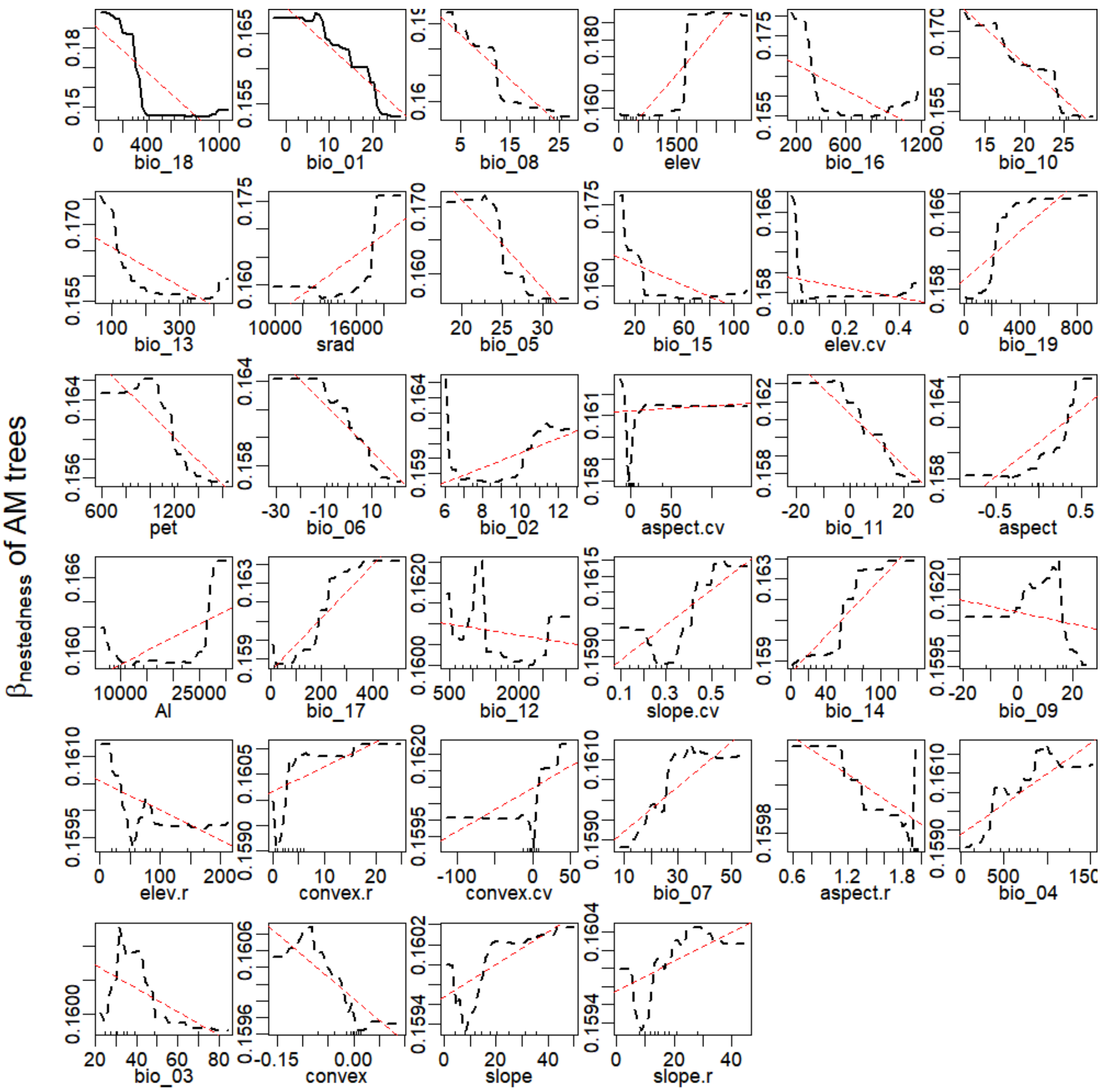

Figure S7.24 Partial dependence of the nestedness component of AM trees on each of 34 environmental variables at the scale of 50 m × 50 m.

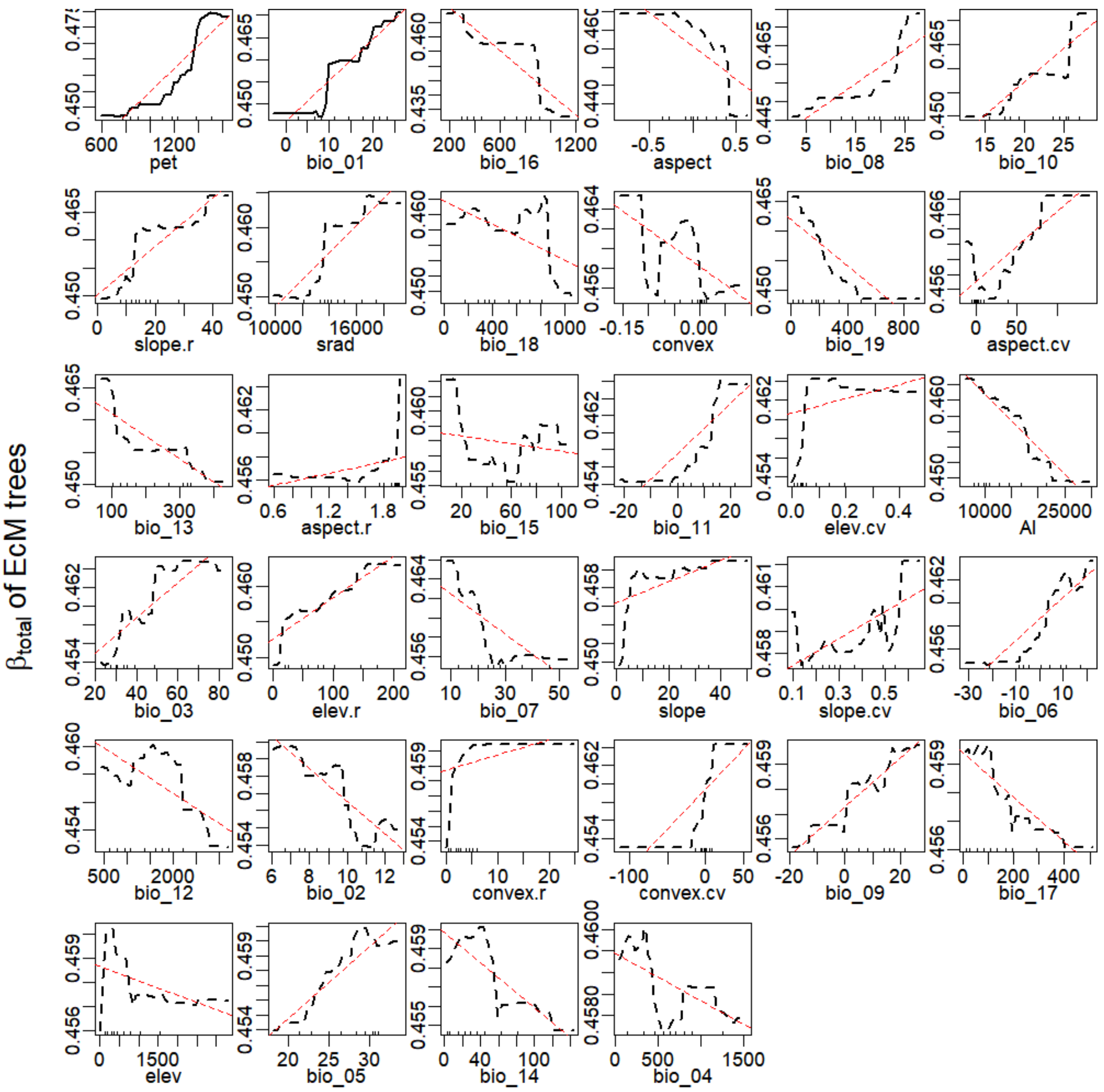

Figure S7.25 Partial dependence of total beta-diversity of EcM trees on each of 34 environmental variables at the scale of 50 m × 50 m.

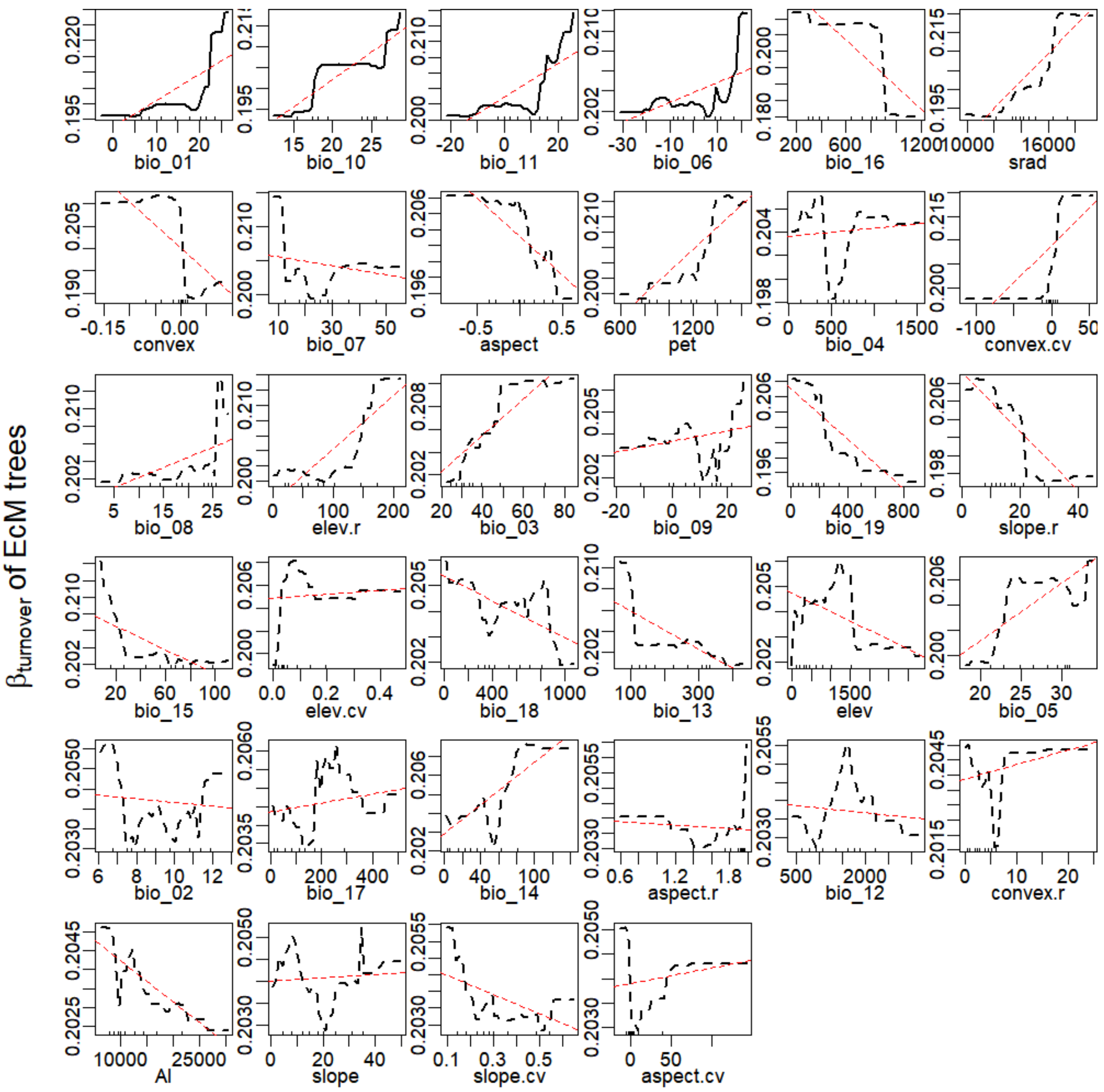

Figure S7.26 Partial dependence of the turnover component of EcM trees on each of 34 environmental variables at the scale of 50 m × 50 m.

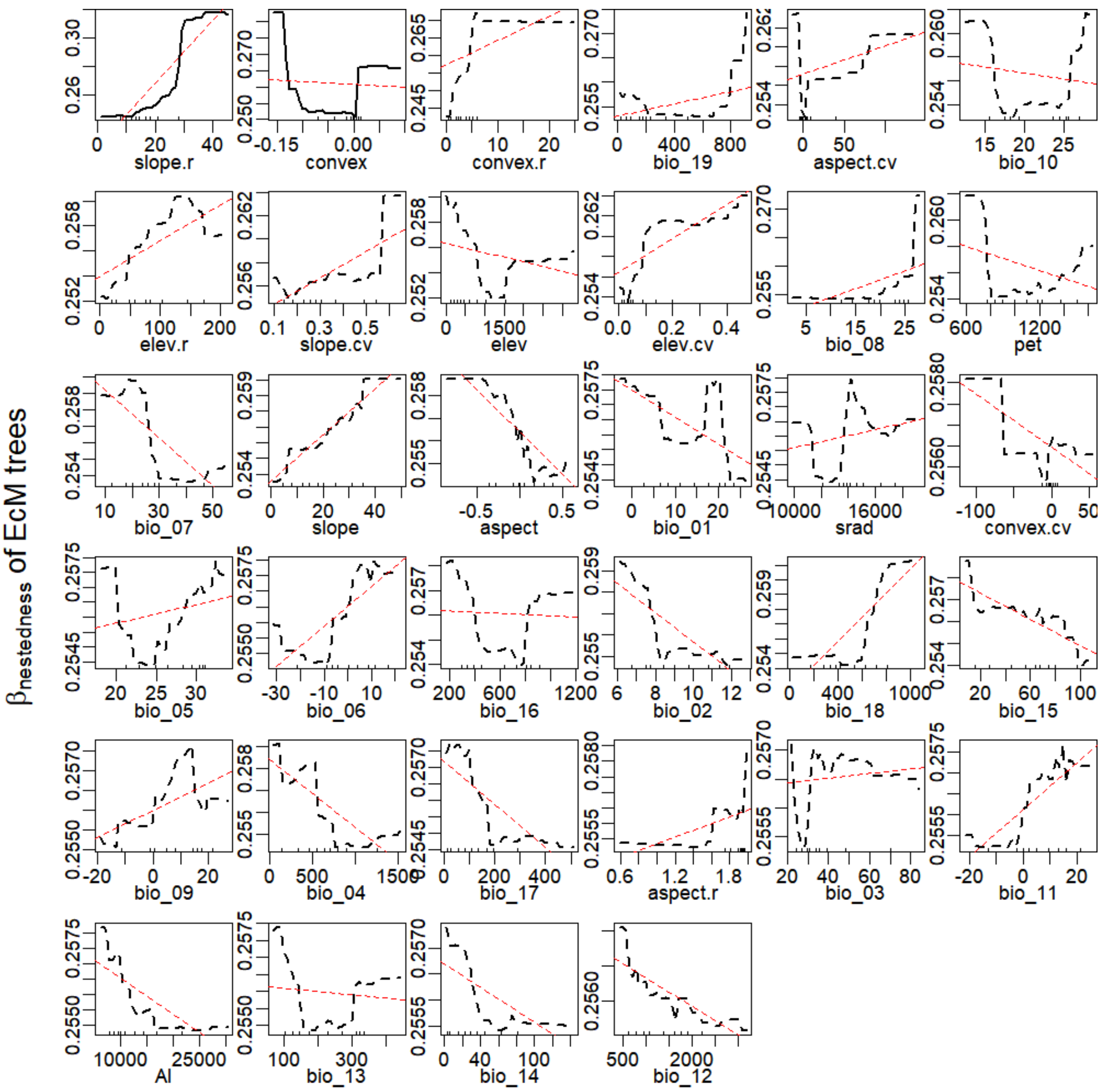

Figure S7.27 Partial dependence of the nestedness component of EcM trees on each of 34 environmental variables at the scale of 50 m × 50 m.

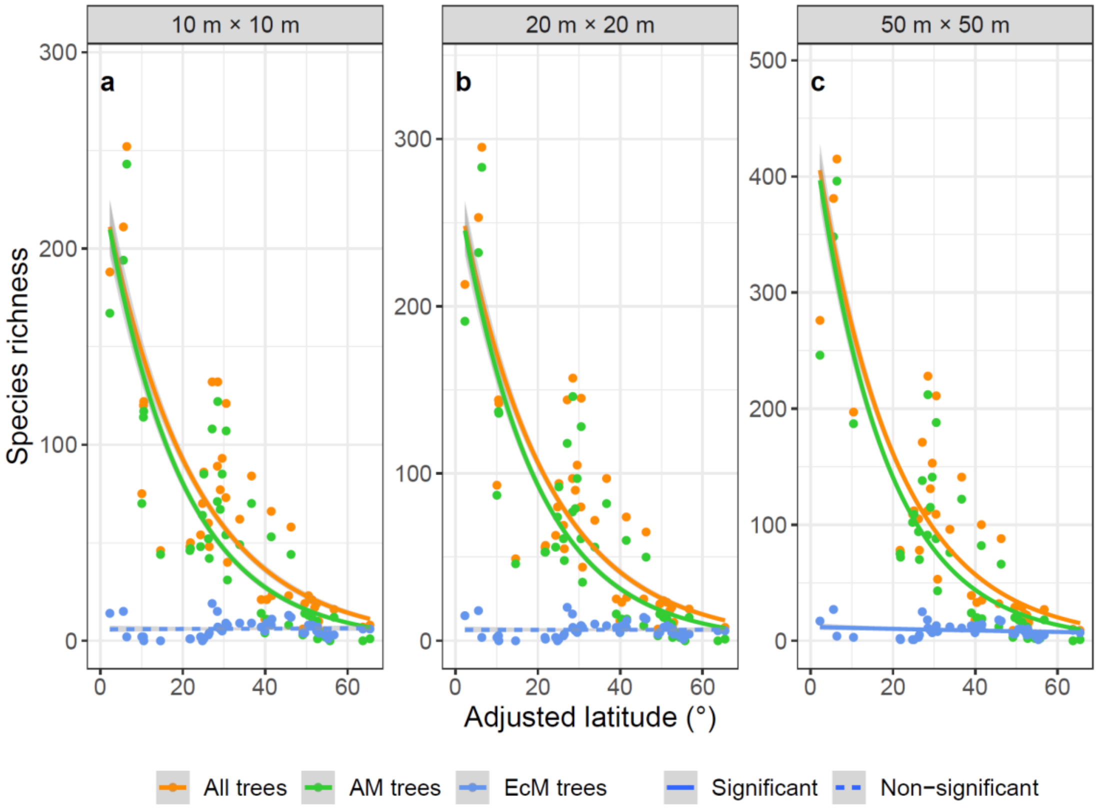

Figure S8 Species richness of all trees, AM trees, and EcM trees at the scale of 10 m × 10 m (a), 20 m × 20 m (b), 50 m × 50 m (c). Orange points represent species richness of all trees and orange lines represent their latitudinal patterns. Green points represent species richness of AM trees and green lines represent their latitudinal patterns. Blue points represent species richness of EcM trees and blue lines represent their latitudinal patterns. Points are the mean values of 200 replicates of number of species of 30, 15, and 15 randomly sampled quadrats of 10 m × 10 m, 20 m × 20 m, and 50 m × 50 m from each forest plot, respectively. Solid lines indicate species richness that varies significantly with latitude while dashed lines indicate non-significant latitudinal patterns fitted using the generalized regression with the Poisson distribution. The error bands (shaded areas) are the 95% confidence intervals of the fitted relationships, with sample size n = 45 at the 10 m × 10 m scale, n = 45 at the 20 m × 20 m scale, and n = 41 at the 50 m × 50 m scale.

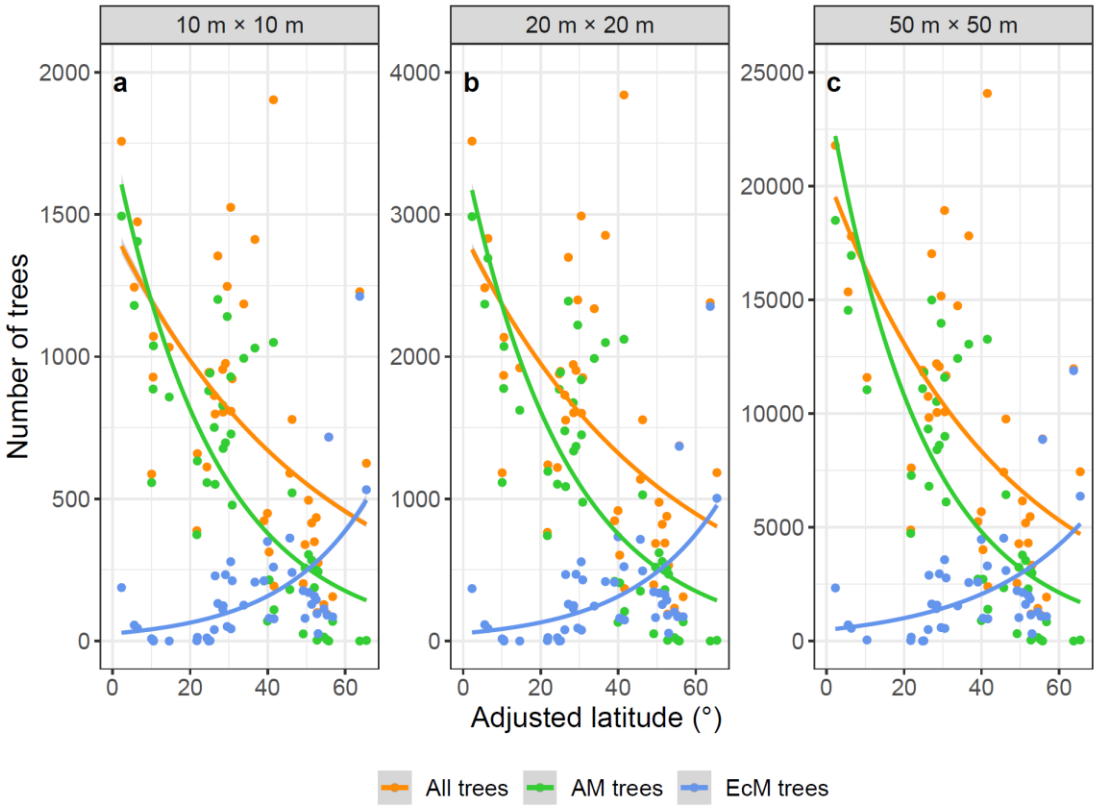

Figure S9 Number of individuals of all trees, AM trees, and EcM trees at the scale of 10 m × 10 m (a), 20 m × 20 m (b), 50 m × 50 m (c). Orange points represent the number of individuals of all trees and orange lines represent their latitudinal patterns. Green points represent number of individuals of AM trees and green lines represent their latitudinal patterns. Blue points represent the number of individuals of EcM trees and blue lines represent their latitudinal patterns. Points are the mean values of 200 replicates of number of trees of 30, 15, and 15 randomly sampled quadrats of 10 m × 10 m, 20 m × 20 m, and 50 m × 50 m from each forest plot, respectively. Solid lines indicate the number of individuals that varies significantly with latitude while dashed lines indicate non-significant latitudinal patterns fitted using the generalized regression with the Poisson distribution. The error bands (shaded areas) are the 95% confidence intervals of the fitted relationships, with sample size n = 45 at the 10 m × 10 m scale, n = 45 at the 20 m × 20 m scale, and n = 41 at the 50 m × 50 m scale.

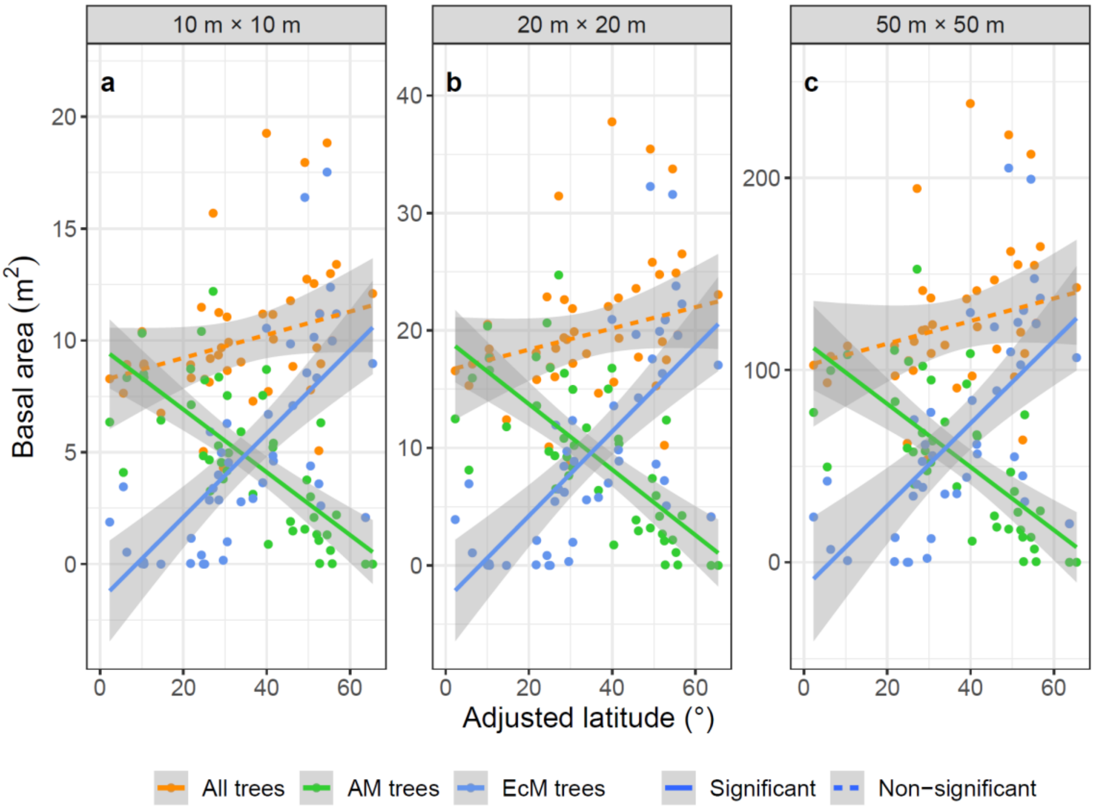

Figure S10 Basal area of all trees, AM trees, and EcM trees at the scale of 10 m × 10 m (a), 20 m × 20 m (b), 50 m × 50 m (c). Orange points represent the basal area of all trees and orange lines represent their latitudinal patterns. Green points represent the basal area of AM trees and green lines represent their latitudinal patterns. Blue points represent basal area of EcM trees and blue lines represent their latitudinal patterns. Points are the mean values of 200 replicates of basal area of 30, 15, and 15 randomly sampled quadrats of 10 m × 10 m, 20 m × 20 m, and 50 m × 50 m from each forest plot, respectively. Solid lines indicate that basal area varies significantly with latitude while dashed lines indicate non-significant latitudinal patterns fitted using the linear regression. The error bands (shaded areas) are the 95% confidence intervals of the fitted relationships, with sample size n = 45 at the 10 m × 10 m scale, n = 45 at the 20 m × 20 m scale, and n = 41 at the 50 m × 50 m scale.

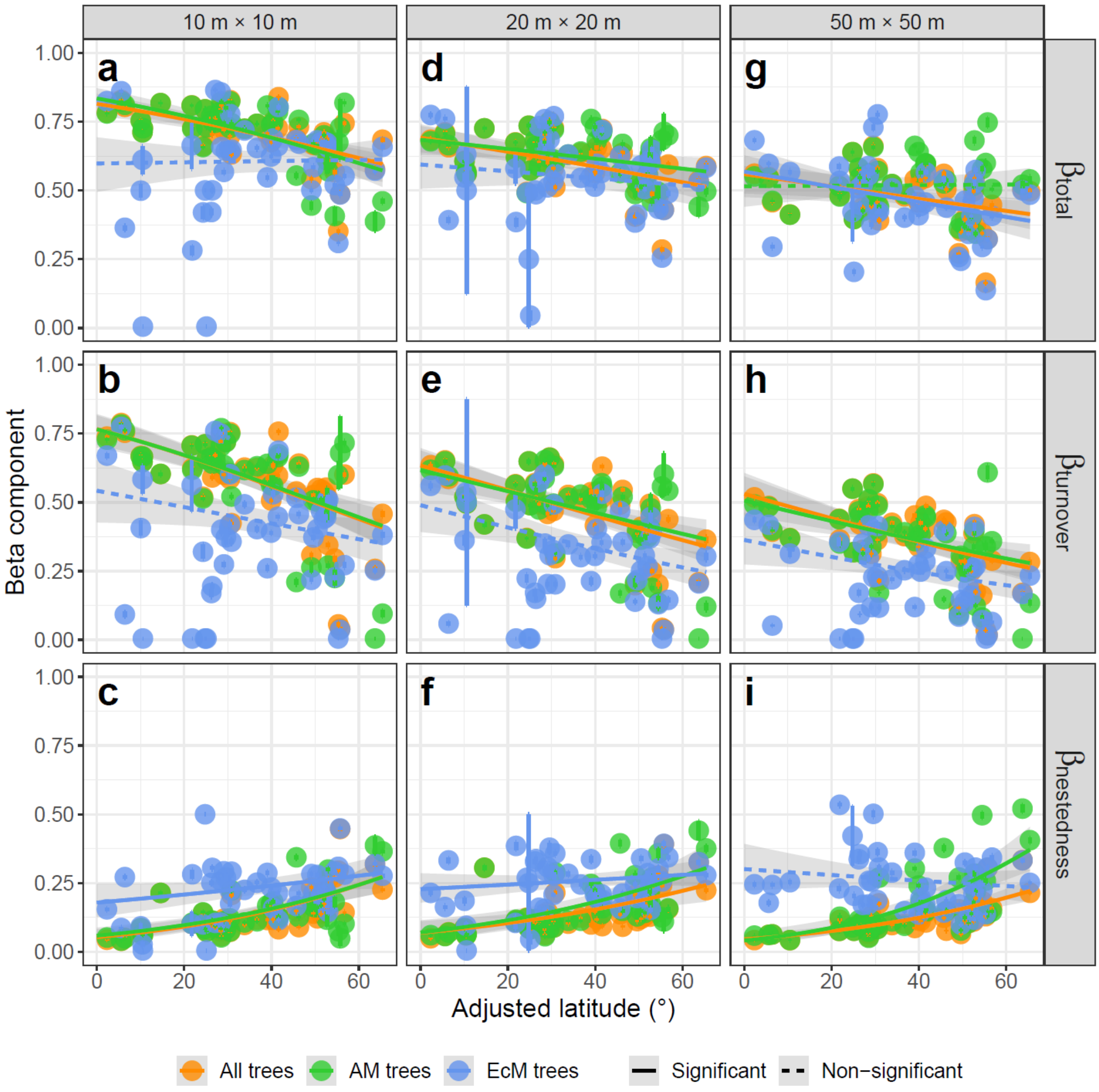

Figure S11 Total beta-diversity, species turnover, and species nestedness of all trees, AM trees, and EcM trees, with AM-EcM trees classified as AM trees and with NM-AM trees classified as AM trees, along latitude at the scale of 10 m × 10 m (a-c), 20 m × 20 m (d-f), and 50 m × 50 m (g-i). Orange points represent beta-diversity and its components of all trees and orange lines represent their latitudinal patterns. Green points represent beta-diversity and its components of AM trees and green lines represent their latitudinal patterns. Blue points represent beta-diversity and its components of EcM trees and blue lines represent their latitudinal patterns. Points are the mean values and the error bars are the 95% confidence intervals, estimated using the non-parametric bootstrapping (n = 200). 200 replicates of average pairwise beta-diversity and its components were calculated based on 30, 15, and 15 randomly sampled quadrats of 10 m × 10 m, 20 m × 20 m, and 50 m × 50 m from each forest

plot, respectively. Solid lines indicate significant relationships with latitude whereas dashed lines indicate non-significant relationships fitted using the beta regression. The error bands (shaded areas) are the 95% confidence intervals of the fitted relationships, with sample size n = 45 for all trees, n = 45 for AM trees, and n = 44 for EcM trees at the 10 m × 10 m scale; with n = 45 for all trees, n = 45 for AM trees, and n = 44 for EcM trees at the 20 m × 20 m scale; and with n = 41 for all trees, n = 41 for AM trees, and n = 41 for EcM trees at the 50 m × 50 m scale.

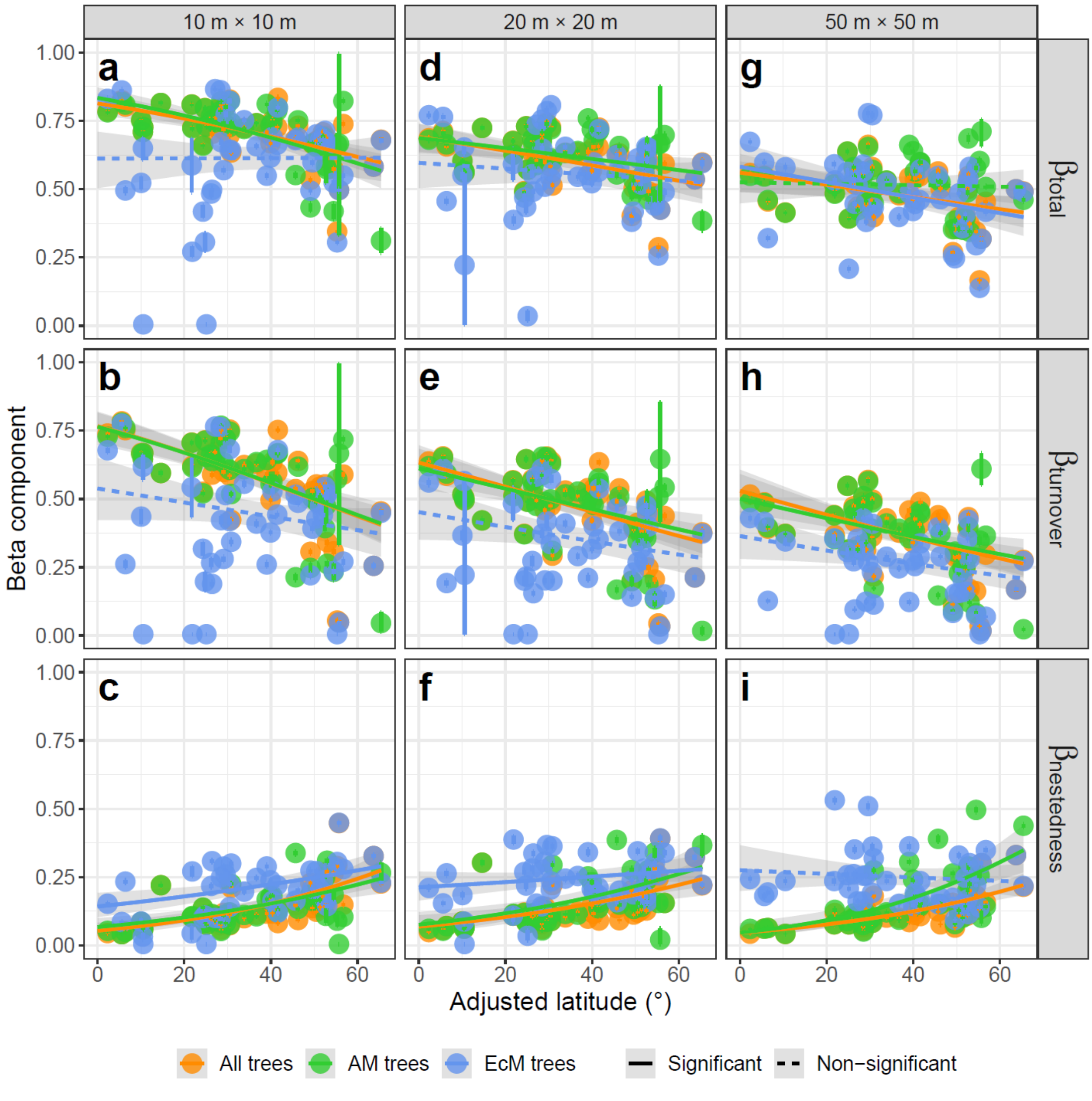

Figure S12 Total beta-diversity, species turnover, and species nestedness of all trees, AM trees, and EcM trees, with AM-EcM trees classified as EcM trees and with NM-AM trees classified as AM trees, along latitude at the scale of 10 m × 10 m (a-c),

20 m × 20 m (d-f), and 50 m × 50 m (g-i). Orange points represent beta-diversity and its components of all trees and orange lines represent their latitudinal patterns. Green points represent beta-diversity and its components of AM trees and green lines represent their latitudinal patterns. Blue points represent beta-diversity and its components of EcM trees and blue lines represent their latitudinal patterns. Points are the mean values and the error bars are the 95% confidence intervals, estimated using non-parametric bootstrapping (n = 200). 200 replicates of average pairwise beta-diversity and its components were calculated based on 30, 15, and 15 randomly sampled quadrats of 10 m × 10 m, 20 m × 20 m, and 50 m × 50 m from each forest plot, respectively. Solid lines indicate significant relationships with latitude whereas dashed lines indicate non-significant relationships fitted using the beta regression. The error bands (shaded areas) are the 95% confidence intervals of the fitted relationships, with sample size n = 45 for all trees, n = 44 for AM trees, and n = 44 for EcM trees at the 10 m × 10 m scale; with n = 45 for all trees, n = 44 for AM trees, and n = 44 for EcM trees at the 20 m × 20 m scale; and with n = 41 for all trees, n = 40 for AM trees, and n = 41 for EcM trees at the 50 m × 50 m scale.

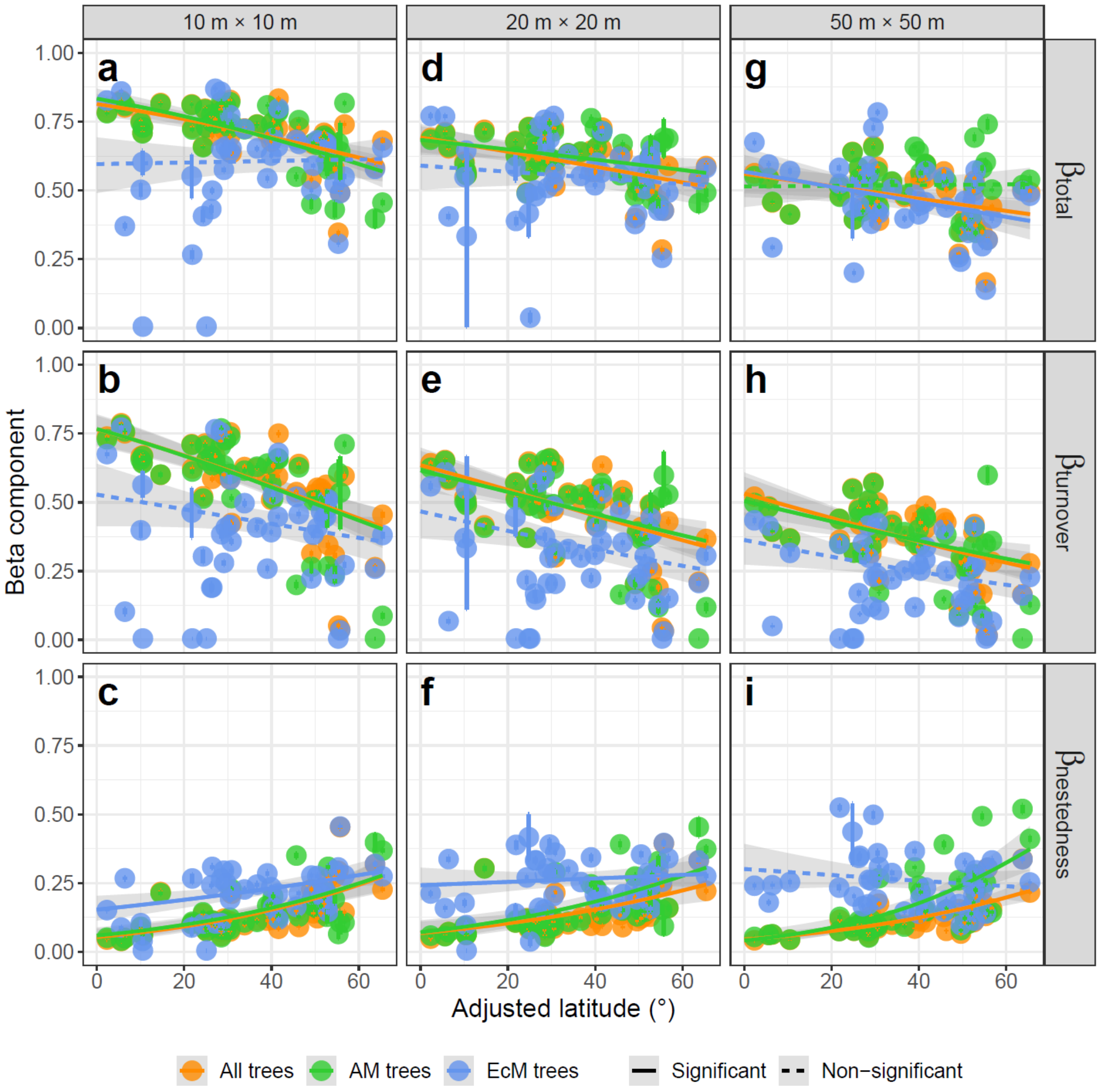

Figure S13 Total beta-diversity, species turnover, and species nestedness of all trees, AM trees, and EcM trees, with AM-EcM trees classified as AM trees and with NM-AM trees classified as NM trees, along latitude at the scale of 10 m × 10 m (a-c), 20 m × 20 m (d-f), and 50 m × 50 m (g-i). Orange points represent beta-diversity and its components of all trees and orange lines represent their latitudinal patterns. Green points represent beta-diversity and its components of AM trees and green lines represent their latitudinal patterns. Blue points represent beta-diversity and its components of EcM trees and blue lines represent their latitudinal patterns. Points are the mean values and the error bars are the 95% confidence intervals, estimated using non-parametric bootstrapping (n = 200). 200 replicates of average pairwise beta-diversity and its components were calculated based on 30, 15, and 15 randomly

sampled quadrats of 10 m × 10 m, 20 m × 20 m, and 50 m × 50 m from each forest plot, respectively. Solid lines indicate significant relationships with latitude whereas dashed lines indicate non-significant relationships fitted using the beta regression. The error bands (shaded areas) are the 95% confidence intervals of the fitted relationships, with sample size n = 45 for all trees, n = 45 for AM trees, and n = 43 for EcM trees at the 10 m × 10 m scale; with n = 45 for all trees, n = 45 for AM trees, and n = 44 for EcM trees at the 20 m × 20 m scale; and with n = 41 for all trees, n = 41 for AM trees, and n = 41 for EcM trees at the 50 m × 50 m scale.

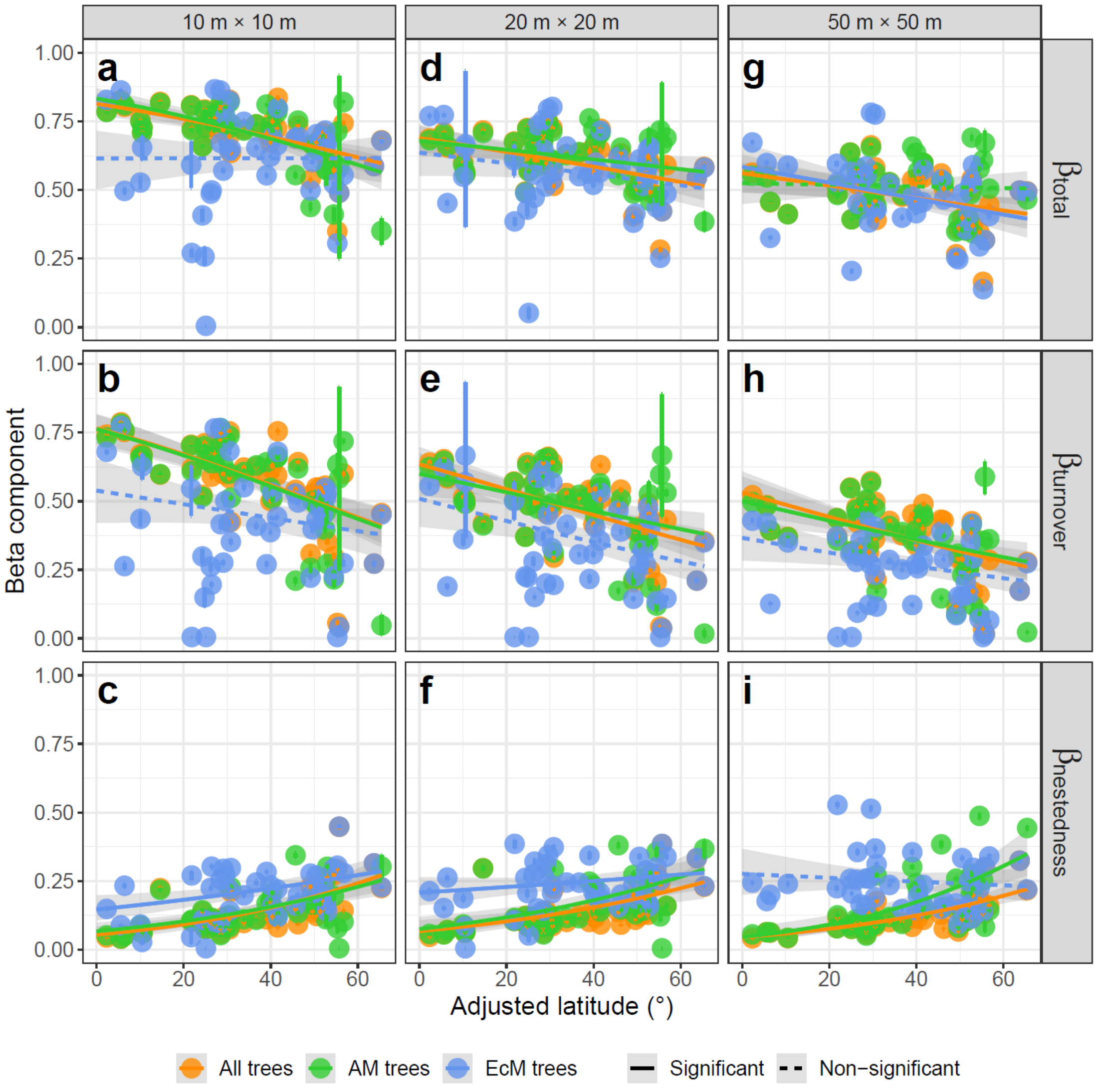

Figure S14 Total beta-diversity, species turnover, and species nestedness of all trees, AM trees, and EcM trees, with AM-EcM trees classified as EcM trees and with

NM-AM trees classified as NM trees, along latitude at the scale of 10 m × 10 m (a-c), 20 m × 20 m (d-f), and 50 m × 50 m (g-i). Orange points represent beta-diversity and its components of all trees and orange lines represent their latitudinal patterns. Green points represent beta-diversity and its components of AM trees and green lines represent their latitudinal patterns. Blue points represent beta-diversity and its components of EcM trees and blue lines represent their latitudinal patterns. Points are the mean values and the error bars are the 95% confidence intervals, estimated using non-parametric bootstrapping (n = 200). 200 replicates of average pairwise beta-diversity and its components were calculated based on 30, 15, and 15 randomly sampled quadrats of 10 m × 10 m, 20 m × 20 m, and 50 m × 50 m from each forest plot, respectively. Solid lines indicate significant relationships with latitude whereas dashed lines indicate non-significant relationships fitted using the beta regression. The error bands (shaded areas) are the 95% confidence intervals of the fitted relationships, with sample size n = 45 for all trees, n = 44 for AM trees, and n = 43 for EcM trees at the 10 m × 10 m scale; with n = 45 for all trees, n = 44 for AM trees, and n = 44 for EcM trees at the 20 m × 20 m scale; and with n = 41 for all trees, n = 40 for AM trees, and n = 41 for EcM trees at the 50 m × 50 m scale.

Supplementary Table S1 Summary of study site characteristics with meta data

Study site	Site_code	Plot size (ha)	Ecological zone
Badagongshan	BDG	25	Subtropical humid forest
Baishanzu	BSZ	25	Subtropical humid forest
Barro Colorado Island	BCI	50	Tropical rainforest
Changbaishan	CBS	25	Temperate continental forest
Chebaling	CBL	20	Subtropical humid forest
Cocoli	Cocoli	4	Tropical moist deciduous forest
Danum Valley	Danum	50	Tropical rainforest
Dinghushan	DHS	20	Subtropical humid forest
Donglingshan	DLS	20	Temperate mountain system
Dongyuan	DY	10	Subtropical humid forest
Fenglin	FL	30	Temperate continental forest
Fushan	FS	25	Subtropical humid forest
Gutianshan	GTS	24	Subtropical humid forest
Harvard Forest	Harvard	35	Temperate mountain system
Heishiding	HSD	50	Subtropical humid forest
Ilha do Cardoso	Ilha	10.2	Tropical rainforest
Jianfengling	JFL	60	Tropical mountain system
Liangshui	LS	9	Temperate continental forest
Luquillo	Luquillo	16	Tropical rainforest
Muling	ML	25	Temperate continental forest
Mulun	Mulun	25	Karst mixed evergreen and deciduous broadleaved forest
Nabanhe	NBH	20	Tropical seasonal rainforest
Nanjenshan	NJS	2.1	Subtropical humid forest
Ngardok	Ngardok	4	Tropical lowland rainforest
Ngel Nyaki	Ngel	20.28	Tropical rainforest
Nonggang	NG	15	Subtropical humid forest
Qinling	QL	25	Deciduous broadleaved forest
Rabi	Rabi	25	Tropical rainforest
Santa Cruz	Santa	16	Subtropical dry forest
SCBI	SCBI	25.6	Temperate mountain system
Scotty Creek	Scotty	10	Subarctic boreal peatland
SERC	SERC	16	Temperate mountain system
Sherman	Sherman	6	Tropical moist forest
Speulderbos	Speulderbos	27	Temperate oceanic forest
Tiantongshan	TTS	20	Subtropical humid forest
Tyson	Tyson	20	Temperate continental forest
Uholka	Uholka	10	Primeval beech forest
Utah	Utah	9	Temperate continental forest
Wanang	Wanang	50	Tropical rainforest
Wind River	WindRive	25.6	Temperate continental forest
Wytham Woods	Wytham	18	Temperate oceanic forest
Xishuangbanna	XSBN	20	Tropical mountain system
Yosemite	Yosemite	25.6	Temperate continental forest
Yulongxueshan	YLXS	25	Subtropical humid forest
Žofín	Zofin	25	Temperate mountain system

**Latitude**	**Longitude**	**Elevation**	**Adusted latitude**	**N.total**	**N.AM**	**N.EcM**	**S.total**	**S.AM**
29.4600	110.5200	803.2	36.6960	118639	86939	17377	182	157
27.7610	119.1980	1523.3	41.4847	160762	88812	22065	122	101
9.1543	-79.8461	144.4	10.4550	155107	147816	644	243	231
42.3833	128.0830	803.2	49.6193	28594	21684	6848	34	23
24.7100	114.2500	488.5	29.1106	64251	45924	15815	165	145
8.9877	-79.6166	120.0	10.0688	7881	7441	106	152	143
5.1019	117.6880	54.3	5.5911	204936	194668	9603	602	543
23.1695	112.5110	339.2	26.2254	57929	50310	2696	145	132
39.9566	115.4250	1394.6	52.5207	29181	16787	9817	23	12
23.7333	115.0667	300.5	26.4401	26364	18337	7733	95	86
48.0800	129.1200	438.3	52.0283	34731	18366	16248	26	14
24.7614	121.5550	675.8	30.8493	76594	39960	18084	66	55
29.2500	118.1170	134.4	30.4611	121666	74537	22796	133	107
42.5388	-72.1755	352.4	45.7136	69885	23712	42129	43	19
23.2700	111.5300	569.6	28.4014	161740	139404	19215	128	103
-25.0955	-47.9573	2.5	25.1177	28509	28398	30	120	117
18.7281	108.9050	933.9	27.1414	270749	240245	26178	190	152
47.1800	128.8800	463.2	51.3528	12422	8462	3873	31	16
18.3262	-65.8160	378.2	21.7336	20754	20126	90	99	95
43.9500	130.0700	729.8	50.5251	41148	25371	14470	35	20
25.1310	108.0094	491.8	29.5616	101688	93503	4204	200	183
22.2460	100.6023	920.5	30.5386	53449	48109	2858	260	233
22.0817	120.8475	249.5	24.3294	4014	3610	76	81	71
7.5101	134.6069	788.2	14.6114	12679	10570	0	58	55
7.0680	11.0566	1642.4	21.8648	38491	36882	853	93	87
22.4333	106.9500	261.6	24.7904	47470	44323	7	128	119
33.5421	108.3774	1414.2	46.2828	64879	42645	20741	104	80
-1.9246	9.8800	42.4	2.3069	145654	123915	15543	337	302
37.0124	-122.0750	324.9	39.9393	13599	2109	10859	21	10
38.8935	-78.1454	302.1	41.6154	15351	8933	6237	44	24
61.3000	-121.3000	269.5	63.7279	38050	0	37669	11	0
38.8890	-76.5590	17.9	39.0504	22423	11449	10913	51	30
9.2815	-79.9740	140.0	10.5428	21377	20717	4	211	199
52.2530	5.7020	52.0	52.7215	8615	304	8311	13	2
29.8116	121.7830	445.9	33.8283	78524	66192	8598	112	89
38.5178	-90.5575	206.0	40.3735	21210	14536	5447	42	26
48.1820	23.6350	791.2	55.3101	3020	136	2884	6	5
37.6602	-112.8550	3083.6	65.4405	17862	105	15226	10	2
-5.2310	145.1820	131.3	6.4139	235997	224979	7454	553	525
45.8197	-121.9560	369.9	49.1517	17113	2223	14890	11	4
51.7743	-1.3379	135.5	52.9950	16080	14448	1603	23	11
21.6117	101.5740	765.0	28.5035	53653	44796	8516	276	255
37.7662	-119.8190	1857.0	54.4955	9867	1182	8684	12	5
27.1400	100.2250	3285.7	56.7412	12954	5673	7170	34	21
48.6638	14.7073	785.1	55.7369	58409	23	58303	10	3

S.EcM	BA.total	BA.AM	BA.EcM	bio_01	bio_02	bio_03	bio_04	bio_05
16	605.52	261.67	239.82	15.74	8.27	27.13	801.91	30.40
13	936.28	437.36	407.48	10.30	7.73	30.06	658.89	23.50
3	1496.17	1433.69	12.37	26.05	6.12	74.59	67.05	30.40
8	1075.15	310.86	728.64	3.25	11.46	24.69	1253.41	25.10
11	647.38	308.81	328.88	17.85	7.72	29.94	662.20	30.40
3	135.04	134.06	0.22	26.60	6.51	76.57	64.79	31.10
48	1244.71	661.91	561.07	26.39	9.61	84.28	44.47	32.10
4	534.90	308.93	183.69	20.20	7.63	34.08	557.32	30.80
6	338.89	69.90	239.79	6.68	10.92	27.02	1085.50	26.30
6	304.79	109.02	195.60	19.93	7.86	34.77	555.59	31.10
9	956.94	132.23	823.24	0.86	12.81	23.16	1529.38	26.70
8	823.65	419.20	370.30	18.60	6.08	32.31	459.94	27.30
13	879.98	333.07	498.84	15.64	7.35	24.50	823.58	30.90
20	1368.71	226.95	1138.93	6.89	11.66	30.52	968.15	24.80
20	1602.86	894.68	696.83	22.21	8.49	34.95	589.04	33.80
1	251.82	250.81	0.02	21.88	7.45	47.45	285.48	29.90
28	3109.58	2445.89	641.73	20.68	6.69	40.56	362.48	28.10
10	372.60	62.35	301.24	1.56	12.32	23.34	1461.60	26.40
2	476.15	466.48	1.49	23.23	8.75	70.00	146.63	29.40
11	642.73	246.11	363.87	2.38	11.91	25.18	1285.14	25.00
9	355.36	316.51	14.09	17.75	7.61	28.71	691.47	30.40
14	573.87	500.57	64.95	20.25	9.13	48.28	350.83	28.50
3	74.85	67.61	2.74	23.27	4.93	39.15	286.43	28.80
0	80.80	76.92	0.00	27.32	7.17	86.45	30.78	31.50
1	487.98	421.44	63.78	20.45	9.71	72.45	128.95	26.50
1	249.18	239.38	0.01	22.20	8.02	35.79	532.55	32.40
19	740.03	121.03	596.99	8.15	9.30	30.69	768.21	22.70
18	686.48	522.58	157.12	25.58	6.73	61.21	148.53	30.40
8	570.98	259.21	311.32	13.81	11.78	57.76	330.54	24.00
19	780.93	416.48	361.33	11.71	11.88	33.19	864.90	29.10
10	63.46	0.00	63.35	-2.86	12.31	23.09	1529.25	21.90
17	583.57	396.20	187.17	13.34	11.37	31.84	876.35	30.70
2	177.14	164.80	0.01	26.13	6.20	77.50	54.79	30.30
11	942.18	2.26	939.92	9.44	7.70	34.22	557.77	20.10
13	603.38	389.37	189.93	14.98	6.32	22.19	805.89	29.50
15	523.45	59.61	454.84	12.60	11.11	29.08	966.07	30.70
1	414.33	18.88	395.45	8.07	8.58	28.97	783.27	21.50
7	341.80	0.02	253.05	2.96	10.23	35.05	719.41	18.10
6	1426.97	1329.74	88.24	24.88	10.59	84.06	45.15	31.00
7	1536.16	139.73	1396.43	9.21	10.74	38.23	624.99	22.00
11	522.19	369.64	150.87	9.65	7.38	37.82	456.31	19.20
12	754.68	547.06	206.26	21.34	8.88	49.35	340.00	29.30
6	1450.07	98.65	1351.42	8.30	11.38	42.32	592.45	22.90
6	1096.91	177.14	919.10	6.65	9.88	38.76	543.25	19.10
4	826.64	1.85	824.66	6.15	7.49	29.97	674.57	18.10

bio_06	bio_07	bio_08	bio_09	bio_10	bio_11	bio_12	bio_13	bio_14	bio_15
-0.10	30.50	23.40	5.60	25.40	5.60	1379	226	28	58.41
-2.20	25.70	13.60	3.67	18.10	1.93	2115	342	45	55.02
22.20	8.20	25.68	26.20	27.00	25.35	2210	330	11	64.48
-21.30	46.40	18.15	-13.07	18.15	-13.07	680	163	4	99.22
4.60	25.80	21.57	10.73	25.32	9.10	1721	302	36	63.34
22.60	8.50	26.00	26.82	27.52	25.93	2250	339	13	69.44
20.70	11.40	25.87	26.92	26.92	25.82	2346	259	143	15.92
8.40	22.40	25.47	14.45	26.23	12.77	1863	322	37	67.24
-14.10	40.40	19.68	-7.17	19.68	-7.17	504	145	3	109.73
8.50	22.60	23.07	14.13	26.05	12.60	1827	311	32	64.02
-28.60	55.30	18.75	-19.10	18.75	-19.10	623	154	4	105.58
8.50	18.80	23.75	12.73	23.82	12.73	2982	441	117	45.58
0.90	30.00	19.47	7.23	25.45	5.22	1788	313	45	56.58
-13.40	38.20	18.45	-4.58	18.45	-5.28	1117	103	80	7.71
9.50	24.30	25.58	15.98	28.65	14.38	1676	301	35	68.67
14.20	15.70	25.40	18.45	25.40	18.45	2244	332	80	45.13
11.60	16.50	23.10	15.72	24.33	15.72	1523	286	23	76.60
-26.40	52.80	18.83	-17.45	18.83	-17.45	622	160	3	108.68
16.90	12.50	24.57	21.35	24.82	21.35	2110	228	89	27.37
-22.30	47.30	17.68	-14.20	17.68	-14.20	599	134	2	97.56
3.90	26.50	24.50	8.83	25.80	8.83	1379	247	29	68.57
9.60	18.90	23.50	16.88	23.50	15.23	1583	317	18	83.33
16.20	12.60	26.20	19.68	26.20	19.33	2711	594	43	90.19
23.20	8.30	27.35	27.28	27.72	26.95	3407	405	173	25.75
13.10	13.40	18.98	20.82	22.27	18.98	1748	316	4	81.72
10.00	22.40	27.90	16.32	27.90	14.98	1357	236	23	74.31
-7.60	30.30	16.53	-1.63	17.45	-1.63	810	143	7	71.35
19.40	11.00	26.28	23.40	27.03	23.40	1830	353	2	74.43
3.60	20.40	10.45	17.52	17.98	9.97	864	180	3	92.97
-6.70	35.80	20.17	0.82	22.20	0.82	1026	101	68	13.31
-31.40	53.30	15.08	-11.17	15.08	-21.73	419	74	18	54.90
-5.00	35.70	21.83	2.37	24.00	2.37	1101	108	72	12.41
22.30	8.00	25.70	26.13	26.90	25.53	2370	444	22	68.52
-2.40	22.50	6.33	5.50	16.42	2.75	867	85	53	14.30
1.00	28.50	24.52	7.33	24.62	4.93	1358	187	45	43.36
-7.50	38.20	12.53	0.12	24.13	0.12	981	107	51	19.41
-8.10	29.60	17.28	-0.52	17.28	-1.95	1050	127	62	25.64
-11.10	29.20	12.40	3.93	12.40	-4.83	568	67	24	24.41
18.40	12.60	25.27	24.22	25.32	24.22	3172	398	123	40.94
-6.10	28.10	2.18	16.82	16.83	1.63	2008	330	26	65.90
-0.30	19.50	5.45	5.95	15.57	4.57	711	72	45	14.05
11.30	18.00	24.32	17.87	24.62	16.55	1695	323	17	77.86
-4.00	26.90	2.40	15.75	16.33	2.33	970	177	8	79.90
-6.40	25.50	13.02	0.35	13.02	-0.15	808	145	12	65.94
-6.90	25.00	14.48	-0.90	14.48	-1.90	896	120	51	29.39

**bio_16**	**bio_17**	**bio_18**	**bio_19**	**srad**	**AI**	**pet**
609	101	571	101	13325.92	12864	1096
852	180	808	218	14067.75	24758	868
869	65	396	648	19277.17	18723	1394
428	19	428	19	13505.75	8664	801
810	133	623	189	14094.00	14414	1221
980	59	415	798	19436.67	13428	1502
703	510	510	692	17415.17	16601	1483
841	121	790	157	13699.08	14404	1201
336	11	336	11	15591.33	6171	849
809	130	732	180	14156.17	15743	1217
403	15	403	15	13385.17	8627	758
1185	392	1044	392	14742.42	30265	1056
808	174	609	224	14595.33	17554	1059
295	255	295	261	13032.33	12497	921
772	118	706	153	14587.67	10674	1349
925	287	925	287	13252.00	18278	1336
779	75	486	75	17107.83	13417	1229
411	13	411	13	13601.00	8372	774
669	326	569	326	18758.50	21401	1420
364	10	364	10	13611.58	7849	772
666	103	642	103	13642.42	11856	1196
837	58	837	69	16724.92	9687	1505
1539	161	1539	166	16122.75	23829	1264
1143	570	845	741	16719.33	24215	1447
909	25	220	909	15560.17	11022	1555
694	89	694	90	14865.08	9992	1370
391	27	347	27	13709.83	10450	865
773	22	681	22	12139.33	12034	1637
468	11	17	463	16690.00	7952	1133
296	208	287	208	13990.25	9091	1112
194	55	194	57	10195.25	6337	587
309	243	305	243	14151.67	9744	1095
1000	84	292	635	19254.00	23841	1338
246	184	219	215	9665.00	11896	675
476	165	470	174	14308.17	16146	916
292	181	263	181	14600.67	8523	1172
354	195	354	225	12012.50	9612	801
169	102	169	144	17562.25	7366	843
1151	393	1072	393	16057.42	24047	1616
952	135	146	894	12799.50	28142	910
209	157	163	190	9835.50	9689	677
889	73	751	85	16309.58	10483	1531
481	34	43	479	17744.67	10187	1015
383	50	383	55	14083.33	9599	973
323	170	323	174	10813.33	12991	722

Meta data

**Header**	**Explanation**
N.total	Number of individuals of all trees
N.AM	Number of individuals of AM trees
N.EcM	Number of individuals of EcM trees
S.total	Number of species of all trees
S.AM	Number of species of AM trees
S.EcM	Number of species of EcM trees
BA.total	Basal area of all trees
BA.AM	Basal area of AM trees
BA.EcM	Basal area of EcM trees
bio_01	Annual Mean Temperature
bio_02	Mean Diurnal Range (Mean of monthly (max temp - min temp))
bio_03	Isothermality (BIO2/BIO7) (×100)
bio_04	Temperature Seasonality (standard deviation ×100)
bio_05	Max Temperature of Warmest Month
bio_06	Min Temperature of Coldest Month
bio_07	Temperature Annual Range (BIO5-BIO6)
bio_08	Mean Temperature of Wettest Quarter
bio_09	Mean Temperature of Driest Quarter
bio_10	Mean Temperature of Warmest Quarter
bio_11	Mean Temperature of Coldest Quarter
bio_12	Annual Precipitation
bio_13	Precipitation of Wettest Month
bio_14	Precipitation of Driest Month
bio_15	Precipitation Seasonality (Coefficient of Variation)
bio_16	Precipitation of Wettest Quarter
bio_17	Precipitation of Driest Quarter
bio_18	Precipitation of Warmest Quarter
bio_19	Precipitation of Coldest Quarter
srad	Solar Radiation
AI	Aridity Index
pet	Potential Evapotranspiration

Supplementary References

The funding information for each permanent forest plot.

1. Badagongshan

The Badagongshan Forest Dynamics Plot is supported by Chinese Forest Biodiversity Monitoring Network. Funding was provided by Biodiversity Council, CAS, and the National Natural Science Foundation of China (31270562 and 31200329).

Xu Y, J Zhang, SB Franklin, J Liang, P Ding, Y Luo, Z Lu, D Bao and M Jiang. 2015. Improving allometry models to estimate the above- and belowground biomass of subtropical forest, China. Ecosphere , 6(12):289. http://dx.doi.org/10.1890/ES15-00198.1

Xiujuan Qiao, Qianxi Li, Qinghu Jiang, Junmeng Lu, Scott Franklin, Zhiyao Tang,Qinggang Wang, Jiaxin Zhang, Zhijun Lu, Dachuan Bao, Yili Guo, Haibo Liu,Yaozhan Xu & Mingxi Jiang. 2015. Beta diversity determinants in Badagongshan, a subtropical forest in central China. Scientific Reports, 5:17043. DOI: 10.1038/srep17043.

Junmeng Lu, Daniel J. Johnson, Xiujuan Qiao, Zhijun Lu, Qinggang Wang, and Mingxi Jiang. 2015. Density dependence and habitat preference shape seedling survival in a subtropical forest in central China. Journal of Plant Ecology, 8 (6): 568-577. DOI:10.1093/jpe/rtv006

Yaozhan Xu, Scott B. Franklin, Qinggang Wang, Zheng Shi, Yiqi Luo, Zhijun Lu, Jiaxin Zhang, Xiujuan Qiao, Mingxi Jiang. 2015. Topographic and biotic factors determine forest biomass spatial distribution in a subtropical mountain moist forest. Forest Ecology and Management, 357:95-103.

Yili Guo, Zhijun Lu, Qinggang Wang, Junmeng Lu, Yaozhan Xu, Hongjie Meng, Haibo Liu, Jiaxin Zhang, Dachuan Bao, Xiujuan Qiao, Handong Huang and Mingxi Jiang. 2015. Detecting density dependence from spatial patterns in a heterogeneous subtropical forest of central China. Canadian Journal of Forest Research, 45: 710–720.

Qinggang Wang, Yaozhan Xu, Zhijun Lu, Dachuan Bao, Yili Guo, Junmeng Lu, Kuihan Zhang, Haibo Liu, Hongjie Meng, Xiujuan Qiao, Handong Huang and Mingxi Jiang. 2014. Disentangling the effects of topography and space on the distributions of dominant species in a subtropical forest. Chin. Sci. Bull., 59(35):5113-5122.

Qinggang Wang, Dachuan Bao, Yili Guo, Junmeng Lu, Zhijun Lu, Yaozhan Xu, Kuihan Zhang, Haibo Liu, Hongjie Meng, Mingxi Jiang, Xiujuan Qiao and Handong Huang. 2014. Species associations in a species-rich subtropical forest were not well-explained by stochastic geometry of biodiversity. PLoS ONE, 9(5): e97300.

Yili Guo, Junmeng Lu, Scott B. Franklin, Qinggang Wang, Yaozhan Xu, Kuihan Zhang, Dachuan Bao, Xiujuan Qiao, Handong Huang，Zhijun Lu and Mingxi Jiang. 2013. Spatial distribution of tree species in a species-rich subtropical mountain forest in central China. Canadian Journal of Forest Research. 43: 826–835.

2. Baishanzu

The Baishanzu Forest Dynamics Plot has been supported by East China Normal University, Sun Yat-sen University, Zhejiang University, Wenzhou University, Lishui University, The BSZ Nature Reserve Management Department.

3. Barro Colorado Island

Condit R., Perez, R., Aguilar, S., Lao, S., Foster, R., Hubbell, S.P. 2019. Complete data from the Barro Colorado 50-ha plot: 423617 trees, 35 years, 2019 version. https://doi.org/10.15146/5xcp-0d46.

Condit, Richard et al. (2019), BCI 50-ha Plot Taxonomy, v4, DataONE, Dataset, https://doi.org/10.15146/R3FH61

Condit, Richard; Pérez, Rolando; Aguilar, Salomón; Lao, Suzanne (2019), Census data from 65 tree plots in Panama, 1994-2015, DataONE, Dataset, https://doi.org/10.15146/mdpr-pm59.

Croat, T.R. 1978. Flora of Barro Colorado Island. Stanford University.

Hubbell, S. P. and Foster, R. B. 1983. Diversity of canopy trees in a neotropical forest and implications for conservation. Pp. 25-41 in Tropical Rain Forest: Ecology and Management, Whitmore, T., Chadwick, A., and Sutton, A. (Eds.). The British Ecological Society.

Hubbell, S. P., Condit, R., and Foster, R. B. 1990. Presence and absence of density dependence in a Neotropical tree community. Philosophical Transactions of the Royal Society of London. Series B: Biological Sciences Pages 269–281.

Condit, R., Hubbell, S. P., and Foster, R. B. 1996. Changes in tree species abundance in a neotropical forest: impact of climate change. Journal of Tropical Ecology 12:231–256.

Condit, R., Aguilar, S., Hernandez, A., Pérez, R., Lao, S., Angehr, G., Hubbell, S., and Foster, R. 2004. Tropical forest dynamics across a rainfall gradient and the impact of an El Niño dry season. Journal of Tropical Ecology 20:51–72.

Condit, R., Chisholm, R. A., and Hubbell, S. P. 2012. Thirty years of forest census at Barro Colorado and the importance of immigration in maintaining diversity. PLoS ONE 7:e49826.

Condit, R., Pérez, R., Aguilar, S., Lao, S., and Hubbell, S. P. 2017. Demographic trends and climate over 35 years in the Barro Colorado 50 ha plot. Forest Ecosystems 4:1–13.

4. Changbaishan

The Changbaishan plot was supported by the National Natural Science Foundation of China (31961133027 and 31730015), Strategic Priority Research Program of the Chinese Academy of Sciences (XDB 31030000), Key Research Program of Frontier Sciences (ZDBS-LY-DQC019) of the Chinese Academy of Sciences.

5. Chebaling

The Chebaling 20-ha forest plot was funded by the National Natural Science Foundation of China (31925027, 31622014 and 31570426 to CC) and Guangdong Chebaling National Nature Reserve.

6. Cocoli
The Cocoli Forest Dynamics Plot has been supported by Smithsonian Tropical Research Institute.

7. Danum Valley
The Danum plot is a core project of the Southeast Asia Rain Forest Research Partnership (SEARRP). We thank SEARRP partners especially Yayasan Sabah for their support, and HSBC Malaysia and the University of Zurich for funding. We are grateful to the research assistants who are conducting the census, in particular the team leader Alex Karolus, and to Mike Bernados and Bill McDonald for species identifications. We thank Stuart Davies and Shameema Esufali for advice and training

8. Dinghushan
Strategic Priority Research Program of the Chinese Academy of Sciences (XDB31030000), National Natural Science Foundation of China (NO. 31300455, 31570527, 41371078), Chinese Forest Biodiversity Monitoring Network and US National Science Foundation grant DEB-1046113.

9. Donglingshan
National Natural Science Foundation of China (NO. 31570630)

10. Dongyuan
NA

11. Fenglin
NA

12. Fushan
Taiwan Forestry Bureau, Taiwan Forestry Research Institute, National Taiwan University (Institute of Ecology and Evolutionary Biology), and the Center for Tropical Forest Science of the Smithsonian Tropical Research Institute.

13. Gutianshan
We thank Drs. Mingjian Yu from Zhejiang University, Jianhua Chen for their contributions to the establishment and census of the 24-ha permanent forest plot. We gratefully acknowledge support from the Administration Bureau of the Gutianshan National Nature Reserve.

14. Harvard Forest
Funding for the Harvard ForestGEO Forest Dynamics plot was provided by the Center for

Tropical Forest Science and Smithsonian Institute's Forest Global Earth Observatory (ForestGEO), the National Science Foundation's LTER program (DEB 06-20443 and DEB 12-37491) and Harvard University. Thanks to many field technicians who helped census the plot and Jason Aylward for field supervision, data screening and database management.

Orwig D, Foster D, Ellison A. 2015. Harvard Forest CTFS-ForestGEO Mapped Forest Plot since 2014. Harvard Forest Data Archive: HF253.
https://harvardforest1.fas.harvard.edu/exist/apps/datasets/showData.html?id=hf253

15. Heishiding

The Heishiding 50-ha forest plot was funded by the National Natural Science Foundation of China (31925027, 31622014 and 31570426 to CC) and Sun Yat-Sen University.

16. Ilha do Cardoso

Alexandre Adalardo de Oliveira receives a fellowship from Braziliann National Council for Scientific and Technological Development (CNPq) and Ilha do Cardoso Plot is supported by São Paulo Research Foundation (FAPESP 2017/11979-9).

17. Jianfengling

This plot was supported by the National Natural Science Foundation of China (NSFC 31670628) and the Central Public-interest Scientific Institution Basal Research Fund (CAFYBB2017ZE001).

Han Xu, Yide Li, Mingxian Lin, Jianhui Wu, Tushou Luo, Zhang Zhou, Dexiang Chen, Huai Yang, Guangjian Li, Shirong Liu. Community characteristics of a 60 ha dynamics plot in the tropical montane rain forest in Jianfengling, Hainan Island. Biodiversity Science, 2015, 23 (2): 192–201.

18. Liangshui

NA

19. Luquillo

This work would not have been possible without the > 100 volunteers and staff that have assisted in the tree censuses of the LFDP. We thank the information management team in the Luquillo LTER office and the El Verde Field Station staff for their hard work and support. This research was supported by grants BSR-8811902, DEB 9411973, DEB 0080538, DEB 0218039, DEB 0620910, DEB 0963447, DEB-129764, DEB-1546686 AND DEB-1831952 from NSF to the Department of Environmental Science, University of Puerto Rico, and to the International Institute of Tropical Forestry, USDA Forest Service, as part of the Luquillo Long-Term Ecological Research Program. The U.S. Forest Service (Dept. of Agriculture) and the University of Puerto Rico gave additional support. The LFDP has also been supported by the Andrew Mellon foundation and the Smithsonian Institution Forest Global Earth Observatory.

20**. Muling**

This research was supported by the Fundamental Research Funds for Heilongjiang Province

(2018-01scz,2018-02scz) and the National Natural Science Foundation of Province (QC2018025, LH2020C104). Investigation: Yunfei Diao, Yulong Liu, Yunhong Li,Yingnan Shao, Lidong Han, Xiaotang Wo and Yao Chen.

21. Mulun

The Mulun forest plot was supported by the Guangxi Key Research and Development Program (AB17129009) and the National Natural Science Foundation of China (31870712)

22. Nabanhe

Funding for the establishment of the 20-ha Nabanhe Tropical Seasonal rainforest Dynamics Plot was provided by the Administrative Bureau of Naban River Watershed National Nature Reserve.

23. Nonggang

This research was supported by the National Natural Science Foundation of China (Grant No. 31660130, 31760131). We thank the field workers and volunteers who participated in the construction and vegetation censuses of the Nonggang plot. We gratefully acknowledge the support from the Administration Bureau of the Nongang National Nature Reserve.

24. Ngardok

The Ngardok forest dynamics monitoring plot is located within the Nature Reserve (NNR) of the Melekeok Conservation Network in the Republic of Palau. This forest dynamics plot is the first in the ForestGEO network to focus on forest recovery within an officially protected area. This type of plot will fill a very important gap in our understanding of tropical forests recovering from human disturbance. Ngardok Nature Reserve was the first of the Protected Area Network (PAN) sites for Palau and contains Lake Ngardok, the largest freshwater body in Micronesia, and is an internationally recognized Ramsar wetland site of international importance. The Ngardok forest dynamics monitoring plot is a collaboration of the USDA Forest Service-Institute of Pacific Islands Forestry, the Ngardok Nature Reserve of Melekeok State, and Palau Forestry under the Ministry of Natural Resources, the Environment and Tourism.

25. Ngel Nyaki

Support for the plot has been received from the Taraba State Forest Service, Chester Zoo, The A.G. Leventis Foundation, and Nexen Nigeria.

The setting up and enumeration of the ForestGEO plot in Ngel Nyaki, Nigeria was majorly funded by a donation from Retired General T.Y. Danjuma to the Nigerian Montane Forest Project and other smaller donations from Forest Global Earth Observatory (ForestGEO) of the Smithsonian Tropical Research Institute, Chester Zoo, England and the A.G. Leventis Foundation.

26. Nanjenshan

This work included dataset of the Nanjenshan Plot I (2.1 ha) which is sponsored by the Taiwan Forestry Bureau (108AS-10.7.1-FB-e1), Ministry of Science and Technology of Taiwan (MOST 108-2918-I-005-012) and Kenting National Park.

27. Qinling
This research was supported by the Youth Program of National Natural Science Foundation of China (Grant No. 32001171, 32001120)

28. Rabi
The Rabi 25-ha is a collaborative project of the National Center for Scientific and Technical Research (CENAREST) in Gabon, the Center for Conservation and Sustainability (CCS) of the Smithsonian Conservation Biology Institute (SCBI) and the Forest Global Earth Observatory (ForestGEO) of the Smithsonian Tropical Research Institute. Funding for the first census was provided by Shell Gabon, ForestGEO, and SCBI. Permission to conduct the field program in Gabon is provided by CENAREST. The plot is located in a conservation area of between a forest concession of the Compagnie des Bois du Gabon (CBG) and oil company Assala Gabon. This is contribution #200 of the Gabon Biodiversity Program.

Memiaghe, H. M., J. A. Lutz, L. Korte, A. Alonson, and D. Kenfack. 2016. Ecological importance of small-diameter trees to the structure, diversity, and biomass of a tropical evergreen forest at Rabi, Gabon. PLOS ONE 11(5): e0154988. http://dx.doi.org/10.1371/journal.pone.0154988

29. Santa Cruz
The UCSC Forest Ecology Research Plot was made possible by US National Science Foundation grants to Gregory S. Gilbert (DEB-0515520, DEB-084259, and DEB-1655896), by the Pepper-Giberson Chair Fund, the University of California, the UCSC Campus Natural Reserve, the US National Science Foundation grant DEB 1926438 to Kai Zhu, and the hard work of dozens of UCSC students. The plot project is part ForestGEO, a global network of large-scale demographic tree plots.

30. Smithsonian Conservation Biology Institute (SCBI)
Funding for the establishment of the SCBI ForestGEO Large Forest Dynamics Plot was provided by the Smithsonian Global Earth Observatory initiative, the Smithsonian Institution, National Zoological Park and the HSBC Climate Partnership. We especially thank the numerous technicians, interns and volunteers of the Conservation Ecology Center at the SCBI who were essential in assisting with plot establishment and data collection. Support for the original exclosure fence installation was provided by the Friends of the National Zoo and Earthwatch Foundation.

31. Scotty Creek
The Scotty Creek plot was supported by funds from the Natural Sciences and Engineering Research Council of Canada, Global Water Futures, Northern Water Futures, Canada Foundation for Innovation, Canada Foundation for Climate and Atmospheric Sciences, the Smithsonian Centre for Tropical Forest Science (ForestGEO), and Polar Knowledge Canada's Northern Scientific Training Program. We are grateful to Katherine Dearborn, Rajit Patankar, and Cory Wallace for their leadership in plot establishment and recensus and the hard work of many field assistants. Logistical support was provided through the Government of the Northwest Territories – Wilfrid Laurier University partnership and the Scotty Creek Research Station. We thank the Dehcho First Nations for permission to conduct research on their lands (Aurora Research Institute

license numbers 15413 and 16431).

Dearborn, KD, Wallace, CA, Patankar, R, Baltzer, JL. 2021. Permafrost thaw in boreal peatlands is rapidly altering forest community composition. Journal of Ecology, 109: 1452-1467. https://doi.org/10.1111/1365-2745.13569

32. Smithsonian Environmental Research Center (SERC)

These data were gathered as part of forest ecology studies at the Smithsonian Environmental Research Center (SERC). SERC is a participant in the Smithsonian Institution Forest Global Earth Observatory (ForestGEO) network.

33. Sherman

NA

34. Speulderbos

The Speulderbos Forest Dynamics Plot has been supported by Wageningen University.

35. Tiantongshan

Financial support for the Tiantongshan plot came from the National Natural Science Foundation of China (31870404 31470487 to G.S., 31210103920 to X.W.) and ECNU Multifunctional Platform for Innovation (008).

36. Tyson Research Center

The Tyson Research Center Forest Dynamics Plot (TRCP) is supported by Washington University in St. Louis' Tyson Research Center. Funding was provided by the International Center for Advanced Renewable Energy and Sustainability (I-CARES) at Washington University in St. Louis, National Science Foundation (DEB 1557094), Smithsonian Forest Global Earth Observatory (ForestGEO), and Tyson Research Center. We thank the Tyson Research Center staff for providing logistical support, and the more than 100 high school students, undergraduate students, and researchers that have contributed to the project. The TRCP is part of ForestGEO, a global network of large-scale forest dynamics plots.

LaManna, J. A., M. L. Walton, B. L. Turner, and J. A. Myers. 2016. Negative density dependence is stronger in resource-rich environments and diversifies communities when stronger for common but not rare species. Ecology Letters 19: 657-667.

Spasojevic, M. J., E. A. Yablon, B. Oberle, and J. A. Myers. 2014. Ontogenetic trait variation influences tree community assembly across environmental gradients. Ecosphere 5: article 129.

37. Uholka

The "Swiss-Ukrainian Research Plot" in Uholka is a collaborative project of the Swiss Federal

Research Institute WSL, the Carpathian Biosphere Reserve CBR and the Vasyl Stefanyk Precarpathian University.

Stillhard, J., M. Hobi, L. Hülsmann, P. Brang, C. Ginzler, M. Kabal, J. Nitzsche, G. Projer, Y. Shparyk, and B. Commarmot. 2019. Stand inventory data from the 10-ha forest research plot in Uholka: 15 yr of primeval beech forest development. Ecology 100(11):e02845. 10.1002/ecy.2845

38. Utah

The Utah Forest Dynamics Plot is a collaborative project of Utah State University and the Utah Agricultural Experiment Station (projects 1153 and 1398). We thank Cedar Breaks National Monument for providing logistical support, and the students, volunteers and staff individually listed at http://ufdp.org for data collection.

Furniss, T. J., A. J. Larson, and J. A. Lutz. 2017. Reconciling niches and neutrality in a subalpine temperate forest. Ecosphere 8(6): Article01847. https://doi.org/10.1002/ecs2.1847

Bishop, M., T. J. Furniss, K. E. Mock, and J. A. Lutz. 2019. Genetic and spatial structuring of Populus tremuloides in a mixed-species forest of southwest Utah, USA. Western North American Naturalist 79(1): 63-71. https://doi.org/10.3398/064.079.0107

39. Wanang

The 50-ha Wanang Forest Dynamics Plot is a collaborative project of the New Guinea Binatang Research Center, the Forest Global Earth Observatory (ForestGEO) of the Smithsonian Tropical Research Institute, the Forest Research Institute of Papua New Guinea, the Czech Academy of Sciences, and the University of Minnesota. It has received financial support from the US National Science Foundation (DEB-0816749), the Czech Science Foundation (20-17282S, 20-10205S), the Swire & Sons Ltd., Darwin Initiative for the Survival of Species (DIR25S1\100123), the European Research Council (669609) and the Christensen Foundation. We acknowledge the government of Papua New Guinea and the customary landowners of Wanang for supporting and maintaining the plot.

40. Wind River

The Wind River Forest Dynamics Plot is a collaborative project of Utah State University and the USDA Forest Service Pacific Northwest Research Station. Funding has been provided by the Smithsonian ForestGEO program, Utah State University, and the Utah Agricultural Experiment Station (projects 1153 and 1398). We acknowledge the Gifford Pinchot National Forest and the Pacific Northwest Wind River Field Station for providing logistical support, and the students, volunteers and staff individually listed at http://wfdp.org for data collection. The Wind River Forest Dynamics Plot was made possible by a grant from Jennifer Walston Johnson to the Smithsonian ForestGEO.

Lutz, J. A., A. J. Larson, J. A. Freund, M. E. Swanson, and K. J. Bible. 2013. The importance of large-diameter trees to forest structural heterogeneity. PLOS ONE 8(12): e82784.

https://doi.org/10.1371/journal.pone.0082784

Lutz, J. A., A. J. Larson, T. J. Furniss, J. A. Freund, M. E. Swanson, D. C. Donato, K. J. Bible, J. Chen, and J. F. Franklin. 2014. Spatially non-random tree mortality and ingrowth maintain equilibrium pattern in an old-growth *Pseudotsuga-Tsuga* forest. Ecology 95(8): 2047-2054. https://doi.org/10.1890/14-0157.1

41. Wytham Woods

The 18-ha Long-Term Forest Monitoring Plot is a collaborative project between the University of Oxford, the Centre for Ecology and Hydrology, and the Smithsonian Institution ForestGEO (HSBC Climate Partnership). The Wytham Forest Monitoring Plot is part of ForestGEO, a global network of large-scale demographic tree plots.
Lutz, Furniss et al. 2018

42. Xishuangbanna

This research was supported by the National Science Foundation of China (31370445，31570430,31570380, 31300358, 32061123003), the Natural Science Foundation of Yunnan Province (2015FB185), the Southeast Asia Biodiversity Research Institute, Chinese Academy of Sciences (2016CASSEABRIQG002).

43. Yosemite

The Yosemite Forest Dynamics Plot is a collaborative project of Utah State University, the University of Montana, and Washington State University. Funding has been provided by the Smithsonian ForestGEO, the National Park Service, the Joint Fire Science Program, Utah State University, and the Utah Agricultural Experiment Station (projects 1153 and 1398). We thank Yosemite National Park for providing logistical support, and the students, volunteers and staff individually listed at http://yfdp.org for data collection. The Yosemite Forest Dynamics Plot was made possible by a grant from Jennifer Walston Johnson to the Smithsonian ForestGEO.

Lutz, J. A., A. J. Larson, M. E. Swanson, and J. A. Freund. 2012. Ecological importance of large-diameter trees in a temperate mixed-conifer forest. PLoS ONE 7(5): e36131. https://10.1371/journal.pone.0036131

44. Yulongxueshan

This research was supported by the International Partnership Program of Chinese Academy of Sciences (151853KYSB20190027), the Strategic Priority Research Program of Chinese Academy of Sciences (XDB31000000) and Yunnan Lijiang Forest Ecosystem Observation and Research Station, China.

45. Zofin

This research was supported by Czech Science Foundation (20-17282S and 19-09427S). The Zofin Forest Dynamics Plot is part of the Forest Global Earth Observatory (ForestGEO), a worldwide network of large, long-term forest dynamics plots and was established with the support of Smithsonian Institution.

Janik, D., Vrška, T., Hort, L., Unar, P., Kral, K., 2018. Where have all the tree diameters grown? Patterns in Fagus sylvatica L. diameter growth on their run to the upper canopy. Ecosphere 9 (12), DOI: 10.1002/ecs2.2508.
