# Supplementary material for: Arbuscular mycorrhizal trees influence the latitudinal beta-diversity gradient of tree communities in forests worldwide
Source: Nat Commun. 2021 May 25;12:3137. doi: 10.1038/s41467-021-23236-3 (PMC8149669; doi:10.1038/s41467-021-23236-3)
Supplement: Supplementary file 2 — Reporting Summary [file 41467_2021_23236_MOESM2_ESM.pdf]

nature research

Corresponding author(s): Chengjin Chu

Last updated by author(s): Mar 13, 2021

Reporting Summary

Nature Research wishes to improve the reproducibility of the work that we publish. This form provides structure for consistency and transparency in reporting. For further information on Nature Research policies, see our Editorial Policies and the Editorial Policy Checklist.

Statistics

For all statistical analyses, confirm that the following items are present in the figure legend, table legend, main text, or Methods section.

n/a	Confirmed	
☐	☒	The exact sample size (*n*) for each experimental group/condition, given as a discrete number and unit of measurement
☐	☒	A statement on whether measurements were taken from distinct samples or whether the same sample was measured repeatedly
☐	☒	The statistical test(s) used AND whether they are one- or two-sided<br>*Only common tests should be described solely by name; describe more complex techniques in the Methods section.*
☐	☒	A description of all covariates tested
☐	☒	A description of any assumptions or corrections, such as tests of normality and adjustment for multiple comparisons
☐	☒	A full description of the statistical parameters including central tendency (e.g. means) or other basic estimates (e.g. regression coefficient) AND variation (e.g. standard deviation) or associated estimates of uncertainty (e.g. confidence intervals)
☐	☒	For null hypothesis testing, the test statistic (e.g. *F*, *t*, *r*) with confidence intervals, effect sizes, degrees of freedom and *P* value noted<br>*Give P values as exact values whenever suitable.*
☒	☐	For Bayesian analysis, information on the choice of priors and Markov chain Monte Carlo settings
☒	☐	For hierarchical and complex designs, identification of the appropriate level for tests and full reporting of outcomes
☒	☐	Estimates of effect sizes (e.g. Cohen's *d*, Pearson's *r*), indicating how they were calculated

Our web collection on statistics for biologists contains articles on many of the points above.

Software and code

Policy information about availability of computer code

Data collection	Climate data were extracted from global climate map from the Worldclim Database using R version 3.5.3 with the "raster" package version 2.6-7.
Data analysis	All analyses were conducted on R version 3.5.3 using packages "base" version 0.1-3, "betapart" version 1.5.0, "vegan" version 2.5-2, "Hmisc" version 4.2-0, "betareg" version 3.1-1, "randomForest" version 4.6-14, and "rfPermute" version 2.1.6. Custom codes for simulations are available in the Supplementary Information files.

For manuscripts utilizing custom algorithms or software that are central to the research but not yet described in published literature, software must be made available to editors and reviewers. We strongly encourage code deposition in a community repository (e.g. GitHub). See the Nature Research guidelines for submitting code & software for further information.

Data

Policy information about availability of data

All manuscripts must include a data availability statement. This statement should provide the following information, where applicable:

- Accession codes, unique identifiers, or web links for publicly available datasets
- A list of figures that have associated raw data
- A description of any restrictions on data availability

Full census data are available on reasonable request from the ForestGEO (https://www.forestgeo.si.edu/). Climatic variables including 19 bioclimatic variables and solar radiation are available from the WorldClim Database (http://worldclim.org/version2) and potential evapotranspiration and aridity index are available from the Global Aridity Index (Global-Aridity) and Global Potential Evapo-Transpiration (Global-PET) Geospatial Database (https://cgiarcsi.community/data/global-aridity-and-pet-database/).

Field-specific reporting

Please select the one below that is the best fit for your research. If you are not sure, read the appropriate sections before making your selection.

☐ Life sciences ☐ Behavioural & social sciences ☒ Ecological, evolutionary & environmental sciences

For a reference copy of the document with all sections, see nature.com/documents/nr-reporting-summary-flat.pdf

Ecological, evolutionary & environmental sciences study design

All studies must disclose on these points even when the disclosure is negative.

Study description	Data of 45 large forest plots across the globe were compiled. Trees were classified into AM-associated trees, EcM-associated trees, and other trees according to published database and literature. Beta regressions were used to detect the latitudinal patterns of beta diversity of different mycorrhizal-associated trees. Variation partitioning was conducted to test the relative importance of environmental and spatial variables, while random forest was used to test the relative importance of every single environmental variable.
Research sample	Tree census data are compiled from the ForestGEO (https://www.forestgeo.si.edu/). In each forest plot, all free-standing woody stems with a diameter at breast (DBH) ≥ 1 cm were identified to species, tagged, measured, and mapped. The Uholka plot in Ukraine was an exception as woody stems were censused from a DBH ≥ 6 cm. Tree census data comprise tree ID, latin name, diameter at breast height (DBH) in cm, growth form, growth status, and coordinates in the plot based on tree stem. There is also a dataset of elevation at the 10-m or 20-m resolution and the Universal Transverse Mercator (UTM) or WGS84 plot coordinates.
Sampling strategy	To account for the scale-dependency of beta-diversity patterns, plot-level data were divided into 10 m × 10 m, 20 m × 20 m, and 50 m × 50 m quadrat-level data. To control for the sampling effort and to facilitate comparison across plots, we randomly sampled 30 quadrats of 10 m × 10 m, 15 quadrats of 20 m × 20 m, and 15 quadrats of 50 m × 50 m in each plot, as plot size varied across forest plots which may influence beta-diversity. This sampling procedure was repeated 200 times for each quadrat size and the results were averaged for each plot with the 95% confidence interval (95% CI). As beta-diversity measure used in this study was calculated as the averaging value of pairwise distance between sampled quadrats for each plot, 15 quadrats generated 15*14/2=105 pairs of quadrats and consequent 105 distance values, a large sample size.
Data collection	Forest plot data were collected from the CTFS-ForestGEO network where forest plots were established and censused by different research teams but according to the same standard protocol which could be found in "Condit, R. 1998. Tropical forest census plots: methods and results from Barro Colorado Island, Panama and a comparison with other plots. Springer-Verlag andRG. Landes Company, Berlin andGeorgetown, TX".
Timing and spatial scale	Plot size ranges from 2.1 ha (Nanjenshan) to 60 ha (Jianfengling) and plot latitude ranges from 25.1°S (Ilha do Cardoso, Brazil) to 61.3°N (Scotty Creek, Canada), covering all continents with forests (i.e., Asia, Africa, Europe, South America, North America, and Oceania). Most plots especially those in China were established and censused near 2010 and they were re-censused every 5 years.
Data exclusions	At the 50 m × 50 m scale, plots smaller than 8 ha (Cocoli, Sherman, Nanjenshan, Ngardok) were excluded from the analyses to ensure adequate sample size and statistical power. This study focused only on trees. Data of other growth forms (such as shrubs and liana) were excluded because we wanted to test the latitudinal beta-diversity patterns of trees rather than all woody plants.
Reproducibility	We randomly sampled the same number of quadrats in each forest plot and repeated 200 times to ensure the robustness of our results. All attempts at replication were successful.
Randomization	Detailed forest plot data collection were conducted according to a standard protocol and could be found in "Condit, R. 1998. Tropical forest census plots: methods and results from Barro Colorado Island, Panama and a comparison with other plots. Springer-Verlag andRG. Landes Company, Berlin andGeorgetown, TX". We randomly sampled the same number of quadrats in each forest plot and repeated 200 times to ensure the robustness of our results. These sampling procedures are random.
Blinding	Blinding was not possible in this study because data were compiled from previously established and censused forest plots.

Did the study involve field work? ☐ Yes ☒ No

Reporting for specific materials, systems and methods

We require information from authors about some types of materials, experimental systems and methods used in many studies. Here, indicate whether each material, system or method listed is relevant to your study. If you are not sure if a list item applies to your research, read the appropriate section before selecting a response.

Materials & experimental systems

n/a	Involved in the study
☒	☐ Antibodies
☒	☐ Eukaryotic cell lines
☒	☐ Palaeontology and archaeology
☒	☐ Animals and other organisms
☒	☐ Human research participants
☒	☐ Clinical data
☒	☐ Dual use research of concern

Methods

n/a	Involved in the study
☒	☐ ChIP-seq
☒	☐ Flow cytometry
☒	☐ MRI-based neuroimaging
